# Supplementary material for: Single-cell aging trajectories reveal a dynamic coupling between nuclear size and proteasome concentration
Source: iScience. 2026 Jan 19;29(3):114736. doi: 10.1016/j.isci.2026.114736 (PMC12955095; doi:10.1016/j.isci.2026.114736)
Supplement: Document S1. Figures S1–S15 and Data S1 [file mmc1.pdf]

iScience, Volume *29*

Supplemental information

Single-cell aging trajectories reveal a dynamic coupling between nuclear size and proteasome concentration

Michael Mobaraki, Changhui Deng, Jiashun Zheng, and Hao Li

A

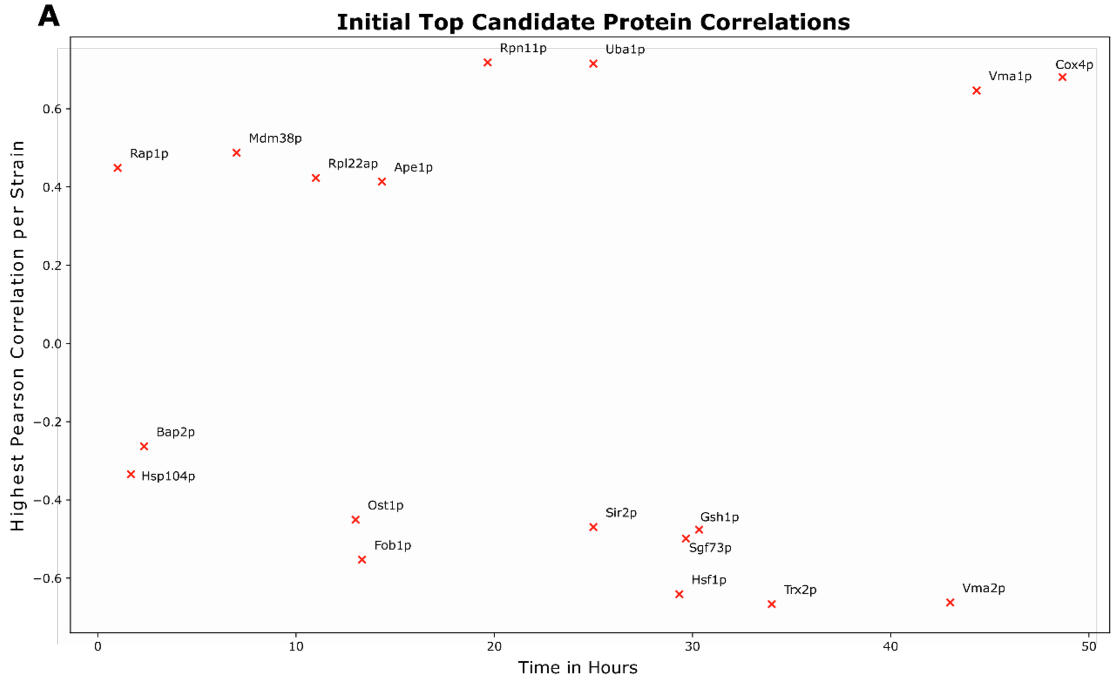

B

Proteostasis Related Proteins	Pearson Correlation	Time in Hours
Hsp104p	-0.35	2
Rpn11p	0.7	22
Uba1p	0.68	25
Hsf1p	-0.63	29
Trx2p	-0.65	35

Supplementary Fig. 1. Proteostasis related molecular markers are most predictive of lifespan. (**A**) The scatter plot displays the top 19 protein vs. lifespan correlations identified from a mini-screen of 34 GFP-tagged markers. Proteins with extreme phenotypes, poor signal quality, or insufficient sample sizes were excluded. Each red "X" represents a distinct protein, tagged at its C-terminal with GFP, for which Pearson correlation analysis was performed. The x-axis denotes the time (in hours) at which the maximum Pearson correlation with replicative lifespan (RLS) was observed. The y-axis represents the Pearson correlation coefficient at the time. A minimum of 20 dividing cells was analyzed for each correlation measurement, though sample size varied by strain. (**B**) A table summarizing notable proteostasis-related proteins that exhibited significant correlations with RLS. The Pearson correlation coefficient and corresponding time point (hours) at which the maximum correlation was observed are reported for each protein.

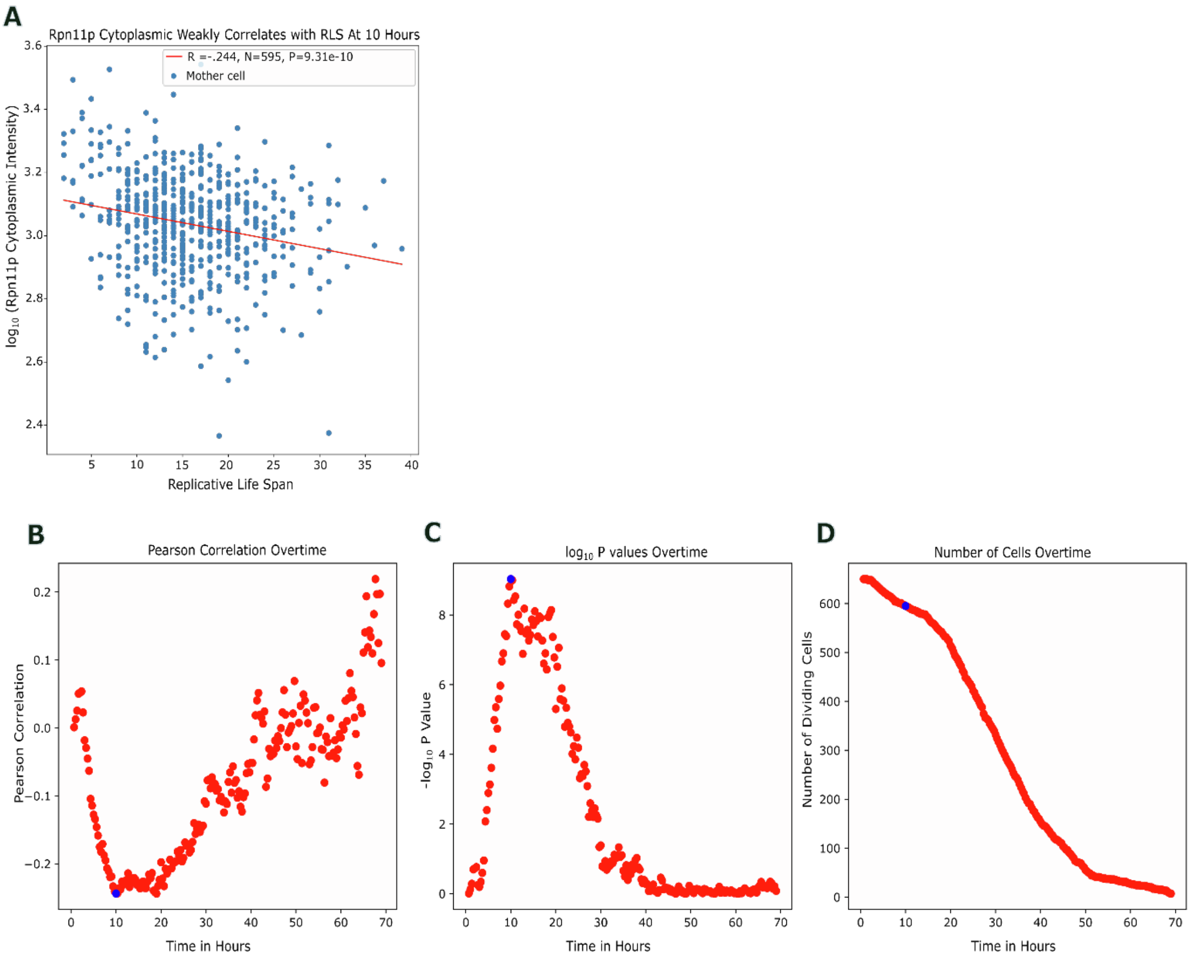

Supplementary Fig. 2. Cytoplasmic proteasome concentration weakly correlates with lifespan. (**A**) Scatter plot depicting the relationship between Rpn11p cytoplasmic intensity and replicative lifespan (RLS) at 10 hours. The x-axis represents the RLS of individual mother cells that were actively dividing at the 10-hour mark, while the y-axis shows the $\log_{10}$-transformed cytoplasmic intensity of Rpn11p at the same time point. A Pearson correlation coefficient of -0.244 was observed (N = 595 cells), with a p-value of 9.31e-10. The red regression line represents the trend in correlation. (**B**-**D**) Aggregated Rpn11p-mCherry data compiled from 12 independent experiments (N = 650 cells). Each experiment involved a different GFP-tagged protein, while Rpn11p-mCherry was consistently used as a reference marker. Only time points with ≥25 dividing cells were included in the analysis. (**B**) Pearson correlation of Rpn11p intensity over time. The x-axis denotes time (hours), and the y-axis represents the Pearson correlation coefficient between Rpn11p cytoplasmic intensity and RLS. (**C**) Statistical significance of correlation over time. The x-axis represents time (hours), while the y-axis shows the - $\log_{10}$-transformed p-values for the correlation at each time point. The highest statistical significance occurs at early time points, reaching the peak at ~10 hours, and gradually decreasing over time. (**D**) Number of dividing cells over time. The x-axis represents time (hours), while the y-axis shows the total number of live dividing cells at each time point. The blue dot marks the 10-hour time point corresponding to panel (A).

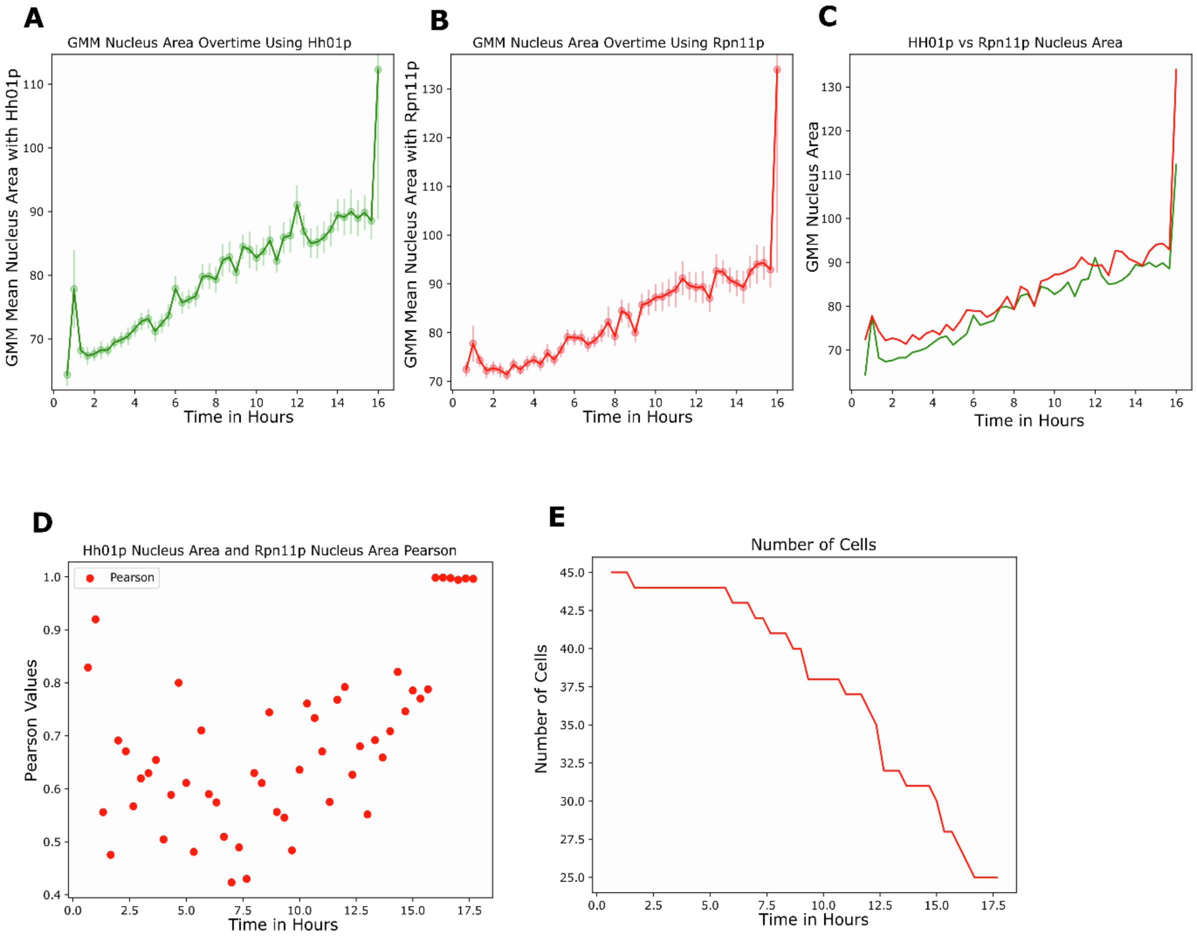

Supplementary Fig. 3. Nuclear areas derived from Rpn11p reporter and Hho1p reporter are highly correlated. (**A-B**) Mean nuclear area over time for Hho1p (green) and Rpn11p (red), respectively. The nuclear area was measured using a Gaussian Mixture Model (GMM)-based segmentation. The x-axis represents time (hours), while the y-axis represents the mean nuclear area (with standard error bars). (**C**) Comparison of nuclear area dynamics for Hho1p and Rpn11p. The nuclear area for both proteins is plotted over time, showing a similar trend in nuclear growth and segmentation. (**D**) Pearson correlation between Hho1p and Rpn11p nuclear area over time. The x-axis represents time, while the y-axis shows the Pearson correlation coefficient between the nuclear areas of the two proteins across single cells. (**E**) Number of dividing cells tracked over time. The x-axis represents time (hours), while the y-axis shows the number of actively dividing cells included in the analysis at each time point. The number of cells decreases as the experiment progresses. A total of 45 cells are tested.

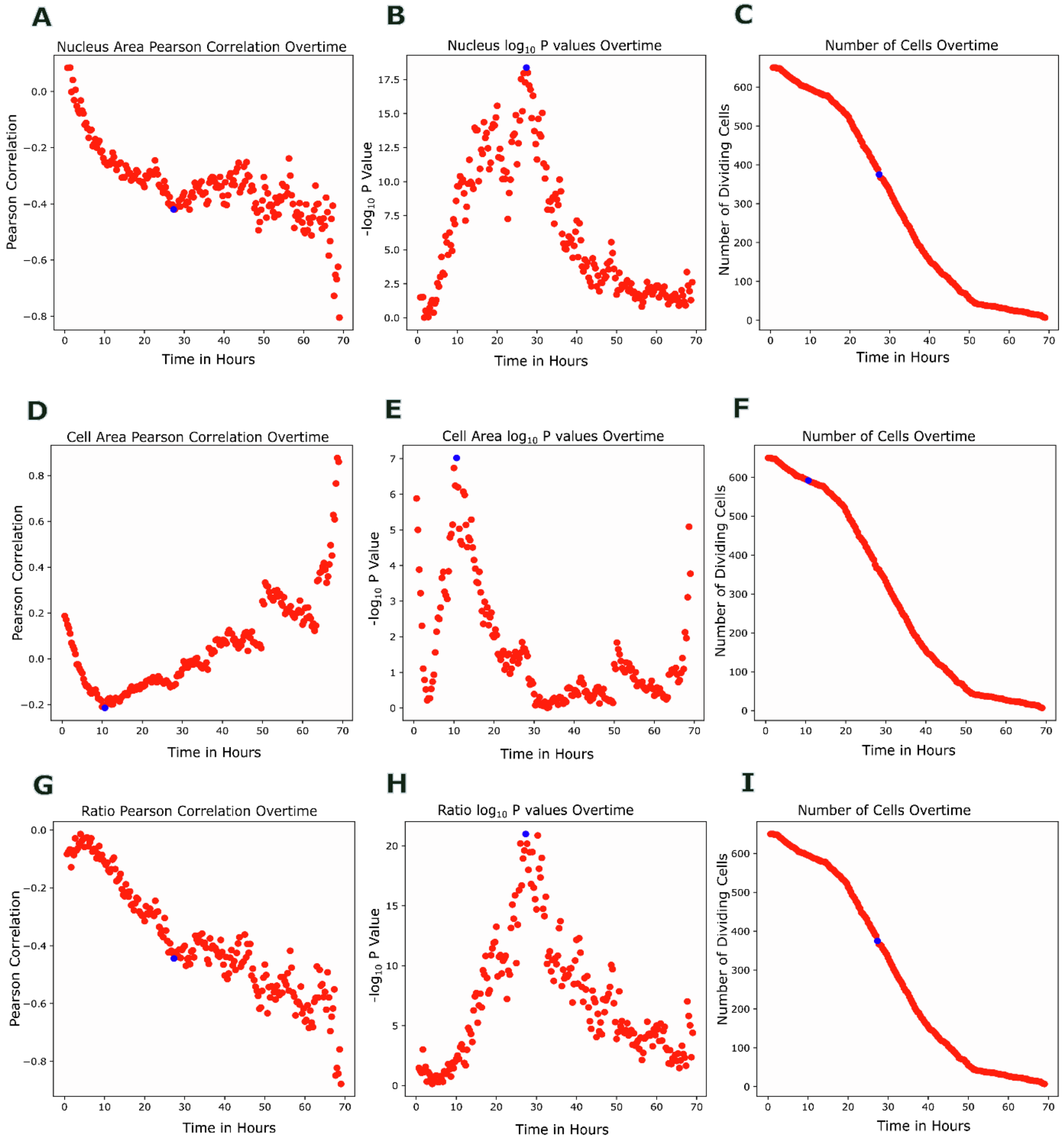

Supplementary Fig. 4. Correlation between cell area or nuclear area vs. lifespan. This figure presents Pearson correlations and statistical significance analyses of nuclear area, cell area, and their ratio vs. lifespan over time using nuclear Rpn11p-mCherry reference marker across 12 independent experiments (N = 650 cells). Rpn11p-mCherry was consistently used for reference and the nuclear region is identified using GMM. Only time points with ≥25 dividing cells were included in the analysis. (**A**-**C**) Correlation of nuclear area with RLS. Pearson correlation (**A**), the corresponding -$\log_{10}$ p-values (**B**) , and the number of actively dividing cells (**C**) as a function of time are shown. (**D**-**F**) Correlation of cell area with replicative lifespan. Pearson correlation (**D**), the corresponding -$\log_{10}$ p-values (**E**) , and the number of actively dividing cells (**F**) as a function of time are shown. (**G**-**I**) Correlation of nuclear-to-cell area ratio with RLS. Pearson correlation (**G**), the corresponding -$\log_{10}$ p-values (**H**), and the number of actively dividing cells (I) as a function of time are shown. Blue dots indicate times at which the -log10(P) reaches the maximum.

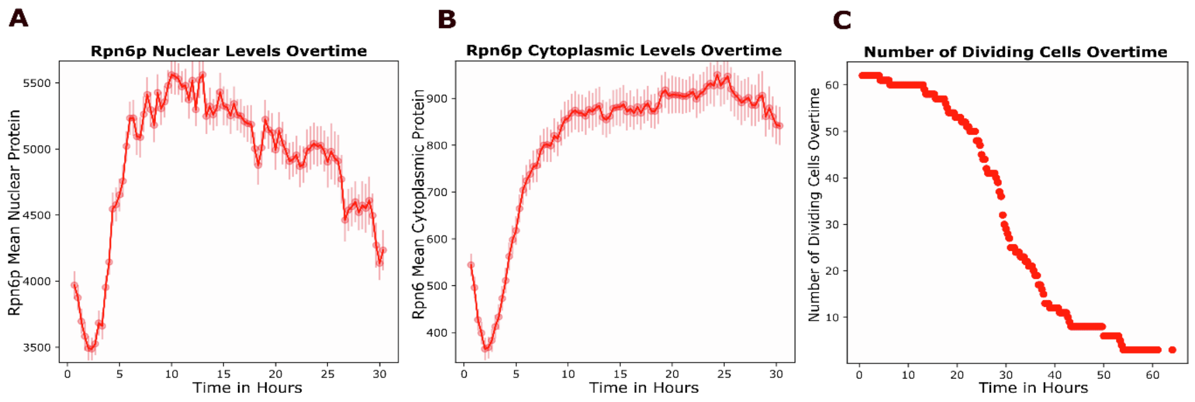

Supplementary Fig. 5. Rpn6p reporter displays similar dynamics as Rpn11p (**A**–**C**) Quantification of Rpn6p-mCherry localization over time in 62 dividing cells. Shown are nuclear Rpn6p intensity (**A**), cytoplasmic Rpn6p intensity (**B**), and number of dividing cells over time (**C**). Shaded error bars indicate the standard error of the mean (SEM). Nuclear and cytoplasmic intensities were extracted from single-cell tracking of Rpn6p-mCherry-expressing cells.

A

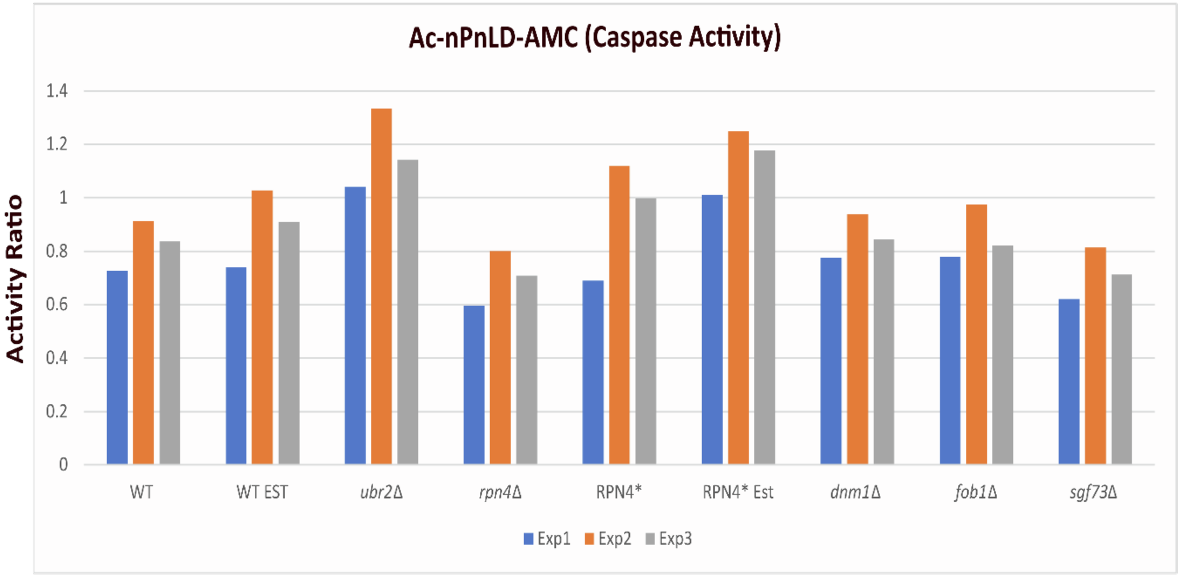

B

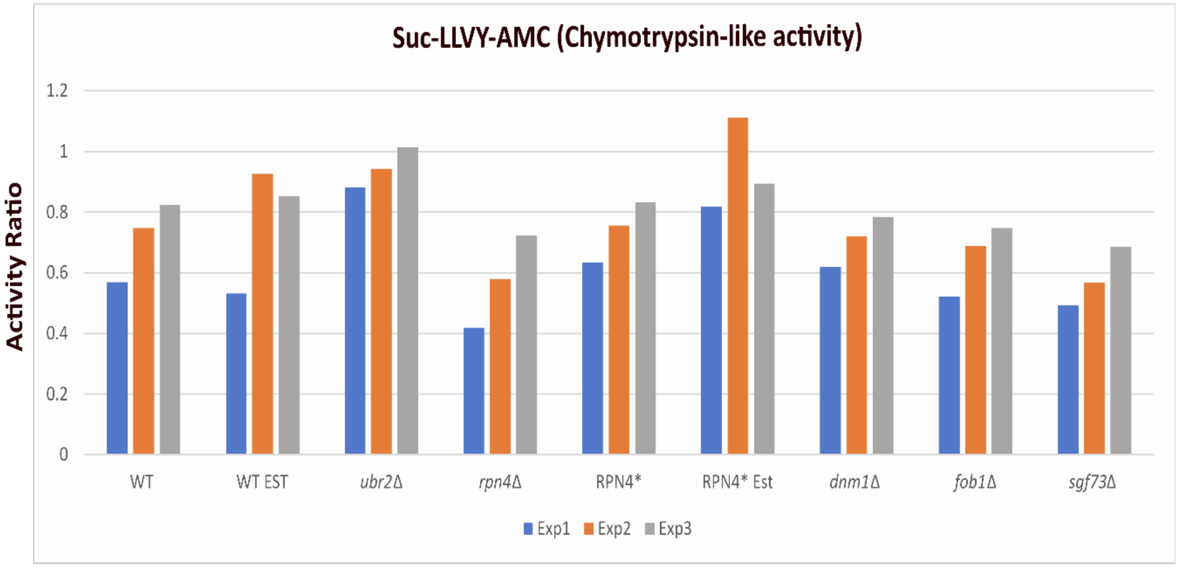

Supplementary Fig. 6. Proteasome activity measured for different strains. (A) Caspase-like proteasomal activity measured using the fluorogenic substrate Ac-nLPnLD-AMC. **(B)** Chymotrypsin-like proteasomal activity measured using the fluorogenic substrate Suc-LLVY-AMC. Strains tested include Wildtype (WT), WT treated with estradiol (WT EST), *ubr2Δ, rpn4Δ,* Rpn4*, Rpn4* treated with estradiol (RPN4* EST), *dnm1Δ*, *fob1Δ*, and *sfg73Δ*. For estradiol-treated samples, 16 nM estradiol was added for ~14 hours prior to harvesting. Cells were collected at an OD_{600} of 0.8–1.0, and 50 µg of total protein was assayed in the presence of 100 µM fluorogenic substrate. Proteasomal activity was measured in three independent experiments (Exp1–Exp3) and is presented as the activity ratio, calculated by comparing reactions with and without 50 µg/ml of the proteasome inhibitor MG132 to confirm specificity.

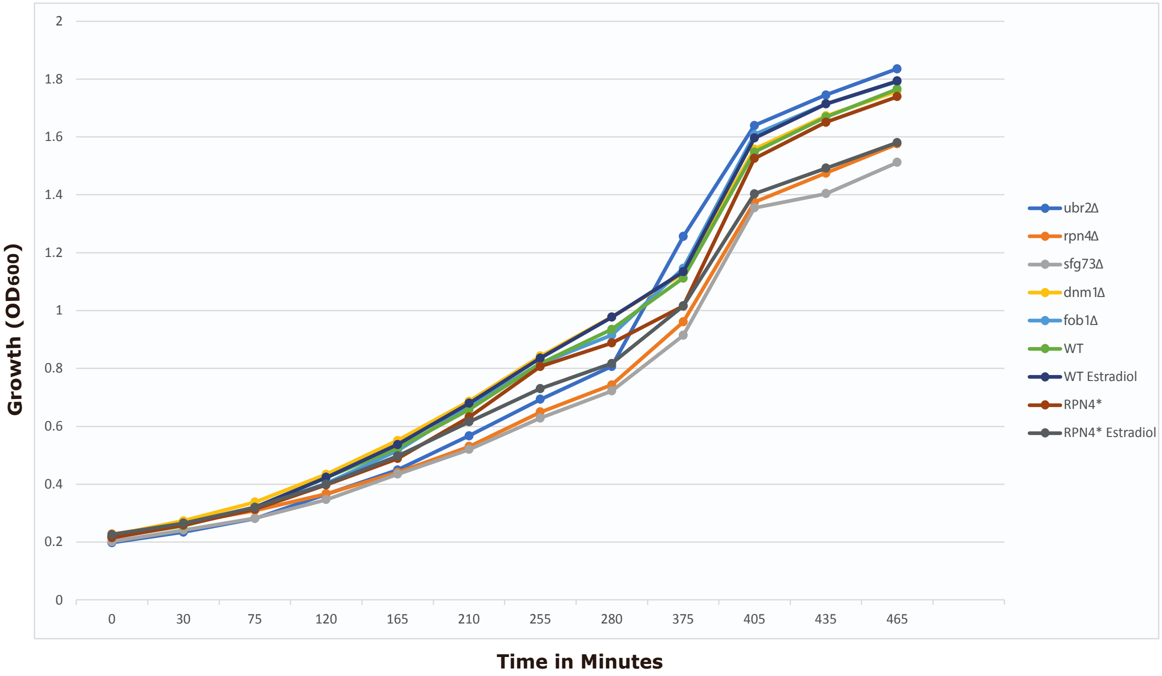

Supplementary Fig. 7. Growth curves for different strains. Growth measurements of *ubr2*Δ, *rpn4*Δ, *sfg73*Δ, *dnm1*Δ, *fob1*Δ, WT, WT estradiol, Rpn4*, and Rpn4* estradiol. Overnight cultures were diluted to an OD_{600} of ~0.2 in galactose rich media and grown near stationary phase. Cell density (OD_{600}) was measured at intervals as indicated and used to plot the growth curves. Samples given 16nM of estradiol were incubated during the start of the experiment.

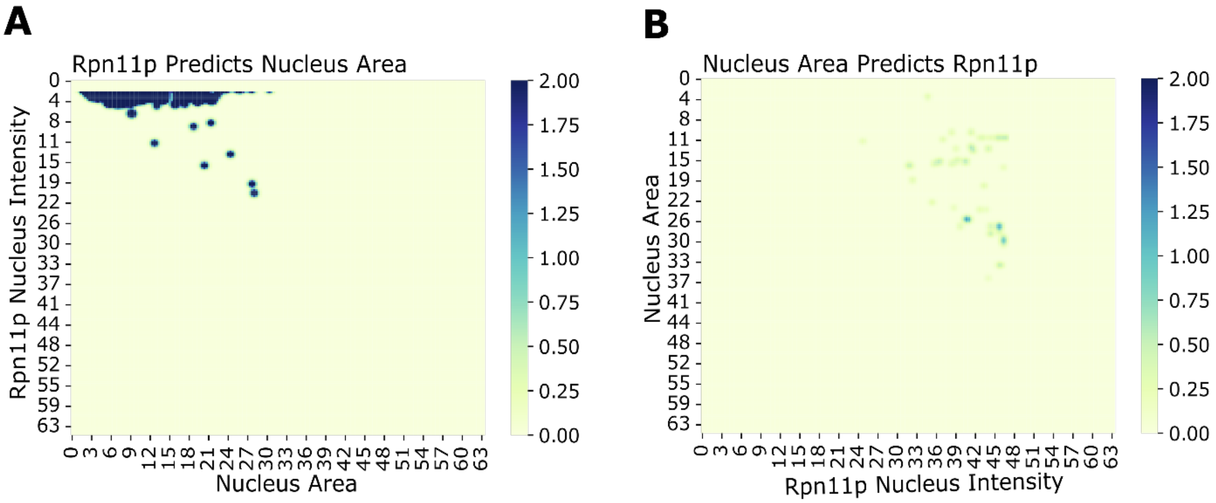

Supplementary Fig. 8. Predicting temporal order using Granger causality with decision trees. The following heatmaps display the -log10 P values calculated from an F-test. A Gaussian blur and threshold is used to filter background noise. (**A)** using Rpn11p nuclear intensity to predict nucleus area in later times. (**B**) using nuclear area to predict Rpn11p nucleus intensity in later times. The time points for both the y-axis and x-axis are the time in hours. Dataset is an accumulation of our Rpn11p-mCherry strains with ~650 cells. Time points with less than 56 dividing cells are excluded. Color scale indicates -log10(P) values.

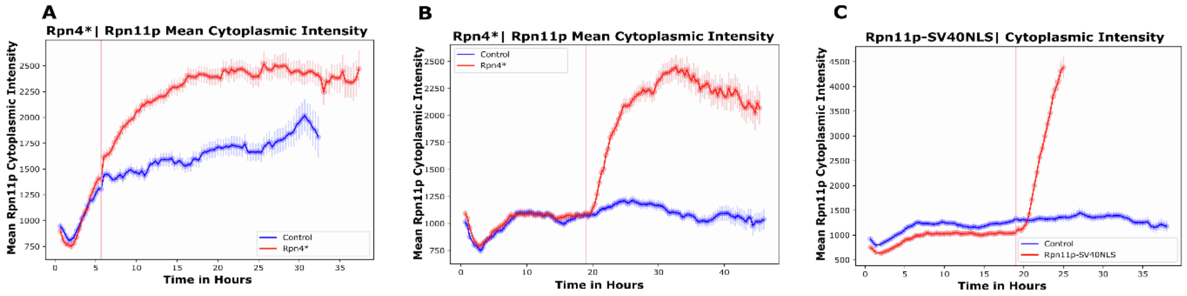

Supplementary Fig. 9. Dynamics of cytoplasmic Rpn11p intensity following Rpn4* and Rpn11-SV40NLS induction. (**A**) Time-course of mean cytoplasmic fluorescence intensity of Rpn11p in control cells (blue) versus cells expressing truncated Rpn4* (red) induced at 5.5 h (indicated by the red vertical line). (**B**) As in (**A**), but with induction at 19.2 h. (**C**) Time-course of mean cytoplasmic fluorescence intensity of Rpn11p in control cells (blue) versus cells being induced with Rpn11p–SV40NLS (red) at 19.2 h (red vertical line). Shaded areas denote SEM.

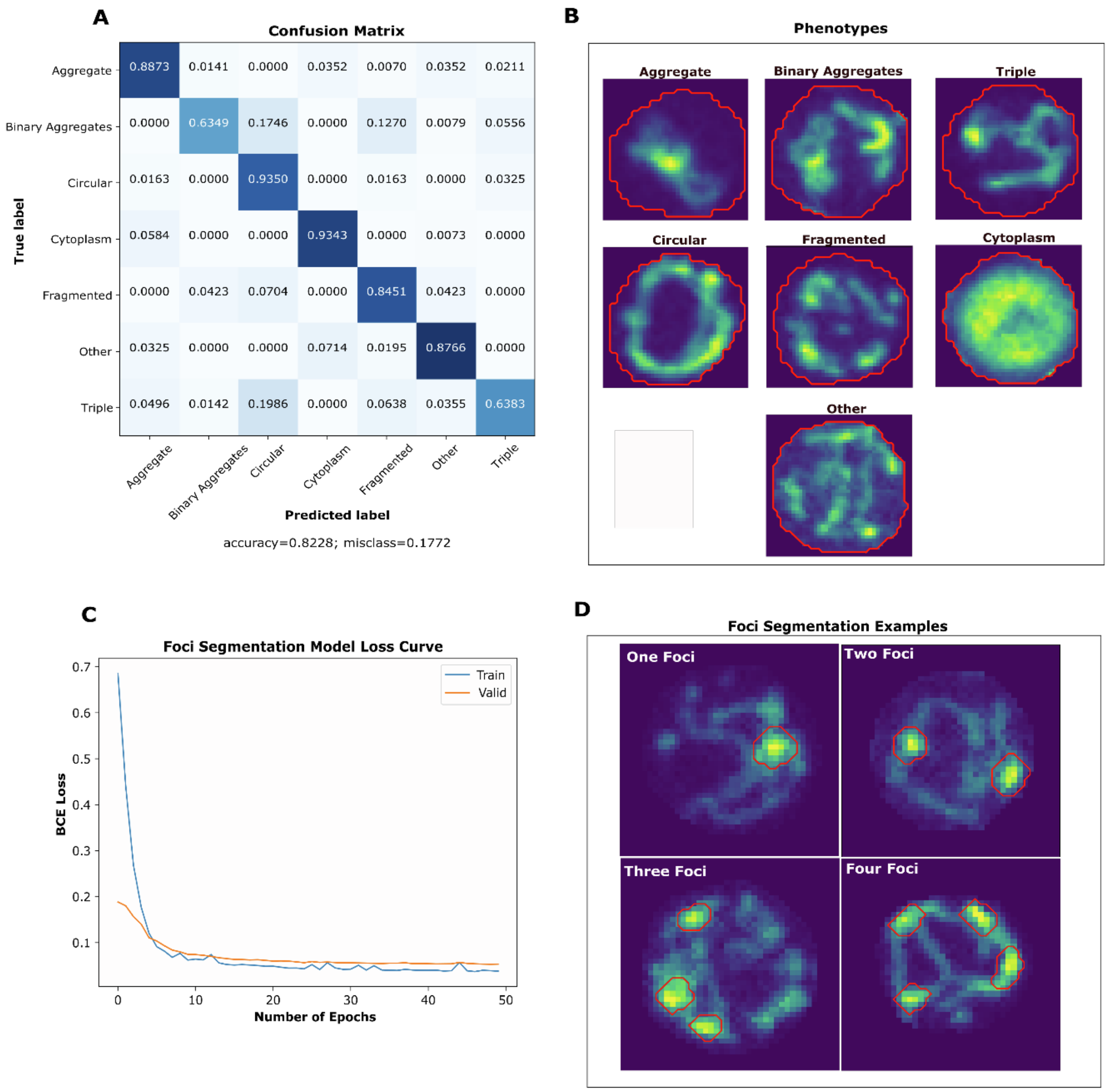

Supplementary Fig. 10. Classification of mitochondrial morphologies. (**A**) A confusion matrix of the ResNet50 model predictions for the validation dataset, with true labels on the y-axis and predicted labels on the x-axis. The validation dataset encompasses diverse experiments, integrating different protein organelle tags and knockout experiments. (**B**) Representative images of identified phenotypes. The red circle delineates the cell boarder. (**C**-**D**) relate to the Foci Segmentation Model. (**C**) the loss curve, with the training data depicted by the blue curve and the validation data by the orange curve. The y-axis signifies the Binary Cross-Entropy with Logits Loss (BCE), while the x-axis denotes the number of epochs. (**D** a selection of mitochondrial phenotypes with foci highlighted in red. The foci count per cell is outlined in white, ranging from one to four. The model can detect up to four or more foci.

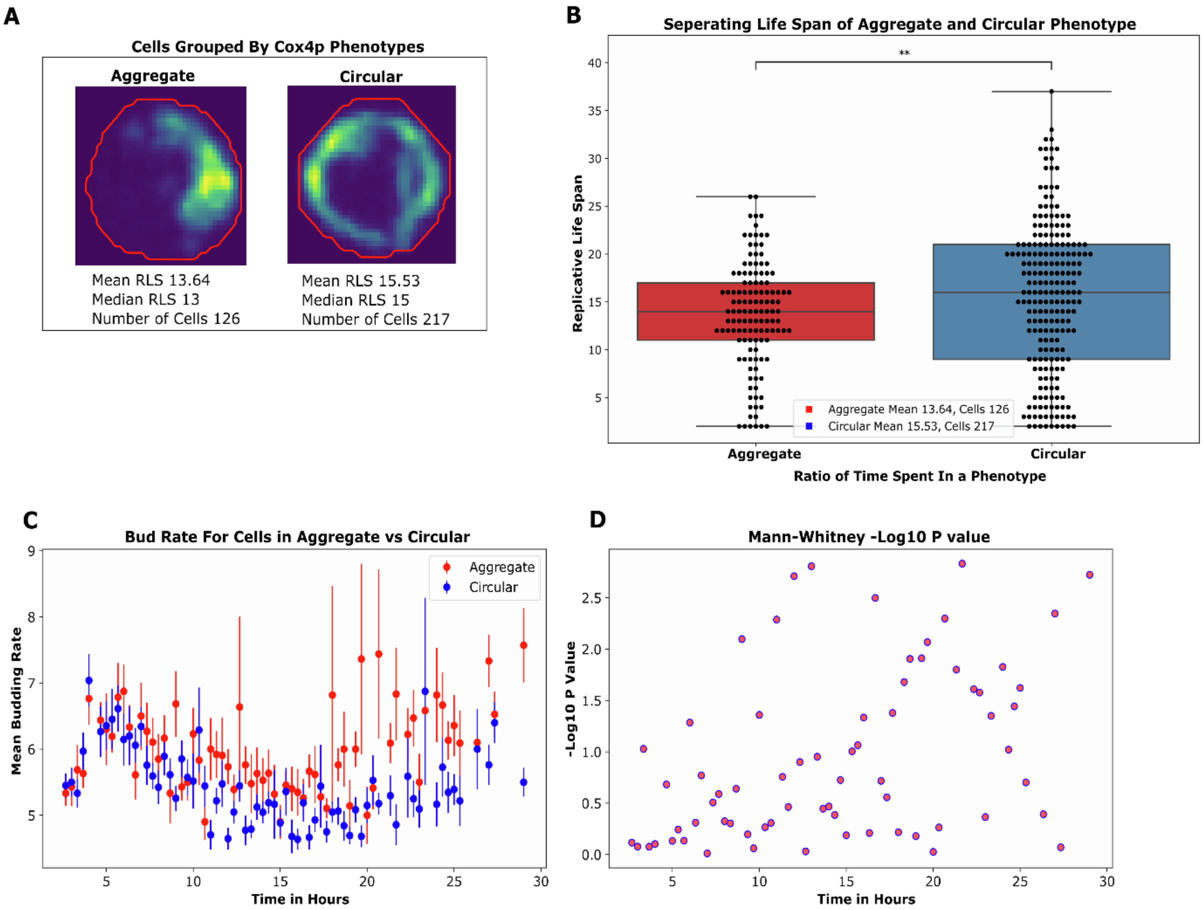

Supplementary Fig. 11. Cells with circular mitochondrial phenotype live longer and bud slower than those with aggregate phenotype. (**A**) example images of the aggregate and circular phenotype. For simplicity, cells displaying binary and triple phenotype are reclassified into the aggregate state. Cells displaying the fragmented state are recalssified into the circular state. This is decided by observing phenotype similarities to the aggregate or circular state. Next cells are grouped into aggregate or circular based on the time spent in the designated state. (**B**) a boxplot of the RLS for each cell classified in the aggregate or circular state. P value based on Mann-Whitney test is 9.605e-03. (**C**) the mean budding rate of cells with circular and aggregate phenotyes over time. Times with less than 10 dividing mother cells are excluded. The SEM of cells dividing at that time is depicted as error bars. (**D**) -log10 P values calculated using a Mann-whitney U test comparing the budding rate of cells calssified in the aggregate or circular state. The dataset consolidates our Cox4p-GFP and Rpn11p-mCherry strains from six independent experiments, encompassing 343 cells.

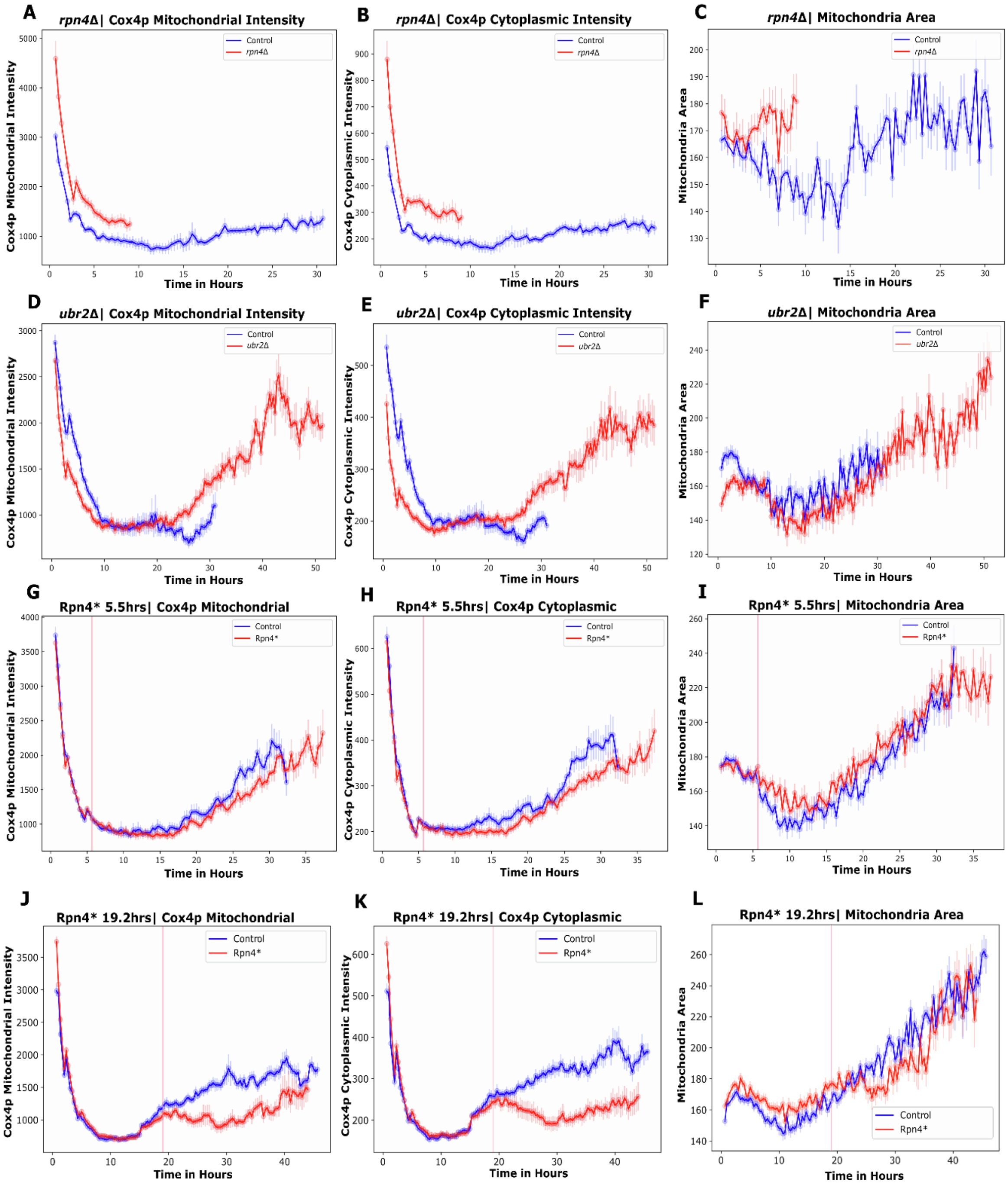

Supplementary Fig. 12. Response of Cox4p signal to various proteasome perturbations. The mean Cox4p mitochondrial intensity, mean Cox4p cytoplasmic intensity, and mean inner mitochondrial area of living dividing cells are shown for different proteasome perturbations of *rpn4*Δ (**A**-**C**), *ubr2*Δ (**D**-**F**), and temporal induction of Rpn4* at time 5.5hrs (**G**-**I**), and 19.2hrs (**J**-**L**). Only time points with a minimum of 15 cells are displayed. The blue curves represent the control, and the red curves represent the proteasome perturbation. SEM is displayed in the shaded areas within the curves. The pink line indicates when induction occurs. Both Rpn4* experiments were performed twice, and the data represents the accumulation of the separate experiments. Number of cells: For **A**-**C**, N= 47 for *rpn4*Δ and N=47 for the control. For **D**-**F**, N=61 for *ubr2*Δ and N= 46 for the control. For **G**-**I**, N=103 for

Rpn4* induction at 5.5 hours and N=107 for the control. For **J-L**, N= 115 for Rpn4* induction at time 19.2 hours and N=114 for the control.

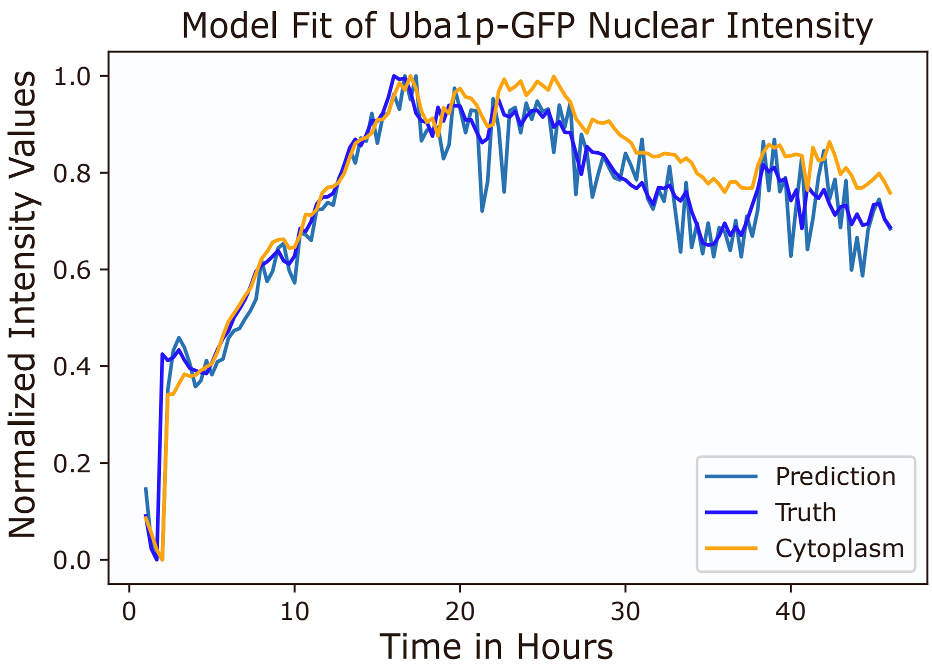

Supplementary Fig. 13. Dynamics of Uba1p intensity in nucleus (dark blue) and cytoplasm (orange). The light blue curve is the fit by the model,

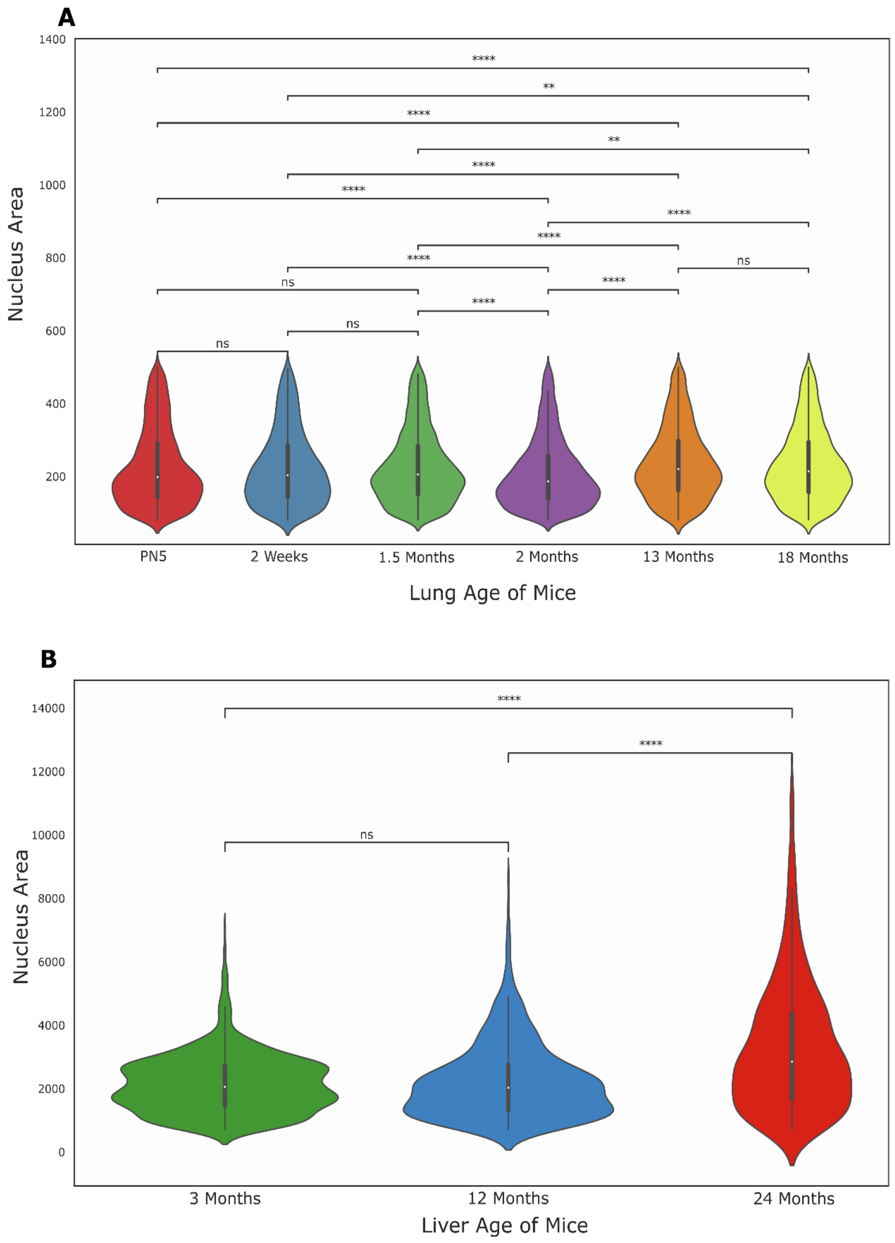

Supplementary Fig. 14. The nucleus area of old mouse lung and liver cells are larger than those in young mice. (**A**) Violin plot of nucleus area in lung tissue across different ages. Postnatal day 5 (PN5) and mice aged 2 weeks, 1.5 months, 2 months, 13 months, and 18 months are shown. The y-axis represents nucleus area, and the white dot denotes the median. The number of nuclei analyzed per group: PN5 (n = 6,016), 2 weeks (n = 2,568), 1.5 months (n = 13,041), 2 months (n = 9,288), 13 months (n = 3,385), and 18 months (n = 3,900). Statistical significance was determined using the Mann-Whitney U test, with $p < 0.05$ indicated by "*". (**B**) Violin plot of nucleus area in liver tissue across different ages. Mice aged 3, 12, and 24 months are shown. The y-axis represents nucleus area, and the white dot denotes the median. The number of nuclei analyzed per group: 3 months (n ≈ 1,034), 12 months (n = 693), and 24 months (n = 616). Statistical significance was determined using the Mann-Whitney U test, with $p < 0.05$ indicated by "*". The p-value for 12 vs. 24 months = 9.93×10^{-22}, and for 3 vs. 24 months = 8.25×10^{-29}.

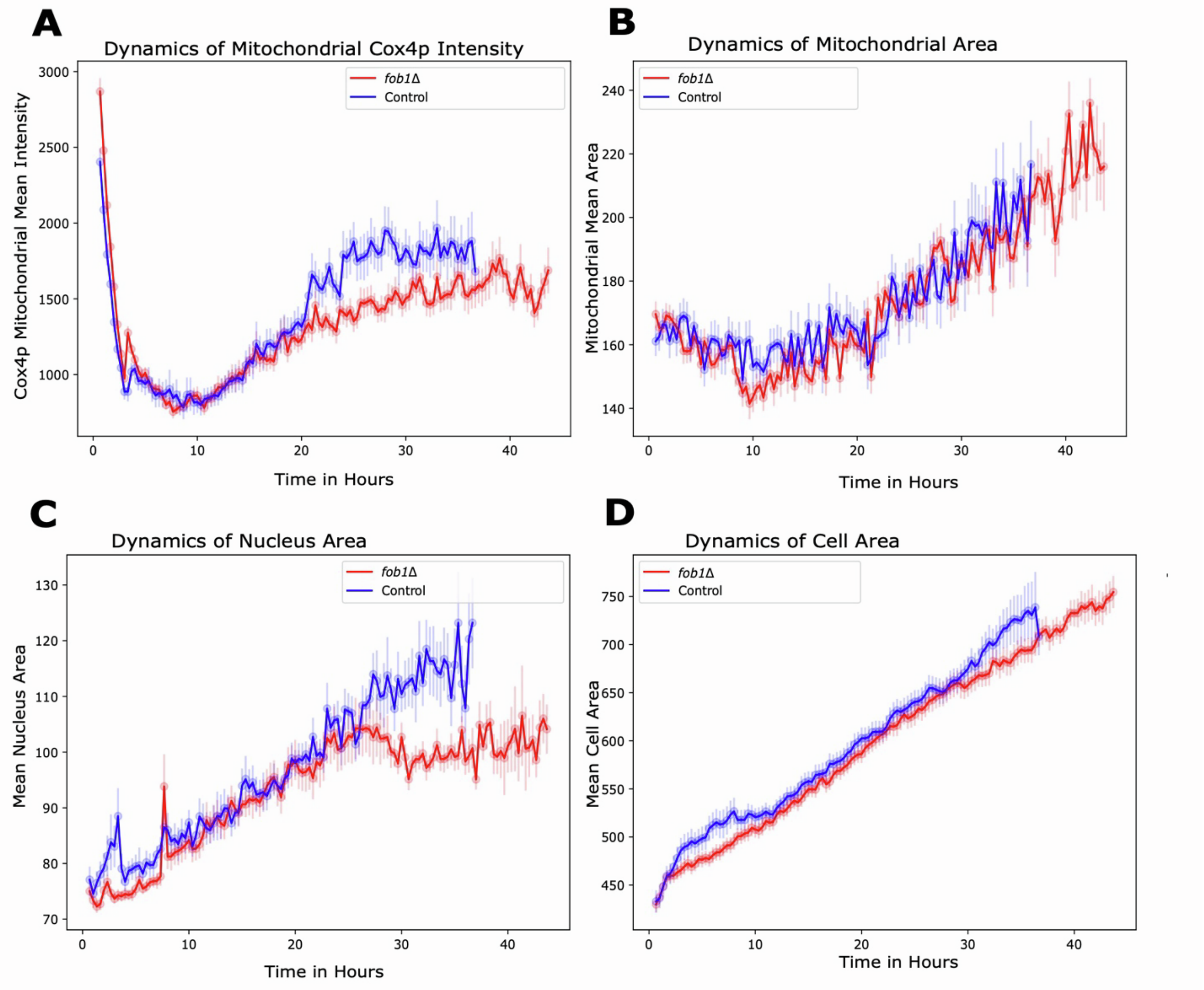

Supplementary Fig. 15. FOB1 deletion curbed nuclear size increase and decreased Cox4p intensity later in life. Cox4p intensity decreased in FOB1 deletion mutant compared to the WT (A), without change of mitochondrial size (B). The nuclear size stopped increase in FOB1 deletion mutant in later times (C), while cell size dynamics is unaffected (D).

Data S1: Plasmids used in this paper

Plasmid: HO_scgRNA_Ura3_CYS4_KAN_RET2_Bestra_GFP_Z4M for the estradiol systems

```
LOCUS       HO_scgRNA_Ura3_C       11599 bp    DNA     circular SYN 04-
NOV-2021
DEFINITION  synthetic circular DNA
ACCESSION   .
VERSION     .
KEYWORDS    .
SOURCE      synthetic DNA construct
  ORGANISM  synthetic DNA construct
REFERENCE   1  (bases 1 to 11599)
  AUTHORS   Trial User
  TITLE     Direct Submission
  JOURNAL   Exported Dec 22, 2025 from SnapGene 8.2.1
            https://www.snapgene.com
FEATURES             Location/Qualifiers
     source          1..11599
                     /mol_type="other DNA"
                     /organism="synthetic DNA construct"
     primer_bind     complement(6..22)
                     /label=M13 fwd
                     /note="common sequencing primer, one of multiple
similar
                     variants"
     primer_bind     6..22
                     /label=Leu2_NCoI_Forward_1
     rep_origin      163..618
                     /direction=RIGHT
                     /label=f1 ori
                     /note="f1 bacteriophage origin of replication; arrow
                     indicates direction of (+) strand synthesis"
     promoter        1256..1360
                     /gene="bla"
                     /label=AmpR promoter
     CDS             join(1361..1429,1430..2221)
                     /codon_start=1
                     /gene="bla"
                     /product="beta-lactamase"
                     /label=AmpR
                     /note="confers resistance to ampicillin,
carbenicillin, and
                     related antibiotics"

/translation="MSIQHFRVALIPFFAAFCLPVFAHPETLVKVKDAEDQLGARVGYI

ELDLNSGKILESFRPEERFPMMSTFKVLLCGAVLSRIDAGQEQLGRRIHYSQNDLVEYS

PVTEKHLTDGMTVRELCSAAITMSDNTAANLLLTTIGGPKELTAFLHNMGDHVTRLDRW

EPELNEAIPNDERDTTMPVAMATTLRKLLTGELLTLASRQQLIDWMEADKVAGPLLRSA
```

```
LPAGWFIADKSGAGERGSRGIIAALGPDGKPSRIVVIYTTGSQATMDERNRQIAEIGAS
                     LIKHW"
     rep_origin      2392..2980
                     /direction=RIGHT
                     /label=ori
                     /note="high-copy-number ColE1/pMB1/pBR322/pUC origin
of
                     replication"
     primer_bind     complement(3131..3152)
                     /label=RPR1_TETR_Reverse_4
     protein_bind    3268..3289
                     /label=CAP binding site
                     /bound_moiety="E. coli catabolite activator protein"
                     /note="CAP binding activates transcription in the
presence
                     of cAMP."
     promoter        join(3304..3309,3310..3327,3328..3334)
                     /label=lac promoter
                     /note="promoter for the E. coli lac operon"
     protein_bind    3342..3358
                     /label=lac operator
                     /bound_moiety="lac repressor encoded by lacI"
                     /note="The lac repressor binds to the lac operator
to
                     inhibit transcription in E. coli. This inhibition
can be
                     relieved by adding lactose or
                     isopropyl-beta-D-thiogalactopyranoside (IPTG)."
     primer_bind     3366..3382
                     /label=M13 rev
                     /note="common sequencing primer, one of multiple
similar
                     variants"
     primer_bind     3386..3402
                     /label=TOP_Check_Forward
     promoter        3403..3421
                     /label=T3 promoter
                     /note="promoter for bacteriophage T3 RNA polymerase"
     primer_bind     3429..3479
                     /label=HO_FSEI_Forward
     misc_feature    3458..3957

                     /note="URA3 forward for homology site cut with PMEI,
FSEI,
                     with NCOI "
     primer_bind     complement(3939..3966)
                     /label=NHEI_HO_Rever
     primer_bind     3956..3992
                     /label=NHEI_prestra_For
     misc_feature    3966..4190
                     /locus_tag="Sc tENO2"
                     /label=Sc tENO2
     primer_bind     4188..4208
```

```
                     /label=prestra_seq_for
                     /note="sequencing 2044bp"
     misc_feature    4195..4219
                     /locus_tag="Con1 scar"
                     /label=Con1 scar
     promoter        4224..4907
                     /locus_tag="pZ4 (-Gal4 site)"
                     /label=pZ4 (-Gal4 site)
     misc_feature    4456..4467
                     /locus_tag="Z4 Binding Site(2)"
                     /label=Z4 Binding Site
                     /label=Z4 Binding Site(2)
                     /label=nonstandard type: Binding
     misc_feature    4469..4480
                     /locus_tag="Z4 Binding Site(1)"
                     /label=Z4 Binding Site
                     /label=Z4 Binding Site(1)
                     /label=nonstandard type: Binding
     misc_feature    4483..4494
                     /locus_tag="Z4 Binding Site(3)"
                     /label=Z4 Binding Site
                     /label=Z4 Binding Site(3)
                     /label=nonstandard type: Binding
     misc_feature    4496..4507
                     /locus_tag="Z4 Binding Site(4)"
                     /label=Z4 Binding Site
                     /label=Z4 Binding Site(4)
                     /label=nonstandard type: Binding
     misc_feature    4510..4521
                     /locus_tag="Z4 Binding Site(5)"
                     /label=Z4 Binding Site
                     /label=Z4 Binding Site(5)
                     /label=nonstandard type: Binding
     misc_feature    4523..4534
                     /locus_tag="Z4 Binding Site"
                     /label=Z4 Binding Site
                     /label=nonstandard type: Binding
     misc_feature    complement(4547..4550)
                     /locus_tag="Bbs1(6)"
                     /label=Bbs1
                     /label=Bbs1(6)
     misc_feature    complement(4553..4558)
                     /locus_tag="Bbs1(4)"
                     /label=Bbs1
                     /label=Bbs1(4)
     promoter        4908..4920
                     /locus_tag="pZ4 (-Gal4 site)(1)"
                     /label=pZ4 (-Gal4 site)
                     /label=pZ4 (-Gal4 site)(1)
     promoter        4921..4947
                     /locus_tag="pZ4 (-Gal4 site)(2)"
                     /label=pZ4 (-Gal4 site)
                     /label=pZ4 (-Gal4 site)(2)
     primer_bind     complement(4922..4965)
```

```
                     /label=BSu36I_pestra_Rev
     primer_bind     4944..4995
                     /label=PMII_AVRII_Flour_For
     misc_feature    join(4948..4950,4951..4965)
                     /label=Sequence_insert
     misc_feature    4954..4959
                     /label=Sequencing_insert
     misc_feature    4966..4992
                     /label=Primer and Link
     primer_bind     4966..4986
                     /label=Forward
     misc_feature    4987..4996

     CDS             4993..5709
                     /codon_start=1
                     /product="Aequoria victoria green fluorescent
protein"
                     /label=GFP

/translation="MVSKGEELFTGVVPILVELDGDVNGHKFSVSGEGEGDATYGKLTL

KFICTTGKLPVPWPTLVTTFTYGVQCFSRYPDHMKRHDFFKSAMPEGYVQERTIFFKDD

GNYKTRAEVKFEGDTLVNRIELKGIDFKEDGNILGHKLEYNYNSHNVYIMADKQKNGIK

VNFKIRHNIEDGSVQLADHYQQNTPIGDGPVLLPDNHYLSTQSALSKDPNEKRDHMVLL
                     EFVTAAGITHGMDELYK"
     primer_bind     complement(5301..5325)
                     /label=Arg_SiteDirected_Muta_REV
     primer_bind     5301..5325
                     /label=Arg_SiteDirected_muta_For
     CDS             5320..5322
                     /codon_start=1
                     /product="Changed the argenine into a different
codon
                     sequence so PMII can be used "

                     /translation="R"
     promoter        5729..5747
                     /label=SP6 promoter
                     /note="promoter for bacteriophage SP6 RNA
polymerase"
     CDS             5761..5826
                     /codon_start=1
                     /product="three tandem FLAG(R) epitope tags,
followed by an
                     enterokinase cleavage site"
                     /label=3xFLAG
                     /translation="DYKDHDGDYKDHDIDYKDDDDK"
     CDS             5827..5844
                     /codon_start=1
                     /product="6xHis affinity tag"
                     /label=6xHis
                     /translation="HHHHHH"
```

```
     misc_feature    5859..6071
                     /label=KlLeu
                     /note="Portion is different from the URA3 plasmid"
     primer_bind     complement(6111..6139)
                     /label=SBFI_Flourecent_REV
     primer_bind     6128..6156
                     /label=SBFI_Estro_For
     misc_feature    6134..6135
                     /locus_tag="Con1 scar"
                     /label=Con1 scar
     misc_feature    6136..6835
                     /locus_tag="Sc pRET2"
                     /label=Sc pRET2
     misc_feature    6842..7192
                     /locus_tag="pZ4 DNA Binding Domain"
                     /label=pZ4 DNA Binding Domain
                     /label=nonstandard type:  ORF
     misc_feature    7223..8164
                     /locus_tag="hER-LBD"
                     /label=hER-LBD
     misc_feature    8198..9121
                     /locus_tag="Msn2 Activation Domain (27 more nt's, 
encoding
                     FPSMTNSRN)"
                     /label=Msn2 Activation Domain (27 more nt's, 
                     encoding ...
                     /label=Msn2 Activation Domain (27 more nt's, 
encoding
                     FPSMTNSRN)
                     /label=nonstandard type:  ORF
     primer_bind     complement(9102..9133)
                     /label=AFIII_Estro_RE_Rev
     primer_bind     complement(9102..9122)
                     /label=estra_seq_reverse
                     /note="3008bp"
     misc_feature    9127..9524
                     /label=tGMP1_Termin
     primer_bind     complement(9620..9647)
                     /label=RPR1_TETR_Reverse_4
     gene            9648..11004
                     /label=kanMX
                     /note="yeast selectable marker conferring kanamycin 
                     resistance (Wach et al., 1994)"
     promoter        9648..9991
                     /label=TEF promoter
                     /note="Ashbya gossypii TEF promoter"
     CDS             9992..10801
                     /codon_start=1
                     /gene="aph(3')-Ia"
                     /product="aminoglycoside phosphotransferase"
                     /label=KanR
                     /note="confers resistance to kanamycin"

/translation="MGKEKTHVSRPRLNSNMDADLYGYKWARDNVGQSGATIYRLYGKP
```

```
DAPELFLKHGKGSVANDVTDEMVRLNWLTEFMPLPTIKHFIRTPDDAWLLTTAIPGKTA

FQVLEEYPDSGENIVDALAVFLRRLHSIPVCNCPFNSDRVFRLAQAQSRMNNGLVDASD

FDDERNGWPVEQVWKEMHKLLPFSPDSVVTHGDFSLDNLIFDEGKLIGCIDVGRVGIAD
                     RYQDLAILWNCLGEFSPSLQKRLFQKYGIDNPDMNKLQFHLMLDEFF"
     terminator      10807..11004
                     /label=TEF terminator
                     /note="Ashbya gossypii TEF terminator"
     primer_bind     11005..11054
                     /label=HO_NOTI_For
     misc_feature    complement(11013..11031)
                     /label=T7 promoter
     primer_bind     complement(11013..11031)
                     /label=NAT_Reverse_5
                     /note="its been butched.
                     gaacttcgggcacagaatactttaatacgactcactatagggagac"
     misc_feature    11068..11567
                     /label=HO_End
                     /note="This will go downstream of the URA3 site and
remove
                     it
                     to insert the plasmid cut with NCOI with eitehr PMEI
or
                     FSEI. Or just use FSEI and PEMI"
     primer_bind     complement(11552..11599)
                     /label=HO_NCOI_Rev
     primer_bind     complement(11583..11599)
                     /label=End_Reverse_check
                     /note="To check sequence"
ORIGIN
        1 gcttcactgg ccgtcgtttt acaacgtcgt gactgggaaa accctggcgt
tacccaactt
       61 aatcgccttg cagcacatcc ccctttcgcc agctggcgta atagcgaaga
ggcccgcacc
      121 gatcgccctt cccaacagtt gcgcagcctg aatggcgaat ggacgcgccc
tgtagcggcg
      181 cattaagcgc ggcgggtgtg gtggttacgc gcagcgtgac cgctacactt
gccagcgccc
      241 tagcgcccgc tcctttcgct ttcttccctt cctttctcgc cacgttcgcc
ggctttcccc
      301 gtcaagctct aaatcggggg ctccctttag ggttccgatt tagtgcttta
cggcacctcg
      361 accccaaaaa acttgattag ggtgatggtt cacgtagtgg gccatcgccc
tgatagacgg
      421 tttttcgccc tttgacgttg gagtccacgt tctttaatag tggactcttg
ttccaaactg
      481 gaacaacact caaccctatc tcggtctatt cttttgattt ataagggatt
ttgccgattt
      541 cggcctattg gttaaaaaat gagctgattt aacaaaaatt taacgcgaat
tttaacaaaa
      601 tattaacgct tacaatttcc tgatgcggta ttttctcctt acgcatctgt
gcggtatttc
```

661 acaccgcata gggtaataac tgatataatt aaattgaagc tctaatttgt gagtttagta
721 ttaggatctc taccсttggc gaaaagtcct ctgccaacaa tgatgatatc tgatccacca
781 cttacaactt cgtcgacggt tctgtactgc tgacccaata tgcgtatata taccaatcta
841 agtctgtgct ccttccttcg ttcttccttc tgttcggaga ttaccgaatc aaaaaaattt
901 caaggaaacc gaaatcaaaa aaaagaataa aaaaaaaatg atgaattgaa aaggtggtat
961 ggtgcactct cagtacaatc tgctctgatg ccgcatagtt aagccagccc cgacacccgc
1021 caacacccgc tgacgcgccc tgacgggctt gtctgctccc ggcatccgct tacagacaag
1081 ctgtgaccgt ctccgggagc tgcatgtgtc agaggttttc accgtcatca ccgaaacgcg
1141 cgagacgaaa gggcctcgtg atacgcctat ttttataggt taatgtcatg ataataatgg
1201 tttcttagac gtcggcgcgc cgaggacgtc aggtggcact tttcggggaa atgtgcgcgg
1261 aacccctatt tgtttatttt tctaaataca ttcaaatatg tatccgctca tgagacaata
1321 accctgataa atgcttcaat aatattgaaa aaggaagagt atgagtattc aacatttccg
1381 tgtcgccctt attccctttt ttgcggcatt ttgccttcct gtttttgctc acccagaaac
1441 gctggtgaaa gtaaaagatg ctgaagatca gttgggtgca cgagtgggtt acatcgaact
1501 ggatctcaac agcggtaaga tccttgagag ttttcgcccc gaagaacgtt ttccaatgat
1561 gagcactttt aaagttctgc tatgtggcgc ggtattatcc cgtattgacg ccgggcaaga
1621 gcaactcggt cgccgcatac actattctca gaatgacttg gttgagtact caccagtcac
1681 agaaaagcat cttacggatg gcatgacagt aagagaatta tgcagtgctg ccataaccat
1741 gagtgataac actgcggcca acttacttct gacaacgatc ggaggaccga aggagctaac
1801 cgcttttttg cacaacatgg gggatcatgt aactcgcctt gatcgttggg aaccggagct
1861 gaatgaagcc ataccaaacg acgagcgtga caccacgatg cctgtagcaa tggcaacaac
1921 gttgcgcaaa ctattaactg gcgaactact tactctagct tcccggcaac aattaataga
1981 ctggatggag gcggataaag ttgcaggacc acttctgcgc tcggcccttc cggctggctg
2041 gtttattgct gataaatctg gagccggtga gcgtgggtct cgcggtatca ttgcagcact
2101 ggggccagat ggtaagccct cccgtatcgt agttatctac acgacgggga gtcaggcaac
2161 tatggatgaa cgaaatagac agatcgctga gataggtgcc tcactgatta agcattggta
2221 actgtcagac caagtttact catatatact ttagattgat ttaaaacttc atttttaatt

2281 taaaaggatc taggtgaaga tcctttttga taatctcatg accaaaatcc cttaacgtga
2341 gttttcgttc cactgagcgt cagaccccgt agaaaagatc aaaggatctt cttgagatcc
2401 tttttttctg cgcgtaatct gctgcttgca aacaaaaaaa ccaccgctac cagcggtggt
2461 ttgtttgccg gatcaagagc taccaactct ttttccgaag gtaactggct tcagcagagc
2521 gcagatacca aatactgttc ttctagtgta gccgtagtta ggccaccact tcaagaactc
2581 tgtagcaccg cctacatacc tcgctctgct aatcctgtta ccagtggctg ctgccagtgg
2641 cgataagtcg tgtcttaccg ggttggactc aagacgatag ttaccggata aggcgcagcg
2701 gtcgggctga acggggggtt cgtgcacaca gcccagcttg gagcgaacga cctacaccga
2761 actgagatac ctacagcgtg agctatgaga aagcgccacg cttcccgaag ggagaaaggc
2821 ggacaggtat ccggtaagcg gcagggtcgg aacaggagag cgcacgaggg agcttccagg
2881 gggaaacgcc tggtatcttt atagtcctgt cgggtttcgc cacctctgac ttgagcgtcg
2941 atttttgtga tgctcgtcag gggggcggag cctatggaaa aacgccagca acgcggcctt
3001 tttacggttc ctggcctttt gctggccttt tgctcacatg ttctttcctg cgttatcccc
3061 tgattctgtg gataaccgta ttaccgcctt tgagtgagct gataccgctc gccgcagccg
3121 aacgaccgag cgcagcgagt cagtgagcga ggaagcggaa gagcgcccaa tacgcaaacc
3181 gcctctcccc gcgcgttggc cgattcatta atgcagctgg cacgacaggt ttcccgactg
3241 gaaagcgggc agtgagcgca acgcaattaa tgtgagttag ctcactcatt aggcacccca
3301 ggctttacac tttatgcttc cggctcctat gttgtgtgga attgtgagcg gataacaatt
3361 tcacacagga aacagctatg accatgatta cgccaagctc ggaattaacc ctcactaaag
3421 ggaacaaaag ctgggccggc catttaaatg tttaaacgct aaattcgagt gaaacacagg
3481 aagatcagaa aatcctcatt tcatccatat taacaataat ttcaaatgtt tatttgcatt
3541 atttgaaact aggcaagaca agcaacgaaa cgtttttgaa aattttgagt attttcaata
3601 aatttgtaga ggactcagat attgaaaaaa agctacagca attaatactt gataagaaga
3661 gtattgagaa gggcaacggt tcatcatctc atggatctgc acatgaacaa acaccagagt
3721 caaacgacgt tgaaattgag gctactgcgc caattgatga caatacagac gatgataaca
3781 aaccgaagtt atctgatgta gaaaaggatt aaagatgcta agagatagtg atgatatttc
3841 ataaataatg taattctata tatgttaatt accttttttg cgaggcatat ttatggtgaa

3901 ggataagttt tgaccatcaa agaaggttaa tgtggctgtg gtttcagggt ccataaagcg
3961 ctagcagtgc ttttaactaa gaattattag tcttttctgc ttattttttc atcatagttt
4021 agaacacttt atattaacga atagtttatg aatctattta ggtttaaaaa ttgatacagt
4081 tttataagtt actttttcaa agactcgtgc tgtctattgc ataatgcact ggaaggggaa
4141 aaaaaaggtg cacacgcgtg gctttttctt gaatttgcag tttgaaaaat gctgccaaac
4201 cagatgtcaa cacagctaca acgttatatt gaattttcaa aaattcttac tttttttttg
4261 gatggacgca aagaagttta ataatcatat tacatggcat taccaccata tacatatcca
4321 tatacatatc catatctaat cttacttata tgttgtggaa atgtaaagag ccccattatc
4381 ttagcctaaa aaaaccttct ctttggaact ttcagtaata cgcttaactg ctcattgcta
4441 tattgaagtg tggccgcggc ggaggagtgc ggcggaggag gagcggcgga ggagtgcggc
4501 ggaggaggag cggcggagga gtgcggcgga ggagtctaga aattgcgtcc tcgtcttcac
4561 cggtcgcgtt cctgaaacgc agatgtgcct aatgccgcac tgctccgaac aataaagatt
4621 ctacaatact agcttttatg gttatgaaga ggaaaaattg gcagtaacct ggccccacaa
4681 accttcaaat taacgaatca aattaacaac cataggatga taatgcgatt agtttttttag
4741 ccttatttct ggggtaatta atcagcgaag cgatgatttt tgatctatta acagatatat
4801 aaatggaaaa gctgcataac cactttaact aatactttca acattttcag tttgtattac
4861 ttcttattca aatgtcataa aagtatcaac aaaaaattgt taatatacct ctatacttta
4921 acgtcaagga gaaaaaacta taagatccac gtgagctgcc ctagggatcc gctagcgcta
4981 ccggtcgcca ccatggtgag taaaggagaa gaacttttca ctggagttgt cccaattctt
5041 gttgaattag atggtgatgt taatgggcac aaattttctg tcagtggaga gggtgaaggt
5101 gatgcaacat acggaaaact tacccttaaa tttatttgca ctactggaaa actacctgtt
5161 ccatggccaa cacttgtcac tactttcact tatggtgttc aatgcttttc aagataccca
5221 gatcatatga aacggcatga ctttttcaag agtgccatgc ccgaaggtta tgtacaggaa
5281 agaactatat ttttcaaaga tgacgggaac tacaagacaa gagctgaagt caagtttgaa
5341 ggtgataccc ttgttaatag aatcgagtta aaaggtattg attttaaaga agatggaaac
5401 attcttggac acaaattgga atacaactat aactcacaca atgtatacat catggcagac
5461 aaacaaaaga atggaatcaa agttaacttc aaaattagac acaacattga agatggaagc

5521 gttcaactag cagaccatta tcaacaaaat actccaattg gcgatggccc
tgtcctttta
5581 ccagacaacc attacctgtc cacacaatct gccctttcga aagatcccaa
cgaaaagaga
5641 gaccacatgg tccttcttga gtttgtaaca gctgctggga ttacacatgg
catggatgaa
5701 ctatacaaat ccggactcag atcttacgat ttaggtgaca ctatagaacg
cggccgccag
5761 gactacaagg accatgacgg tgattacaag gatcatgaca tcgactacaa
ggatgacgat
5821 gacaagcatc atcaccatca ccatggttga ggcgcgcctc ctgcatcaaa
ttaaattgat
5881 taacctatca cattatttat cgtacatcaa tttactattg aaccattatt
tatttattac
5941 atactttgcc tagtatttaa gtcacattat ttcaagtcat aaccgtcatg
aagtcagttg
6001 agattgatta aatacagttc ttatgatcta aattacaaat tattactcca
atcaagactg
6061 aaaactggga aatccgctag ggataacagg gtaatatata agcttcgtac
gtgactgcag
6121 aatgcttcct gcaggacgat ggcttcttat ctcacttcaa tagtactttc
caccggttat
6181 acttccggct tttccctatt aatacaagct acaatttcaa tgggtggcaa
ataatgtgta
6241 gaatagaaaa taagccgaca gggtaataaa gaaaattttt agaaaaaaaa
ggttagatgg
6301 cttatttaag ttacaggcta gcgaaaaaag gaacttcagg gcaagtaaag
tgtttgattg
6361 ggcactagca tggcttataa aggcgagcaa ttgtcgaaac taattaatgt
tgtacggact
6421 attgctgtca tctcgtggta aatgcgtgtt ccaggtcgaa tactacttgc
acacaggcga
6481 gcggggcccc ataaaagtgt tgccgatttg ttaagttgtc ttttcggttt
ttctactctg
6541 ttattcctta cttccctttt taagaactct ttttatcctt catttaggat
cttgcacgtt
6601 tccgcctcat cacttgaatt aaaacatgtc tctgtcagta aaccttggcg
tttctattgt
6661 tcttcatagt tcaactttta ttattacccg ccctgcgcgt ttacattttt
ccagcaacag
6721 ccagcgaaaa attagaaaat ctggttgttg acacctcaag aacaagggca
attagcctca
6781 gcgtcgaata tagatcatat tagaatacct atagctccat caaaagaaat
acacaagatc
6841 tatgggtacc cgcccatatg cttgccctgt cgagtcctgc gatcgccgct
tttctcgcca
6901 cgccaatctt acccgccata tccgcatcca taccggtcag aagcccttcc
agtgtcgaat
6961 ctgcatgcgt aacttcagtc gtaatgcgaa ccttgtgcgc cacatccgca
cccacacagg
7021 atcccaaaag ccgttccaat gtcggatctg tatgcggaac tttagtcgaa
aggccgacct
7081 gaggcgtcac attcgcacgc acaccggcga gaagcctttt gcctgtgaca
tttgtgggag

7141 gaagtttgcc aggaagggcg acctcaagag gcataccaaa atccatacag gtagatccgg
7201 tgacggtgct ggtttaatta actctgctgg agacatgaga gctgccaacc tttggccaag
7261 cccgctcatg atcaaacgct ctaagaagaa cagcctggcc ttgtccctga cggccgacca
7321 gatggtcagt gccttgttgg atgctgagcc ccccatactc tattccgagt atgatcctac
7381 cagacccttc agtgaagctt cgatgatggg cttactgacc aacctggcag acagggagct
7441 ggttcacatg atcaactggg cgaagagggt gccaggcttt gtggatttga ccctccatga
7501 tcaggtccac cttctagaat gtgcctggct agagatcctg atgattggac tcgtctggcg
7561 ctccatggag cacccaggga agctactgtt tgctcctaac ttgctcttgg acaggaacca
7621 gggaaaatgt gtagagggca tggtggagat cttcgacatg ctgctggcta catcatctcg
7681 gttccgcatg atgaatctgc agggagagga gtttgtgtgc ctcaaatcta ttattttgct
7741 taattctgga gtgtacacat ttctgtccag caccctgaag tctctggaag agaaggacca
7801 tatccaccga gtcctggaca agatcacaga cactttgatc cacctgatgg ccaaggcagg
7861 cctgaccctg cagcagcagc accagcggct ggcccagctc ctcctcatcc tctcccacat
7921 caggcacatg agtaacaaag gcatggagca tctgtacagc atgaagtgca agaacgtggt
7981 gcccctctat gacctgctgc tggagatgct ggacgcccac cgcctacatg cgcccactag
8041 ccgtggaggg gcatccgtgg aggaaacgga ccaaagccac ttggccactg cgggctctac
8101 ttcatcgcat tccttgcaaa agtattacat cacgggggag gcagagggtt tccctgccac
8161 agtcgcggct gcaggtgacg gtgctggttt aattaacatg acggtcgacc atgatttcaa
8221 tagcgaagat attttattcc ccatagaaag catgagtagt atacaatacg tggagaataa
8281 taacccaaat aatattaaca acgatgttat cccgtattct ctagatatca aaaacactgt
8341 cttagatagt gcggatctca atgacattca aaatcaagaa acttcactga atttggggct
8401 tcctccacta tctttcgact ctccactgcc cgtaacggaa acgataccat ccactaccga
8461 taacagcttg catttgaaag ctgatagcaa caaaaatcgc gatgcaagaa ctattgaaaa
8521 tgatagtgaa attaagagta ctaataatgc tagtggctct ggggcaaatc aatacacaac
8581 tcttacttca ccttatccta tgaacgacat tttgtacaac atgaacaatc cgttacaatc
8641 accgtcacct tcatcggtac ctcaaaatcc gactataaat cctcccataa atacagcaag
8701 taacgaaact aatttatcgc ctcaaacttc aaatggtaat gaaactctta tatctcctcg

8761 agcccaacaa catacgtcca ttaaagataa tcgtctgtcc ttacctaatg gtgctaattc
8821 gaatcttttc attgacacta acccaaacaa tttgaacgaa aaactaagaa atcaattgaa
8881 ctcagataca aattcatatt ctaactccat ttctaattca aactccaatt ctacgggtaa
8941 tttaaattcc agttatttta attcactgaa catagactcc atgctagatg attacgtttc
9001 tagtgatctc ttattgaatg atgatgatga tgacactaat ttatcacgcc gaagatttag
9061 cgacgttata acaaaccaat ttccgtcaat gacaaattcg aggaatgagc tcggatccta
9121 acttaagtct gaagaatgaa tgatttgatg atttcttttt ccctccattt ttcttactga
9181 atatatcaat gatatagact tgtatagttt attatttcaa attaagtagc tatatatagt
9241 caagataacg tttgtttgac acgattacat tattcgtcga catctttttt cagcctgtcg
9301 tggtagcaat ttgaggagta ttattaattg aataggttca ttttgcgctc gcataaacag
9361 ttttcgtcag ggacagtatg ttggaatgag tggtaattaa tggtgacatg acatgttata
9421 gcaataacct tgatgtttac atcgtagttt aatgtacacc ccgcgaattc gttcaagtag
9481 gagtgcacca attgcaaagg gaaaagctga atgggcagtt cgaaccgcgg tctttcctgc
9541 gttatcccct gattctgtgg ataaccgtat taccgccttt gagtgagctg ataccgctcg
9601 ccgcagccga acgaccgagc gcagcgagtc agtgagcgag gctgagcgac atggaggccc
9661 agaataccct ccttgacagt cttgacgtgc gcagctcagg ggcatgatgt gactgtcgcc
9721 cgtacattta gcccatacat ccccatgtat aatcatttgc atccatacat tttgatggcc
9781 gcacggcgcg aagcaaaaat tacggctcct cgctgcagac ctgcgagcag ggaaacgctc
9841 ccctcacaga cgcgttgaat tgtccccacg ccgcgcccct gtagagaaat ataaaaggtt
9901 aggatttgcc actgaggttc ttctttcata tacttccttt taaaatcttg ctaggataca
9961 gttctcacat cacatccgaa cataaacaac catgggtaag gaaaagactc acgtttcgag
10021 gccgcgatta aattccaaca tggatgctga tttatatggg tataaatggg ctcgcgataa
10081 tgtcgggcaa tcaggtgcga caatctatcg attgtatggg aagcccgatg cgccagagtt
10141 gtttctgaaa catggcaaag gtagcgttgc caatgatgtt acagatgaga tggtcagact
10201 aaactggctg acggaattta tgcctcttcc gaccatcaag cattttatcc gtactcctga
10261 tgatgcatgg ttactcacca ctgcgatccc cggcaaaaca gcattccagg tattagaaga
10321 atatcctgat tcaggtgaaa atattgttga tgcgctggca gtgttcctgc gccggttgca

10381 ttcgattcct gtttgtaatt gtccttttaa cagcgatcgc gtatttcgtc
tcgctcaggc
10441 gcaatcacga atgaataacg gtttggttga tgcgagtgat tttgatgacg
agcgtaatgg
10501 ctggcctgtt gaacaagtct ggaaagaaat gcataagctt ttgccattct
caccggattc
10561 agtcgtcact catggtgatt tctcacttga taaccttatt tttgacgagg
ggaaattaat
10621 aggttgtatt gatgttggac gagtcggaat cgcagaccga taccaggatc
ttgccatcct
10681 atggaactgc ctcggtgagt tttctccttc attacagaaa cggctttttc
aaaaatatgg
10741 tattgataat cctgatatga ataaattgca gtttcatttg atgctcgatg
agtttttcta
10801 atcagtactg acaataaaaa gattcttgtt ttcaagaact tgtcatttgt
atagtttttt
10861 tatattgtag ttgttctatt ttaatcaaat gttagcgtga tttatatttt
ttttcgcctc
10921 gacatcatct gcccagatgc gaagttaagt gcgcagaaag taatatcatg
cgtcaatcgt
10981 atgtgaatgc tggtcgctat actggcggcc gccctatagt gagtcgtatt
agagttatta
11041 cccgagtaga gcacttgaat ccactgcccc gggaatctcg gtcgtaatga
tttctataat
11101 gacgaaaaaa aaaaaattgg aaagaaaaag cttcatggcc tttataaaaa
ggaactatcc
11161 aatacctcgc cagaaccaag taacagtatt ttacggggca caaatcaaga
acaataagac
11221 aggactgtaa agatggacgc attgaactcc aaagaacaac aagagttcca
aaaagtagtg
11281 gaacaaaagc aaatgaagga tttcatgcgt ttgtactcta atctggtaga
aagatgtttc
11341 acagactgtg tcaatgactt cacaacatca aagctaacca ataaggaaca
aacatgcatc
11401 atgaagtgct cagaaaagtt cttgaagcat agcgaacgtg tagggcagcg
tttccaagaa
11461 caaaacgctg ccttgggaca aggcttgggc cgataaggtg tactggcgta
tatatatcta
11521 attatgtatc tctggtgtag cccattttta gcatgtaaat ataaagaccg
agcgggccgg
11581 cctcgggcca tggtcactg
//

Plasmid: HO_scgRNA_Ura3_CYS4_KAN_RET2_Bestra_RPN4_Z4M for inducible RPN4

```
LOCUS       HO_scgRNA_Ura3_C       12275 bp    DNA     circular SYN 09-
FEB-2022
DEFINITION  synthetic circular DNA
ACCESSION   .
VERSION     .
KEYWORDS    .
SOURCE      synthetic DNA construct
  ORGANISM  synthetic DNA construct
REFERENCE   1  (bases 1 to 12275)
  AUTHORS   Trial User
  TITLE     Direct Submission
  JOURNAL   Exported Dec 22, 2025 from SnapGene 8.2.1
            https://www.snapgene.com
FEATURES             Location/Qualifiers
     source          1..12275
                     /mol_type="other DNA"
                     /organism="synthetic DNA construct"
     primer_bind     complement(6..22)
                     /label=M13 fwd
                     /note="common sequencing primer, one of multiple
similar
                     variants"
     primer_bind     6..22
                     /label=Leu2_NCoI_Forward_1
     rep_origin      163..618
                     /direction=RIGHT
                     /label=f1 ori
                     /note="f1 bacteriophage origin of replication; arrow
                     indicates direction of (+) strand synthesis"
     promoter        1256..1360
                     /gene="bla"
                     /label=AmpR promoter
     CDS             join(1361..1429,1430..2221)
                     /codon_start=1
                     /gene="bla"
                     /product="beta-lactamase"
                     /label=AmpR
                     /note="confers resistance to ampicillin,
carbenicillin, and
                     related antibiotics"

/translation="MSIQHFRVALIPFFAAFCLPVFAHPETLVKVKDAEDQLGARVGYI

ELDLNSGKILESFRPEERFPMMSTFKVLLCGAVLSRIDAGQEQLGRRIHYSQNDLVEYS

PVTEKHLTDGMTVRELCSAAITMSDNTAANLLLTTIGGPKELTAFLHNMGDHVTRLDRW

EPELNEAIPNDERDTTMPVAMATTLRKLLTGELLTLASRQQLIDWMEADKVAGPLLRSA

LPAGWFIADKSGAGERGSRGIIAALGPDGKPSRIVVIYTTGSQATMDERNRQIAEIGAS
                     LIKHW"
     rep_origin      2392..2980
```

```
                     /direction=RIGHT
                     /label=ori
                     /note="high-copy-number ColE1/pMB1/pBR322/pUC origin
of
                     replication"
     primer_bind     complement(3131..3152)
                     /label=RPR1_TETR_Reverse_4
     protein_bind    3268..3289
                     /label=CAP binding site
                     /bound_moiety="E. coli catabolite activator protein"
                     /note="CAP binding activates transcription in the
presence
                     of cAMP."
     promoter        join(3304..3309,3310..3327,3328..3334)
                     /label=lac promoter
                     /note="promoter for the E. coli lac operon"
     protein_bind    3342..3358
                     /label=lac operator
                     /bound_moiety="lac repressor encoded by lacI"
                     /note="The lac repressor binds to the lac operator
to
                     inhibit transcription in E. coli. This inhibition
can be
                     relieved by adding lactose or
                     isopropyl-beta-D-thiogalactopyranoside (IPTG)."
     primer_bind     3366..3382
                     /label=M13 rev
                     /note="common sequencing primer, one of multiple
similar
                     variants"
     primer_bind     3386..3402
                     /label=TOP_Check_Forward
     promoter        3403..3421
                     /label=T3 promoter
                     /note="promoter for bacteriophage T3 RNA polymerase"
     primer_bind     3429..3479
                     /label=HO_FSEI_Forward
     misc_feature    3458..3957

                     /note="URA3 forward for homology site cut with PMEI,
FSEI,
                     with NCOI "
     primer_bind     complement(3939..3966)
                     /label=NHEI_HO_Rever
     primer_bind     3956..3992
                     /label=NHEI_prestra_For
     misc_feature    3966..4190
                     /locus_tag="Sc tENO2"
                     /label=Sc tENO2
     primer_bind     4188..4208
                     /label=prestra_seq_for
                     /note="sequencing 2044bp"
     misc_feature    4195..4219
                     /locus_tag="Con1 scar"
```

```
                     /label=Con1 scar
     promoter        4224..4907
                     /locus_tag="pZ4 (-Gal4 site)"
                     /label=pZ4 (-Gal4 site)
     misc_feature    4456..4467
                     /locus_tag="Z4 Binding Site(2)"
                     /label=Z4 Binding Site
                     /label=Z4 Binding Site(2)
                     /label=nonstandard type: Binding
     misc_feature    4469..4480
                     /locus_tag="Z4 Binding Site(1)"
                     /label=Z4 Binding Site
                     /label=Z4 Binding Site(1)
                     /label=nonstandard type: Binding
     misc_feature    4483..4494
                     /locus_tag="Z4 Binding Site(3)"
                     /label=Z4 Binding Site
                     /label=Z4 Binding Site(3)
                     /label=nonstandard type: Binding
     misc_feature    4496..4507
                     /locus_tag="Z4 Binding Site(4)"
                     /label=Z4 Binding Site
                     /label=Z4 Binding Site(4)
                     /label=nonstandard type: Binding
     misc_feature    4510..4521
                     /locus_tag="Z4 Binding Site(5)"
                     /label=Z4 Binding Site
                     /label=Z4 Binding Site(5)
                     /label=nonstandard type: Binding
     misc_feature    4523..4534
                     /locus_tag="Z4 Binding Site"
                     /label=Z4 Binding Site
                     /label=nonstandard type: Binding
     misc_feature    complement(4547..4550)
                     /locus_tag="Bbs1(6)"
                     /label=Bbs1
                     /label=Bbs1(6)
     misc_feature    complement(4553..4558)
                     /locus_tag="Bbs1(4)"
                     /label=Bbs1
                     /label=Bbs1(4)
     promoter        4908..4920
                     /locus_tag="pZ4 (-Gal4 site)(1)"
                     /label=pZ4 (-Gal4 site)
                     /label=pZ4 (-Gal4 site)(1)
     promoter        4921..4947
                     /locus_tag="pZ4 (-Gal4 site)(2)"
                     /label=pZ4 (-Gal4 site)
                     /label=pZ4 (-Gal4 site)(2)
     primer_bind     complement(4922..4953)
                     /label=BSu36I_pestra_Rev
     primer_bind     4944..4980
                     /label=RPN4_PMII_Kozak_for
     misc_feature    4948..4950
```

```
                     /label=Sequence_insert
     misc_feature    4954..4963
                     /label=Kozak_sequence
     misc_feature    4960..6565
                     /label=RPN4
     primer_bind     complement(6768..6815)
                     /label=RPN4_SBFI_AVRII_Rev
     primer_bind     6804..6832
                     /label=SBFI_Estro_For
     misc_feature    6810..6811
                     /locus_tag="Con1 scar"
                     /label=Con1 scar
     misc_feature    6812..7511
                     /locus_tag="Sc pRET2"
                     /label=Sc pRET2
     misc_feature    7518..7868
                     /locus_tag="pZ4 DNA Binding Domain"
                     /label=pZ4 DNA Binding Domain
                     /label=nonstandard type:  ORF
     misc_feature    7899..8840
                     /locus_tag="hER-LBD"
                     /label=hER-LBD
     misc_feature    8874..9797
                     /locus_tag="Msn2 Activation Domain (27 more nt's,
encoding
                     FPSMTNSRN)"
                     /label=Msn2 Activation Domain (27 more nt's,
                     encoding ...
                     /label=Msn2 Activation Domain (27 more nt's,
encoding
                     FPSMTNSRN)
                     /label=nonstandard type:  ORF
     primer_bind     complement(9778..9809)
                     /label=AFIII_Estro_RE_Rev
     primer_bind     complement(9778..9798)
                     /label=estra_seq_reverse
                     /note="3008bp"
     misc_feature    9803..10200
                     /label=tGMP1_Termin
     primer_bind     complement(10296..10323)
                     /label=RPR1_TETR_Reverse_4
     gene            10324..11680
                     /label=kanMX
                     /note="yeast selectable marker conferring kanamycin
                     resistance (Wach et al., 1994)"
     promoter        10324..10667
                     /label=TEF promoter
                     /note="Ashbya gossypii TEF promoter"
     CDS             10668..11477
                     /codon_start=1
                     /gene="aph(3')-Ia"
                     /product="aminoglycoside phosphotransferase"
                     /label=KanR
                     /note="confers resistance to kanamycin"
```

```
/translation="MGKEKTHVSRPRLNSNMDADLYGYKWARDNVGQSGATIYRLYGKP

DAPELFLKHGKGSVANDVTDEMVRLNWLTEFMPLPTIKHFIRTPDDAWLLTTAIPGKTA

FQVLEEYPDSGENIVDALAVFLRRLHSIPVCNCPFNSDRVFRLAQAQSRMNNGLVDASD

FDDERNGWPVEQVWKEMHKLLPFSPDSVVTHGDFSLDNLIFDEGKLIGCIDVGRVGIAD
                     RYQDLAILWNCLGEFSPSLQKRLFQKYGIDNPDMNKLQFHLMLDEFF"
     terminator      11483..11680
                     /label=TEF terminator
                     /note="Ashbya gossypii TEF terminator"
     primer_bind     11681..11730
                     /label=HO_NOTI_For
     misc_feature    complement(11689..11707)
                     /label=T7 promoter
     primer_bind     complement(11689..11707)
                     /label=NAT_Reverse_5
                     /note="its been butched.
                     gaacttcgggcacagaatactttaatacgactcactatagggagac"
     misc_feature    11744..12243
                     /label=HO_End
                     /note="This will go downstream of the URA3 site and
remove
                     it
                     to insert the plasmid cut with NCOI with eitehr PMEI
or
                     FSEI. Or just use FSEI and PEMI"
     primer_bind     complement(12228..12275)
                     /label=HO_NCOI_Rev
     primer_bind     complement(12259..12275)
                     /label=End_Reverse_check
                     /note="To check sequence"
ORIGIN
        1 gcttcactgg ccgtcgtttt acaacgtcgt gactgggaaa accctggcgt
tacccaactt
       61 aatcgccttg cagcacatcc ccctttcgcc agctggcgta atagcgaaga
ggcccgcacc
      121 gatcgccctt cccaacagtt gcgcagcctg aatggcgaat ggacgcgccc
tgtagcggcg
      181 cattaagcgc ggcgggtgtg gtggttacgc gcagcgtgac cgctacactt
gccagcgccc
      241 tagcgcccgc tcctttcgct ttcttccctt cctttctcgc cacgttcgcc
ggctttcccc
      301 gtcaagctct aaatcggggg ctccctttag ggttccgatt tagtgcttta
cggcacctcg
      361 accccaaaaa acttgattag ggtgatggtt cacgtagtgg gccatcgccc
tgatagacgg
      421 tttttcgccc tttgacgttg gagtccacgt tctttaatag tggactcttg
ttccaaactg
      481 gaacaacact caaccctatc tcggtctatt cttttgattt ataagggatt
ttgccgattt
      541 cggcctattg gttaaaaaat gagctgattt aacaaaaatt taacgcgaat
tttaacaaaa
```

601 tattaacgct tacaatttcc tgatgcggta ttttctcctt acgcatctgt gcggtatttc
661 acaccgcata gggtaataac tgatataatt aaattgaagc tctaatttgt gagtttagta
721 ttaggatctc taccettggc gaaaagtcct ctgccaacaa tgatgatatc tgatccacca
781 cttacaactt cgtcgacggt tctgtactgc tgacccaata tgcgtatata taccaatcta
841 agtctgtgct ccttccttcg ttcttccttc tgttcggaga ttaccgaatc aaaaaaattt
901 caaggaaacc gaaatcaaaa aaaagaataa aaaaaaaatg atgaattgaa aaggtggtat
961 ggtgcactct cagtacaatc tgctctgatg ccgcatagtt aagccagccc cgacacccgc
1021 caacacccgc tgacgcgccc tgacgggctt gtctgctccc ggcatccgct tacagacaag
1081 ctgtgaccgt ctccgggagc tgcatgtgtc agaggttttc accgtcatca ccgaaacgcg
1141 cgagacgaaa gggcctcgtg atacgcctat ttttataggt taatgtcatg ataataatgg
1201 tttcttagac gtcggcgcgc cgaggacgtc aggtggcact tttcggggaa atgtgcgcgg
1261 aacccctatt tgtttatttt tctaaataca ttcaaatatg tatccgctca tgagacaata
1321 accctgataa atgcttcaat aatattgaaa aaggaagagt atgagtattc aacatttccg
1381 tgtcgccctt attccctttt ttgcggcatt ttgccttcct gtttttgctc acccagaaac
1441 gctggtgaaa gtaaaagatg ctgaagatca gttgggtgca cgagtgggtt acatcgaact
1501 ggatctcaac agcggtaaga tccttgagag ttttcgcccc gaagaacgtt ttccaatgat
1561 gagcactttt aaagttctgc tatgtggcgc ggtattatcc cgtattgacg ccgggcaaga
1621 gcaactcggt cgccgcatac actattctca gaatgacttg gttgagtact caccagtcac
1681 agaaaagcat cttacggatg gcatgacagt aagagaatta tgcagtgctg ccataaccat
1741 gagtgataac actgcggcca acttacttct gacaacgatc ggaggaccga aggagctaac
1801 cgcttttttg cacaacatgg gggatcatgt aactcgcctt gatcgttggg aaccggagct
1861 gaatgaagcc ataccaaacg acgagcgtga caccacgatg cctgtagcaa tggcaacaac
1921 gttgcgcaaa ctattaactg gcgaactact tactctagct tcccggcaac aattaataga
1981 ctggatggag gcggataaag ttgcaggacc acttctgcgc tcggcccttc cggctggctg
2041 gtttattgct gataaatctg gagccggtga gcgtgggtct cgcggtatca ttgcagcact
2101 ggggccagat ggtaagccct cccgtatcgt agttatctac acgacgggga gtcaggcaac
2161 tatggatgaa cgaaatagac agatcgctga gataggtgcc tcactgatta agcattggta

2221 actgtcagac caagtttact catatatact ttagattgat ttaaaacttc atttttaatt
2281 taaaaggatc taggtgaaga tcctttttga taatctcatg accaaaatcc cttaacgtga
2341 gttttcgttc cactgagcgt cagaccccgt agaaaagatc aaaggatctt cttgagatcc
2401 tttttttctg cgcgtaatct gctgcttgca aacaaaaaaa ccaccgctac cagcggtggt
2461 ttgtttgccg gatcaagagc taccaactct ttttccgaag gtaactggct tcagcagagc
2521 gcagatacca aatactgttc ttctagtgta gccgtagtta ggccaccact tcaagaactc
2581 tgtagcaccg cctacatacc tcgctctgct aatcctgtta ccagtggctg ctgccagtgg
2641 cgataagtcg tgtcttaccg ggttggactc aagacgatag ttaccggata aggcgcagcg
2701 gtcgggctga acggggggtt cgtgcacaca gcccagcttg gagcgaacga cctacaccga
2761 actgagatac ctacagcgtg agctatgaga aagcgccacg cttcccgaag ggagaaaggc
2821 ggacaggtat ccggtaagcg gcagggtcgg aacaggagag cgcacgaggg agcttccagg
2881 gggaaacgcc tggtatcttt atagtcctgt cgggtttcgc cacctctgac ttgagcgtcg
2941 atttttgtga tgctcgtcag gggggcggag cctatggaaa aacgccagca acgcggcctt
3001 tttacggttc ctggcctttt gctggccttt tgctcacatg ttctttcctg cgttatcccc
3061 tgattctgtg gataaccgta ttaccgcctt tgagtgagct gataccgctc gccgcagccg
3121 aacgaccgag cgcagcgagt cagtgagcga ggaagcggaa gagcgcccaa tacgcaaacc
3181 gcctctcccc gcgcgttggc cgattcatta atgcagctgg cacgacaggt ttcccgactg
3241 gaaagcgggc agtgagcgca acgcaattaa tgtgagttag ctcactcatt aggcacccca
3301 ggctttacac tttatgcttc cggctcctat gttgtgtgga attgtgagcg gataacaatt
3361 tcacacagga aacagctatg accatgatta cgccaagctc ggaattaacc ctcactaaag
3421 ggaacaaaag ctgggccggc catttaaatg tttaaacgct aaattcgagt gaaacacagg
3481 aagatcagaa aatcctcatt tcatccatat taacaataat ttcaaatgtt tatttgcatt
3541 atttgaaact aggcaagaca agcaacgaaa cgtttttgaa aattttgagt attttcaata
3601 aatttgtaga ggactcagat attgaaaaaa agctacagca attaatactt gataagaaga
3661 gtattgagaa gggcaacggt tcatcatctc atggatctgc acatgaacaa acaccagagt
3721 caaacgacgt tgaaattgag gctactgcgc caattgatga caatacagac gatgataaca
3781 aaccgaagtt atctgatgta gaaaaggatt aaagatgcta agagatagtg atgatatttc

3841 ataaataatg taattctata tatgttaatt accttttttg cgaggcatat
ttatggtgaa
3901 ggataagttt tgaccatcaa agaaggttaa tgtggctgtg gtttcagggt
ccataaagcg
3961 ctagcagtgc ttttaactaa gaattattag tcttttctgc ttatttttttc
atcatagttt
4021 agaacacttt atattaacga atagtttatg aatctattta ggtttaaaaa
ttgatacagt
4081 tttataagtt actttttcaa agactcgtgc tgtctattgc ataatgcact
ggaaggggaa
4141 aaaaaaggtg cacacgcgtg gctttttctt gaatttgcag tttgaaaaat
gctgccaaac
4201 cagatgtcaa cacagctaca acgttatatt gaattttcaa aaattcttac
ttttttttttg
4261 gatggacgca aagaagttta ataatcatat tacatggcat taccaccata
tacatatcca
4321 tatacatatc catatctaat cttacttata tgttgtggaa atgtaaagag
ccccattatc
4381 ttagcctaaa aaaaccttct ctttggaact ttcagtaata cgcttaactg
ctcattgcta
4441 tattgaagtg tggccgcggc ggaggagtgc ggcggaggag gagcggcgga
ggagtgcggc
4501 ggaggaggag cggcggagga gtgcggcgga ggagtctaga aattgcgtcc
tcgtcttcac
4561 cggtcgcgtt cctgaaacgc agatgtgcct aatgccgcac tgctccgaac
aataaagatt
4621 ctacaatact agcttttatg gttatgaaga ggaaaaattg gcagtaacct
ggccccacaa
4681 accttcaaat taacgaatca aattaacaac cataggatga taatgcgatt
agttttttag
4741 ccttatttct ggggtaatta atcagcgaag cgatgatttt tgatctatta
acagatatat
4801 aaatggaaaa gctgcataac cactttaact aatactttca acattttcag
tttgtattac
4861 ttcttattca aatgtcataa aagtatcaac aaaaaattgt taatatacct
ctatacttta
4921 acgtcaagga gaaaaaacta taagatccac gtggccacca tggcttctac
ggaacttagc
4981 ctaaaaagaa ccttaacgga tattttagaa gacgagttgt accatactaa
tccaggtcac
5041 agtcagttta cgagtcatta tcaaaactat catccaaatg ctagtattac
tccatataag
5101 ttggtgaata agaacaagga aaacaacact tttacgtgga atcattcatt
acaacaccag
5161 aatgaatcga gtgcagcttc gatacccccca caacaaacct accatttccc
gatattcaac
5221 aaatacgcgg atcctacttt aactaccacc acctctttta cgactagtga
agcaacggcc
5281 aacgatagac agattaataa tgtccatctc ataccaaacg agattaaggg
tgctagcgaa
5341 accccattgc agaagaccgt caatctaaag aatataatga aagtatcaga
cccgtatgta
5401 ccgacacgga atacgttcaa ttatgatgtt aaaatttcca acgatttttt
cgataacggt

5461 gacaatctat atggtaatga tgaagaagtg cttttctatg aggataatta taatccgaaa
5521 atgcagtggt cacttcaaga taatagcgcc gcaataaaca atgaggatgc gagagctatt
5581 tttaacaatg aatttgactc tgatgacgac gatatcagtg atgatgaaga ggatgaaata
5641 gaagaaaatt gtttgcaaca agagcaacac caagaggagc ctttactgtc attggatgtt
5701 acaccaatct caatgtttgg ctcagatcaa aaaacgggtc gtgccaagag ttctagtcat
5761 ttatttaatg agtacagtta cgttgactct aacatggaca gcatttccag tgttgtatct
5821 gaagatctgt tagatgaacg gggacatgag aagatagagg atgaggatga ggataatgat
5881 cttgatgaag acgatatcta cgatatctct ctcttgaaga acagaagaaa gcaaagtttt
5941 gtcctcaata aaaacactat tgattttgaa agatttccat ctccctcaac ctcggcaaac
6001 gtaccgtcta ctgctactac cggtaaaagg aaaccagcaa aatcatccag taaccgtagt
6061 tgcgttagta acagtaatga aaacggcaca ttagaaagaa taaagaagcc tacatcagct
6121 gtagtaagct caaatgctag taggcggaag ctaattaatt atactaagaa gcacttatct
6181 tcacattcat ctacaaattc gaattcgaaa ccttcgactg catcaccatc ggcccatacg
6241 tcatcttctg acggtaataa cgaaatattt acgtgtcaga taatgaatct cattacaaat
6301 gaaccgtgtg gtgcccaatt ttcaaggtcc tatgatttaa cgagacacca aaataccatt
6361 cacgctaaaa ggaagattgt cttccgttgc tcggagtgta taaaaattct tggatctgag
6421 ggctatcaga agacgttttc gagactggat gctttaacaa ggcatataaa atcgaagcat
6481 gaagatttgt cgttagaaca acgtcaagaa gttacaaaat ttgcaaaggc taatattggt
6541 tatgtcatgg gttaattaag gttacaaatt atataggaga taaaagaaga aaacctcaca
6601 caaaatggaa atacatatac acatctatat atatttacaa tatatatcat atatctgcat
6661 tcaggaatgt tcttataaat atcacatttt taaagtacct cattggactt ataataagtt
6721 ttctattggt tttcattata cttcggaaaa tacacaatta tattatatac ttacccccct
6781 taaggatttc ctaggcctac tgacctgcag gacgatggct tcttatctca cttcaatagt
6841 actttccacc ggttatactt ccggcttttc cctattaata caagctacaa tttcaatggg
6901 tggcaaataa tgtgtagaat agaaaataag ccgacagggt aataaagaaa atttttagaa
6961 aaaaaaggtt agatggctta tttaagttac aggctagcga aaaaaggaac ttcagggcaa
7021 gtaaagtgtt tgattgggca ctagcatggc ttataaaggc gagcaattgt cgaaactaat

7081 taatgttgta cggactattg ctgtcatctc gtggtaaatg cgtgttccag
gtcgaatact
7141 acttgcacac aggcgagcgg ggccccataa aagtgttgcc gatttgttaa
gttgtctttt
7201 cggtttttct actctgttat tccttacttc ccttttttaag aactcttttt
atccttcatt
7261 taggatcttg cacgtttccg cctcatcact tgaattaaaa catgtctctg
tcagtaaacc
7321 ttggcgtttc tattgttctt catagttcaa cttttattat tacccgccct
gcgcgtttac
7381 atttttccag caacagccag cgaaaaatta gaaaatctgg ttgttgacac
ctcaagaaca
7441 agggcaatta gcctcagcgt cgaatataga tcatattaga atacctatag
ctccatcaaa
7501 agaaatacac aagatctatg ggtacccgcc catatgcttg ccctgtcgag
tcctgcgatc
7561 gccgcttttc tcgccacgcc aatcttaccc gccatatccg catccatacc
ggtcagaagc
7621 ccttccagtg tcgaatctgc atgcgtaact tcagtcgtaa tgcgaacctt
gtgcgccaca
7681 tccgcaccca cacaggatcc caaaagccgt tccaatgtcg gatctgtatg
cggaacttta
7741 gtcgaaaggc cgacctgagg cgtcacattc gcacgcacac cggcgagaag
ccttttgcct
7801 gtgacatttg tgggaggaag tttgccagga agggcgacct caagaggcat
accaaaatcc
7861 atacaggtag atccggtgac ggtgctggtt taattaactc tgctggagac
atgagagctg
7921 ccaacctttg gccaagcccg ctcatgatca aacgctctaa gaagaacagc
ctggccttgt
7981 ccctgacggc cgaccagatg gtcagtgcct tgttggatgc tgagcccccc
atactctatt
8041 ccgagtatga tcctaccaga cccttcagtg aagcttcgat gatgggctta
ctgaccaacc
8101 tggcagacag ggagctggtt cacatgatca actgggcgaa gagggtgcca
ggctttgtgg
8161 atttgaccct ccatgatcag gtccaccttc tagaatgtgc ctggctagag
atcctgatga
8221 ttggactcgt ctggcgctcc atggagcacc cagggaagct actgtttgct
cctaacttgc
8281 tcttggacag gaaccaggga aaatgtgtag agggcatggt ggagatcttc
gacatgctgc
8341 tggctacatc atctcggttc cgcatgatga atctgcaggg agaggagttt
gtgtgcctca
8401 aatctattat tttgcttaat tctggagtgt acacatttct gtccagcacc
ctgaagtctc
8461 tggaagagaa ggaccatatc caccgagtcc tggacaagat cacagacact
ttgatccacc
8521 tgatggccaa ggcaggcctg accctgcagc agcagcacca gcggctggcc
cagctcctcc
8581 tcatcctctc ccacatcagg cacatgagta acaaaggcat ggagcatctg
tacagcatga
8641 agtgcaagaa cgtggtgccc ctctatgacc tgctgctgga gatgctggac
gcccaccgcc

8701 tacatgcgcc cactagccgt ggaggggcat ccgtggagga aacggaccaa agccacttgg
8761 ccactgcggg ctctacttca tcgcattcct tgcaaaagta ttacatcacg ggggaggcag
8821 agggtttccc tgccacagtc gcggctgcag gtgacggtgc tggtttaatt aacatgacgg
8881 tcgaccatga tttcaatagc gaagatattt tattccccat agaaagcatg agtagtatac
8941 aatacgtgga gaataataac ccaaataata ttaacaacga tgttatcccg tattctctag
9001 atatcaaaaa cactgtctta gatagtgcgg atctcaatga cattcaaaat caagaaactt
9061 cactgaattt ggggcttcct ccactatctt tcgactctcc actgcccgta acggaaacga
9121 taccatccac taccgataac agcttgcatt tgaaagctga tagcaacaaa aatcgcgatg
9181 caagaactat tgaaaatgat agtgaaatta agagtactaa taatgctagt ggctctgggg
9241 caaatcaata cacaactctt acttcacctt atcctatgaa cgacattttg tacaacatga
9301 acaatccgtt acaatcaccg tcaccttcat cggtacctca aaatccgact ataaatcctc
9361 ccataaatac agcaagtaac gaaactaatt tatcgcctca aacttcaaat ggtaatgaaa
9421 ctcttatatc tcctcgagcc caacaacata cgtccattaa agataatcgt ctgtccttac
9481 ctaatggtgc taattcgaat cttttcattg acactaaccc aaacaatttg aacgaaaaac
9541 taagaaatca attgaactca gatacaaatt catattctaa ctccatttct aattcaaact
9601 ccaattctac gggtaattta aattccagtt attttaattc actgaacata gactccatgc
9661 tagatgatta cgtttctagt gatctcttat tgaatgatga tgatgatgac actaatttat
9721 cacgccgaag atttagcgac gttataacaa accaatttcc gtcaatgaca aattcgagga
9781 atgagctcgg atcctaactt aagtctgaag aatgaatgat ttgatgattt ctttttccct
9841 ccatttttct tactgaatat atcaatgata tagacttgta tagtttatta tttcaaatta
9901 agtagctata tatagtcaag ataacgtttg tttgacacga ttacattatt cgtcgacatc
9961 ttttttcagc ctgtcgtggt agcaatttga ggagtattat taattgaata ggttcatttt
10021 gcgctcgcat aaacagtttt cgtcagggac agtatgttgg aatgagtggt aattaatggt
10081 gacatgacat gttatagcaa taaccttgat gtttacatcg tagtttaatg tacaccccgc
10141 gaattcgttc aagtaggagt gcaccaattg caaagggaaa agctgaatgg gcagttcgaa
10201 ccgcggtctt tcctgcgtta tcccctgatt ctgtggataa ccgtattacc gcctttgagt
10261 gagctgatac cgctcgccgc agccgaacga ccgagcgcag cgagtcagtg agcgaggctg

10321 agcgacatgg aggcccagaa taccctcctt gacagtcttg acgtgcgcag ctcaggggca
10381 tgatgtgact gtcgcccgta catttagccc atacatcccc atgtataatc atttgcatcc
10441 atacattttg atggccgcac ggcgcgaagc aaaaattacg gctcctcgct gcagacctgc
10501 gagcagggaa acgctcccct cacagacgcg ttgaattgtc cccacgccgc gcccctgtag
10561 agaaatataa aaggttagga tttgccactg aggttcttct ttcatatact tccttttaaa
10621 atcttgctag gatacagttc tcacatcaca tccgaacata aacaaccatg ggtaaggaaa
10681 agactcacgt ttcgaggccg cgattaaatt ccaacatgga tgctgattta tatgggtata
10741 aatgggctcg cgataatgtc gggcaatcag gtgcgacaat ctatcgattg tatgggaagc
10801 ccgatgcgcc agagttgttt ctgaaacatg gcaaaggtag cgttgccaat gatgttacag
10861 atgagatggt cagactaaac tggctgacgg aatttatgcc tcttccgacc atcaagcatt
10921 ttatccgtac tcctgatgat gcatggttac tcaccactgc gatccccggc aaaacagcat
10981 tccaggtatt agaagaatat cctgattcag gtgaaaatat tgttgatgcg ctggcagtgt
11041 tcctgcgccg gttgcattcg attcctgttt gtaattgtcc ttttaacagc gatcgcgtat
11101 ttcgtctcgc tcaggcgcaa tcacgaatga ataacggttt ggttgatgcg agtgattttg
11161 atgacgagcg taatggctgg cctgttgaac aagtctggaa agaaatgcat aagcttttgc
11221 cattctcacc ggattcagtc gtcactcatg gtgatttctc acttgataac cttatttttg
11281 acgaggggaa attaataggt tgtattgatg ttggacgagt cggaatcgca gaccgatacc
11341 aggatcttgc catcctatgg aactgcctcg gtgagttttc tccttcatta cagaaacggc
11401 tttttcaaaa atatggtatt gataatcctg atatgaataa attgcagttt catttgatgc
11461 tcgatgagtt tttctaatca gtactgacaa taaaaagatt cttgttttca agaacttgtc
11521 atttgtatag tttttttata ttgtagttgt tctattttaa tcaaatgtta gcgtgattta
11581 tattttttttt cgcctcgaca tcatctgccc agatgcgaag ttaagtgcgc agaaagtaat
11641 atcatgcgtc aatcgtatgt gaatgctggt cgctatactg gcggccgccc tatagtgagt
11701 cgtattagag ttattacccg agtagagcac ttgaatccac tgccccggga atctcggtcg
11761 taatgatttc tataatgacg aaaaaaaaaa aattggaaag aaaaagcttc atggccttta
11821 taaaaaggaa ctatccaata cctcgccaga accaagtaac agtattttac ggggcacaaa
11881 tcaagaacaa taagacagga ctgtaaagat ggacgcattg aactccaaag aacaacaaga

11941 gttccaaaaa gtagtggaac aaaagcaaat gaaggatttc atgcgtttgt actctaatct
12001 ggtagaaaga tgtttcacag actgtgtcaa tgacttcaca acatcaaagc taaccaataa
12061 ggaacaaaca tgcatcatga agtgctcaga aaagttcttg aagcatagcg aacgtgtagg
12121 gcagcgtttc caagaacaaa acgctgcctt gggacaaggc ttgggccgat aaggtgtact
12181 ggcgtatata tatctaatta tgtatctctg gtgtagccca tttttagcat gtaaatataa
12241 agaccgagcg ggccggcctc gggccatggt cactg
//

Plasmid: iRFP713_El_Samad for iRFP

```
LOCUS       iRFP713_El_Samad        2613 bp    DNA     circular SYN 26-
AUG-2021
DEFINITION  synthetic circular DNA
ACCESSION   .
VERSION     .
KEYWORDS    .
SOURCE      synthetic DNA construct
  ORGANISM  synthetic DNA construct
REFERENCE   1  (bases 1 to 2613)
  AUTHORS   Trial User
  TITLE     Direct Submission
  JOURNAL   Exported Dec 22, 2025 from SnapGene 8.2.1
            https://www.snapgene.com
COMMENT     Sequence Label: MTK3b_011 -iRFP713 -pAN307
FEATURES             Location/Qualifiers
     source          1..2613
                     /mol_type="other DNA"
                     /organism="synthetic DNA construct"
     misc_feature    complement(1274..1382)

     terminator      complement(1278..1312)
                     /label=lambda t0 terminator
                     /note="minimal transcription terminator from phage
lambda
                     (Scholtissek and Grosse, 1987)"
     CDS             complement(1383..2042)
                     /codon_start=1
                     /gene="cat"
                     /product="chloramphenicol acetyltransferase"
                     /label=CmR
                     /note="confers resistance to chloramphenicol"

/translation="MEKKITGYTTVDISQWHRKEHFEAFQSVAQCTYNQTVQLDITAFL

KTVKKNKHKFYPAFIHILARLMNAHPEFRMAMKDGELVIWDSVHPCYTVFHEQTETFSS

LWSEYHDDFRQFLHIYSQDVACYGENLAYFPKGFIENMFFVSANPWVSFTSFDLNVANM

DNFFAPVFTMGKYYTQGDKVLMPLAIQVHHAVCDGFHVGRMLNELQQYCDEWQGGA"
     misc_feature    complement(2043..2147)
                     /label=CamR Promoter
     misc_feature    complement(join(2148..2613,1..298))
                     /label=ColE1
ORIGIN
        1 gctatgagaa agcgccacgc ttcccgaagg gagaaaggcg gacaggtatc
cggtaagcgg
       61 cagggtcgga acaggagagc gcacgaggga gcttccaggg ggaaacgcct
ggtatcttta
      121 tagtcctgtc gggtttcgcc acctctgact tgagcgtcga tttttgtgat
gctcgtcagg
      181 ggggcggagc ctatggaaaa acgccagcaa cgcggccttt ttacggttcc
tggccttttg
```

241 ctggcctttt gctcacatgt tctttcctgc gttatcccct gattctgtgg ataaccgtag
301 tcggtctcat tctgctgaag gttctgttgc tagacaacca gacttgttga cttgtgacga
361 cgaaccaatc cacatcccag gtgctatcca accacacggt ttgttgttgg ctttggctgc
421 tgacatgact atcgttgctg gttctgacaa cttgccagaa ttgactggtt tggctatcgg
481 tgctttgatc ggtagatctg ctgctgacgt tttcgactct gaaactcaca acagattgac
541 tatcgctttg gctgaaccag gtgctgctgt tggtgctcca atcactgttg gtttcactat
601 gagaaaggac gctggtttca tcggttcttg gcacagacac gaccaattga tcttcttgga
661 attggaacca ccacaaagag atgttgctga accacaagct ttcttcagaa gaactaactc
721 tgctatcaga agattgcaag ctgctgaaac tttggaatct gcttgtgctg ctgctgctca
781 agaagttaga aagatcactg gtttcgacag agttatgatc tacagattcg cttctgactt
841 ctctggtgaa gttatcgctg aagacagatg tgctgaagtt gaatctaagt tgggtttgca
901 ctacccagct tctactgttc cagctcaagc tagaagattg tacactatca acccagttag
961 aatcatccca gacatcaact acagaccagt tccagttact ccagacttga acccagttac
1021 tggtagacca atcgacttgt ctttcgctat cttgagatct gtttctccag ttcacttgga
1081 attcatgaga aacatcggta tgcacggtac tatgtctatc tctatcttga gaggtgaaag
1141 attgtggggt ttgatcgttt gtcaccacag aactccatac tacgttgact tggacggtag
1201 acaagcttgt gaattggttg ctcaagtttt ggcttggcaa atcggtgtta tggaagaagg
1261 atcctgagac cagaccaata aaaaacgccc ggcggcaacc gagcgttctg aacaaatcca
1321 gatggagttc tgaggtcatt actggatcta tcaacaggag tccaagcgag ctcgatatca
1381 aattacgccc cgccctgcca ctcatcgcag tactgttgta attcattaag cattctgccg
1441 acatggaagc catcacaaac ggcatgatga acctgaatcg ccagcggcat cagcaccttg
1501 tcgccttgcg tataatattt gcccatggtg aaaacggggg cgaagaagtt gtccatattg
1561 gccacgttta aatcaaaact ggtgaaactc acccagggat tggctgaaac gaaaaacata
1621 ttctcaataa accctttagg gaaataggcc aggttttcac cgtaacacgc cacatcttgc
1681 gaatatatgt gtagaaactg ccggaaatcg tcgtggtatt cactccagag cgatgaaaac
1741 gtttcagttt gctcatggaa aacggtgtaa caagggtgaa cactatccca tatcaccagc
1801 tcaccgtctt tcattgccat acgaaattcc ggatgagcat tcatcaggcg ggcaagaatg

1861 tgaataaagg ccggataaaa cttgtgctta tttttcttta cggtctttaa aaaggccgta
1921 atatccagct gaacggtctg gttataggta cattgagcaa ctgactgaaa tgcctcaaaa
1981 tgttctttac gatgccattg ggatatatca acggtggtat atccagtgat ttttttctcc
2041 atttagctt ccttagctcc tgaaaatctc gataactcaa aaaatacgcc cggtagtgat
2101 cttatttcat tatggtgaaa gttggaacct cttacgtgcc cgatcaatca tgaccaaaat
2161 cccttaacgt gagttttcgt tccactgagc gtcagacccc gtagaaaaga tcaaaggatc
2221 ttcttgagat cctttttttc tgcgcgtaat ctgctgcttg caaacaaaaa aaccaccgct
2281 accagcggtg gtttgtttgc cggatcaaga gctaccaact ctttttccga aggtaactgg
2341 cttcagcaga gcgcagatac caaatactgt tcttctagtg tagccgtagt taggccacca
2401 cttcaagaac tctgtagcac cgcctacata cctcgctctg ctaatcctgt taccagtggc
2461 tgctgccagt ggcgataagt cgtgtcttac cgggttggac tcaagacgat agttaccgga
2521 taaggcgcag cggtcgggct gaacgggggg ttcgtgcaca cagcccagct tggagcgaac
2581 gacctacacc gaactgagat acctacagcg tga
//

Plasmid: KAN_Plasmid for knockout with KAN

```
LOCUS       KAN_Plasmid            4375 bp    DNA     circular SYN 19-
AUG-2020
DEFINITION  synthetic circular DNA
ACCESSION   .
VERSION     .
KEYWORDS    .
SOURCE      synthetic DNA construct
  ORGANISM  synthetic DNA construct
REFERENCE   1  (bases 1 to 4375)
  AUTHORS   Changhui Deng
  TITLE     Direct Submission
  JOURNAL   Exported Dec 22, 2025 from SnapGene 8.2.1
            https://www.snapgene.com
FEATURES             Location/Qualifiers
     source          1..4375
                     /mol_type="other DNA"
                     /organism="synthetic DNA construct"
     gene            115..1471
                     /label=kanMX
                     /note="yeast selectable marker conferring kanamycin
                     resistance (Wach et al., 1994)"
     promoter        115..458
                     /label=TEF promoter
                     /note="Ashbya gossypii TEF promoter"
     CDS             459..1268
                     /codon_start=1
                     /gene="aph(3')-Ia"
                     /product="aminoglycoside phosphotransferase"
                     /label=KanR
                     /note="confers resistance to kanamycin"

/translation="MGKEKTHVSRPRLNSNMDADLYGYKWARDNVGQSGATIYRLYGKP

DAPELFLKHGKGSVANDVTDEMVRLNWLTEFMPLPTIKHFIRTPDDAWLLTTAIPGKTA

FQVLEEYPDSGENIVDALAVFLRRLHSIPVCNCPFNSDRVFRLAQAQSRMNNGLVDASD

FDDERNGWPVEQVWKEMHKLLPFSPDSVVTHGDFSLDNLIFDEGKLIGCIDVGRVGIAD
                     RYQDLAILWNCLGEFSPSLQKRLFQKYGIDNPDMNKLQFHLMLDEFF"
     terminator      1274..1471
                     /label=TEF terminator
                     /note="Ashbya gossypii TEF terminator"
     promoter        1518..1916
                     /gene="S. cerevisiae TEF1"
                     /label=TEF1 promoter
                     /note="promoter for EF-1-alpha"
     primer_bind     1920..1936
                     /label=SK primer
                     /note="common sequencing primer, one of multiple
similar
                     variants"
     promoter        complement(2013..2031)
```

```
                     /label=T7 promoter
                     /note="promoter for bacteriophage T7 RNA polymerase"
     rep_origin      complement(2289..2877)
                     /direction=LEFT
                     /label=ori
                     /note="high-copy-number ColE1/pMB1/pBR322/pUC origin
of
                     replication"
     CDS             complement(join(3048..3839,3840..3908))
                     /codon_start=1
                     /gene="bla"
                     /product="beta-lactamase"
                     /label=AmpR
                     /note="confers resistance to ampicillin,
carbenicillin, and
                     related antibiotics"

/translation="MSIQHFRVALIPFFAAFCLPVFAHPETLVKVKDAEDQLGARVGYI

ELDLNSGKILESFRPEERFPMMSTFKVLLCGAVLSRIDAGQEQLGRRIHYSQNDLVEYS

PVTEKHLTDGMTVRELCSAAITMSDNTAANLLLTTIGGPKELTAFLHNMGDHVTRLDRW

EPELNEAIPNDERDTTMPVAMATTLRKLLTGELLTLASRQQLIDWMEADKVAGPLLRSA

LPAGWFIADKSGAGERGSRGIIAALGPDGKPSRIVVIYTTGSQATMDERNRQIAEIGAS
                     LIKHW"
     promoter        complement(3909..4013)
                     /gene="bla"
                     /label=AmpR promoter
ORIGIN
        1 gaacgcggcc gccagctgaa gcttcgtacg ctgcaggtcg acggatcccc
gggttaatta
       61 aggcgcgcca gatctgttta gcttgcctcg tccccgccgg gtcacccggc
cagcgacatg
      121 gaggcccaga ataccctcct tgacagtctt gacgtgcgca gctcaggggc
atgatgtgac
      181 tgtcgcccgt acatttagcc catacatccc catgtataat catttgcatc
catacatttt
      241 gatggccgca cggcgcgaag caaaaattac ggctcctcgc tgcagacctg
cgagcaggga
      301 aacgctcccc tcacagacgc gttgaattgt ccccacgccg cgcccctgta
gagaaatata
      361 aaaggttagg atttgccact gaggttcttc tttcatatac ttccttttaa
aatcttgcta
      421 ggatacagtt ctcacatcac atccgaacat aaacaaccat gggtaaggaa
aagactcacg
      481 tttcgaggcc gcgattaaat tccaacatgg atgctgattt atatgggtat
aaatgggctc
      541 gcgataatgt cgggcaatca ggtgcgacaa tctatcgatt gtatgggaag
cccgatgcgc
      601 cagagttgtt tctgaaacat ggcaaaggta gcgttgccaa tgatgttaca
gatgagatgg
```

661 tcagactaaa ctggctgacg gaatttatgc ctcttccgac catcaagcat tttatccgta
721 ctcctgatga tgcatggtta ctcaccactg cgatccccgg caaaacagca ttccaggtat
781 tagaagaata tcctgattca ggtgaaaata ttgttgatgc gctggcagtg ttcctgcgcc
841 ggttgcattc gattcctgtt tgtaattgtc cttttaacag cgatcgcgta tttcgtctcg
901 ctcaggcgca atcacgaatg aataacggtt tggttgatgc gagtgatttt gatgacgagc
961 gtaatggctg gcctgttgaa caagtctgga aagaaatgca taagcttttg ccattctcac
1021 cggattcagt cgtcactcat ggtgatttct cacttgataa ccttattttt gacgagggga
1081 aattaatagg ttgtattgat gttggacgag tcggaatcgc agaccgatac caggatcttg
1141 ccatcctatg gaactgcctc ggtgagtttt ctccttcatt acagaaacgg ctttttcaaa
1201 aatatggtat tgataatcct gatatgaata aattgcagtt tcatttgatg ctcgatgagt
1261 ttttctaatc agtactgaca ataaaaagat tcttgttttc aagaacttgt catttgtata
1321 gtttttttat attgtagttg ttctatttta atcaaatgtt agcgtgattt atattttttt
1381 tcgcctcgac atcatctgcc cagatgcgaa gttaagtgcg cagaaagtaa tatcatgcgt
1441 caatcgtatg tgaatgctgg tcgctatact gctgtcgatt cgatactaac gccgccatcc
1501 agtgtcgaaa acgagctcat agcttcaaaa tgtttctact cctttttac tcttccagat
1561 tttctcggac tccgcgcatc gccgtaccac ttcaaaacac ccaagcacag catactaaat
1621 ttcccctctt tcttcctcta gggtgtcgtt aattacccgt actaaaggtt tggaaaagaa
1681 aaaagagacc gcctcgtttc tttttcttcg tcgaaaaagg caataaaaat ttttatcacg
1741 tttcttttc ttgaaaattt ttttttgat tttttctct ttcgatgacc tcccattgat
1801 atttaagtta ataaacggtc ttcaatttct caagtttcag tttcattttt cttgttctat
1861 tacaactttt tttacttctt gctcattaga aagaaagcat agcaatctaa tctaagtttt
1921 ctagaactag tggatccccc ccggacgaca gagaattcat cgatgatatc agatccacta
1981 gtggcctatg cggccgcgga tctgccggtc tccctatagt gagtcgtatt aatttcgata
2041 agccaggtta acctgcatta atgaatcggc caacgcgcgg ggagaggcgg tttgcgtatt
2101 gggcgctctt ccgcttcctc gctcactgac tcgctgcgct cggtcgttcg gctgcggcga
2161 gcggtatcag ctcactcaaa ggcggtaata cggttatcca cagaatcagg ggataacgca
2221 ggaaagaaca tgtgagcaaa aggccagcaa aaggccagga accgtaaaaa ggccgcgttg

2281 ctggcgtttt tccataggct ccgcccccct gacgagcatc acaaaaatcg acgctcaagt
2341 cagaggtggc gaaacccgac aggactataa agataccagg cgtttccccc tggaagctcc
2401 ctcgtgcgct ctcctgttcc gaccctgccg cttaccggat acctgtccgc ctttctccct
2461 tcgggaagcg tggcgctttc tcaatgctca cgctgtaggt atctcagttc ggtgtaggtc
2521 gttcgctcca agctgggctg tgtgcacgaa ccccccgttc agcccgaccg ctgcgcctta
2581 tccggtaact atcgtcttga gtccaacccg gtaagacacg acttatcgcc actggcagca
2641 gccactggta acaggattag cagagcgagg tatgtaggcg gtgctacaga gttcttgaag
2701 tggtggccta actacggcta cactagaagg acagtatttg gtatctgcgc tctgctgaag
2761 ccagttacct tcggaaaaag agttggtagc tcttgatccg gcaaacaaac caccgctggt
2821 agcggtggtt tttttgtttg caagcagcag attacgcgca gaaaaaaagg atctcaagaa
2881 gatcctttga tcttttctac ggggtctgac gctcagtgga acgaaaactc acgttaaggg
2941 attttggtca tgagattatc aaaaaggatc ttcacctaga tccttttaaa ttaaaaatga
3001 agttttaaat caatctaaag tatatatgag taaacttggt ctgacagtta ccaatgctta
3061 atcagtgagg cacctatctc agcgatctgt ctatttcgtt catccatagt tgcctgactc
3121 cccgtcgtgt agataactac gatacgggag ggcttaccat ctggccccag tgctgcaatg
3181 ataccgcgag acccacgctc accggctcca gatttatcag caataaacca gccagccgga
3241 agggccgagc gcagaagtgg tcctgcaact ttatccgcct ccatccagtc tattaattgt
3301 tgccgggaag ctagagtaag tagttcgcca gttaatagtt tgcgcaacgt tgttgccatt
3361 gctacaggca tcgtggtgtc acgctcgtcg tttggtatgg cttcattcag ctccggttcc
3421 caacgatcaa ggcgagttac atgatccccc atgttgtgca aaaaagcggt tagctccttc
3481 ggtcctccga tcgttgtcag aagtaagttg gccgcagtgt tatcactcat ggttatggca
3541 gcactgcata attctcttac tgtcatgcca tccgtaagat gcttttctgt gactggtgag
3601 tactcaacca agtcattctg agaatagtgt atgcggcgac cgagttgctc ttgcccggcg
3661 tcaatacggg ataataccgc gccacatagc agaactttaa aagtgctcat cattggaaaa
3721 cgttcttcgg ggcgaaaact ctcaaggatc ttaccgctgt tgagatccag ttcgatgtaa
3781 cccactcgtg cacccaactg atcttcagca tcttttactt tcaccagcgt ttctgggtga
3841 gcaaaaacag gaaggcaaaa tgccgcaaaa aagggaataa gggcgacacg gaaatgttga

3901 atactcatac tcttcctttt tcaatattat tgaagcattt atcagggtta ttgtctcatg
3961 agcggataca tatttgaatg tatttagaaa aataaacaaa taggggttcc gcgcacattt
4021 ccccgaaaag tgccacctga cgtctaagaa accattatta tcatgacatt aacctataaa
4081 aataggcgta tcacgaggcc ctttcgtctc gcgcgtttcg gtgatgacgg tgaaaacctc
4141 tgacacatgc agctcccgga gacggtcaca gcttgtctgt aagcggatgc cgggagcaga
4201 caagcccgtc agggcgcgtc agcgggtgtt ggcgggtgtc ggggctggct taactatgcg
4261 gcatcagagc agattgtact gagagtgcac catatggaca tattgtcgtt agaacgcggc
4321 tacaattaat acataacctt atgtatcata cacatacgat ttaggtgaca ctata
//

Plasmid: Mcherry_psc211 for dtomatoh tag

```
LOCUS       Mcherry_psc211          6675 bp    DNA     circular SYN 11-
JUN-2022
DEFINITION  synthetic circular DNA
ACCESSION   Not-Specified.
VERSION     .
KEYWORDS    .
SOURCE      synthetic DNA construct
  ORGANISM  synthetic DNA construct
REFERENCE   1  (bases 1 to 6675)
  AUTHORS   .
  TITLE     Direct Submission
  JOURNAL   Exported Dec 22, 2025 from SnapGene 8.2.1
            https://www.snapgene.com
FEATURES             Location/Qualifiers
     source          1..6675
                     /organism="synthetic DNA construct"
     misc_feature    1..589
                     /gene="pCMV"
                     /label=pCMV
     misc_feature    554..560
                     /label=TATA Box
     primer_bind     586..612
                     /label=Forward
     primer_bind     597..636
                     /label=mCherry-S
     CDS             613..1320
                     /codon_start=1
                     /gene="mCherry"
                     /label=mCherry

/translation="MVSKGEEDNMAIIKEFMRFKVHMEGSVNGHEFEIEGEGEGRPYEG

TQTAKLKVTKGGPLPFAWDILSPQFMYGSKAYVKHPADIPDYLKLSFPEGFKWERVMNF

EDGGVVTVTQDSSLQDGEFIYKVKLRGTNFPSDGPVMQKKTMGWEASSERMYPEDGALK

GEIKQRLKLKDGGHYDAEVKTTYKAKKPVQLPGAYNVNIKLDITSHNEDYTIVEQYERA
                     EGRHSTGGMDELYK"
     primer_bind     complement(1300..1338)
                     /label=mCherry-A
     primer_bind     1336..1359
                     /label=FT-S
     CDS             1372..1461
                     /codon_start=1
                     /gene="3F6H"
                     /label=3F6H
                     /translation="DYKDHDGDYKDHDIDYKDDDDKHHHHHHG"
     primer_bind     1443..1485
                     /label=tADH1-S
     terminator      1470..1672
                     /gene="tADH1"
     primer_bind     complement(1657..1676)
```

```
                     /label=SBFI_reverse
     primer_bind     complement(1676..1727)
                     /label=tADH1-A
     misc_feature    1722..1781
                     /gene="loxP"
                     /label=loxP
     CDS             complement(1905..2708)
                     /gene="URA3"
                     /label=URA3
     misc_feature    3208..3261
                     /gene="loxP"
                     /label=loxP
     primer_bind     complement(3306..3329)
                     /label=RT-A
     misc_feature    3494..3544
                     /gene="SV40"
                     /label=SV40 pA
     misc_feature    3591..4046
                     /gene="f1 ori"
                     /label=f1 ori
     misc_feature    4220..4522
                     /gene="SV40 ori"
                     /label=SV40 ori
     CDS             4571..5365
                     /gene="Kan r/Neo r"
                     /label=KanR/NeoR
     misc_feature    5601..5619
                     /gene="HSV TK pA"
                     /label=HSV TK pA
     misc_feature    5950..6593
                     /gene="pUC"
                     /label=pUC
ORIGIN
        1 tagttattaa tagtaatcaa ttacggggtc attagttcat agcccatata
tggagttccg
       61 cgttacataa cttacggtaa atggcccgcc tggctgaccg cccaacgacc
cccgcccatt
      121 gacgtcaata atgacgtatg ttcccatagt aacgccaata gggactttcc
attgacgtca
      181 atgggtggag tatttacggt aaactgccca cttggcagta catcaagtgt
atcatatgcc
      241 aagtacgccc cctattgacg tcaatgacgg taaatggccc gcctggcatt
atgcccagta
      301 catgacctta tgggactttc ctacttggca gtacatctac gtattagtca
tcgctattac
      361 catggtgatg cggttttggc agtacatcaa tgggcgtgga tagcggtttg
actcacgggg
      421 atttccaagt ctccacccca ttgacgtcaa tgggagtttg ttttggcacc
aaaatcaacg
      481 ggactttcca aaatgtcgta acaactccgc cccattgacg caaatgggcg
gtaggcgtgt
      541 acggtgggag gtctatataa gcagagctgg tttagtgaac cgtcagatcc
gctagcgcta
```

601 ccggtcgcca ccatggtgag caagggcgag gaggataaca tggccatcat caaggagttc
661 atgcgcttca aggtgcacat ggagggctcc gtgaacggcc acgagttcga gatcgagggc
721 gagggcgagg gccgccccta cgagggcacc cagaccgcca agctgaaggt gaccaagggt
781 ggccccctgc ccttcgcctg ggacatcctg tcccctcagt tcatgtacgg ctccaaggcc
841 tacgtgaagc accccgccga catccccgac tacttgaagc tgtccttccc cgagggcttc
901 aagtgggagc gcgtgatgaa cttcgaggac ggcggcgtgg tgaccgtgac ccaggactcc
961 tccctgcagg acggcgagtt catctacaag gtgaagctgc gcggcaccaa cttcccctcc
1021 gacggccccg taatgcagaa gaagaccatg ggctgggagg cctcctccga gcggatgtac
1081 cccgaggacg gcgccctgaa gggcgagatc aagcagaggc tgaagctgaa ggacggcggc
1141 cactacgacg ctgaggtcaa gaccacctac aaggccaaga agcccgtgca gctgcccggc
1201 gcctacaacg tcaacatcaa gttggacatc acctcccaca acgaggacta caccatcgtg
1261 gaacagtacg aacgcgccga gggccgccac tccaccggcg gcatggacga gctgtacaag
1321 tccggactca gatcttacga tttaggtgac actatagaac gcggccgcca ggactacaag
1381 gaccatgacg gtgattacaa ggatcatgac atcgactaca aggatgacga tgacaagcat
1441 catcaccatc accatggttg aggcgcgcca cttctaaata agcgaatttc ttatgattta
1501 tgatttttat tattaaataa gttataaaaa aaataagtgt atacaaattt taaagtgact
1561 cttaggtttt aaaacgaaaa ttcttattct tgagtaactc tttcctgtag gtcaggttgc
1621 tttctcaggt atagtatgag gtcgctctta ttgaccacac ctctaccggc agatccgcta
1681 gggataacag ggtaatatat aagcttcgta cgtgactgca ggtcgacaac ccttaatata
1741 acttcgtata atgtatgcta tacgaagtta ttaggtctag agatcccaat acaacagatc
1801 acgtgatctt tttgtaagat gaagttgaag tagtgttgca ccgtgccaat gcaggtggct
1861 attagattaa atatgtgatt tgttctatta agtttcctgt ataattaatg gggagcgctg
1921 attctctttt ggtacgcttc ccatccagca tttctgtatc tttcaccttc aaccttagga
1981 tctctaccct tggcgaaaag tcctctgcca acaatgatga tatctgatcc accacttaca
2041 acttcgtcga cggttctgta ctgctgaccc aatgcatcgc ctttgtcgtc taaacctaca
2101 cctggggtca tgattagcca atcaaaccct tcttctcttc ctcccatatc gttctgagca
2161 atgaacccaa taacgaaatc tttatcactc tttgcaatat caacggtacc cttagtatat

2221 tcaccgtgtg ctagagaacc cttggaagac aattcagcaa gcatcaataa tccccttggt
2281 tctttggtga cctcttgcgc accttgtttc aagccagcaa caataccagc accagtaacc
2341 ccgtgggcgt tggtgatatc agaccattct gcgatacggt aaacgcccga tgtatattgt
2401 aatttgactg tgttaccgat atcggcgaat ttctgtcct caaatatcaa gaacttgtat
2461 ttctctgcca atgctttcaa tggaacgaca gtaccctcat aactgaaatc atccaagata
2521 tcaacgtgtg ttttcaaaag gcaaatgtat ggacccaacg tttcaacaag tttcaatagc
2581 tcatcagtcg aacgaacgtc aagagaagca cacaaattgg tcttcttttc atccattaaa
2641 cgtaaaagtt tcgatgcaac cggacttgca tgagtctcag ctctactggt atatgatttt
2701 gtggacatgg tgcaactaat tgacgggagt gtattgacgc tggcgtactg gctttcacaa
2761 aatggcccaa tcacaaccac atcttagata gttgaaatga ctttagataa catcaattga
2821 gatgagctta atcatgtcaa agctaaaagt gtcaccatga acgacaattc ttaagcaaat
2881 cacgtgatat agatccacga ataaccacca tttgatgctc gaggcaagta atgtgtgtaa
2941 aaaaatgcgt taccaccatc caatgcagac cgatcttcta cccagaatca catatattta
3001 tgtaccgagt accttttttc tatcttccaa ttgcttctcc catatgattg tctccgtaag
3061 ctcgaaattt ctaagttgga ttttaatctt cacgcaggat gacagttcga tgagcttctg
3121 aggagtgttt agaacataat cagtttatcc atggtctatc tcttcttgtc gctttttctc
3181 ctcgatagaa cctaaataaa acgagctctc gagaaccctt aatataactt cgtataatgt
3241 atgctatacg aagttattag gtgatatcag atccactagt ggcctatgcg gccgcggatc
3301 tgccggtctc cctatagtga gtcgtattac ccgggatcca ccggatctag ataactgatc
3361 ataatcagcc ataccacatt tgtagaggtt ttacttgctt taaaaaacct cccacacctc
3421 cccctgaacc tgaaacataa aatgaatgca attgttgttg ttaacttgtt tattgcagct
3481 tataatggtt acaaataaag caatagcatc acaaatttca caaataaagc atttttttca
3541 ctgcattcta gttgtggttt gtccaaactc atcaatgtat cttaaggcgt aaattgtaag
3601 cgttaatatt ttgttaaaat tcgcgttaaa tttttgttaa atcagctcat tttttaacca
3661 ataggccgaa atcggcaaaa tcccttataa atcaaaagaa tagaccgaga tagggttgag
3721 tgttgttcca gtttggaaca agagtccact attaaagaac gtggactcca acgtcaaagg
3781 gcgaaaaacc gtctatcagg gcgatggccc actacgtgaa ccatcaccct aatcaagttt

3841 tttggggtcg aggtgccgta aagcactaaa tcggaaccct aaagggagcc cccgatttag
3901 agcttgacgg ggaaagccgg cgaacgtggc gagaaaggaa gggaagaaag cgaaaggagc
3961 gggcgctagg gcgctggcaa gtgtagcggt cacgctgcgc gtaaccacca cacccgccgc
4021 gcttaatgcg ccgctacagg gcgcgtcagg tggcactttt cggggaaatg tgcgcggaac
4081 ccctatttgt ttatttttct aaatacattc aaatatgtat ccgctcatga gacaataacc
4141 ctgataaatg cttcaataat attgaaaaag gaagagtcct gaggcggaaa gaaccagctg
4201 tggaatgtgt gtcagttagg gtgtggaaag tccccaggct ccccagcagg cagaagtatg
4261 caaagcatgc atctcaatta gtcagcaacc aggtgtggaa agtccccagg ctccccagca
4321 ggcagaagta tgcaaagcat gcatctcaat tagtcagcaa ccatagtccc gcccctaact
4381 ccgcccatcc cgcccctaac tccgcccagt tccgcccatt ctccgcccca tggctgacta
4441 atttttttta tttatgcaga ggccgaggcc gcctcggcct ctgagctatt ccagaagtag
4501 tgaggaggct tttttggagg cctaggcttt tgcaaagatc gatcaagaga caggatgagg
4561 atcgtttcgc atgattgaac aagatggatt gcacgcaggt tctccggccg cttgggtgga
4621 gaggctattc ggctatgact gggcacaaca gacaatcggc tgctctgatg ccgccgtgtt
4681 ccggctgtca gcgcaggggc gcccggttct ttttgtcaag accgacctgt ccggtgccct
4741 gaatgaactg caagacgagg cagcgcggct atcgtggctg gccacgacgg gcgttccttg
4801 cgcagctgtg ctcgacgttg tcactgaagc gggaagggac tggctgctat tgggcgaagt
4861 gccggggcag gatctcctgt catctcacct tgctcctgcc gagaaagtat ccatcatggc
4921 tgatgcaatg cggcggctgc atacgcttga tccggctacc tgcccattcg accaccaagc
4981 gaaacatcgc atcgagcgag cacgtactcg gatggaagcc ggtcttgtcg atcaggatga
5041 tctggacgaa gagcatcagg ggctcgcgcc agccgaactg ttcgccaggc tcaaggcgag
5101 catgcccgac ggcgaggatc tcgtcgtgac ccatggcgat gcctgcttgc cgaatatcat
5161 ggtggaaaat ggccgctttt ctggattcat cgactgtggc cggctgggtg tggcggaccg
5221 ctatcaggac atagcgttgg ctacccgtga tattgctgaa gagcttggcg gcgaatgggc
5281 tgaccgcttc ctcgtgcttt acggtatcgc cgctcccgat tcgcagcgca tcgccttcta
5341 tcgccttctt gacgagttct tctgagcggg actctggggt tcgaaatgac cgaccaagcg
5401 acgcccaacc tgccatcacg agatttcgat tccaccgccg ccttctatga aaggttgggc

5461 ttcggaatcg ttttccggga cgccggctgg atgatcctcc agcgcgggga tctcatgctg
5521 gagttcttcg cccaccctag ggggaggcta actgaaacac ggaaggagac aataccggaa
5581 ggaacccgcg ctatgacggc aataaaaaga cagaataaaa cgcacggtgt tgggtcgttt
5641 gttcataaac gcggggttcg gtcccagggc tggcactctg tcgatacccc accgagaccc
5701 cattggggcc aatacgcccg cgtttcttcc ttttccccac cccacccccc aagttcgggt
5761 gaaggcccag ggctcgcagc caacgtcggg gcggcaggcc ctgccatagc ctcaggttac
5821 tcatatatac tttagattga tttaaaactt catttttaat ttaaaaggat ctaggtgaag
5881 atcctttttg ataatctcat gaccaaaatc ccttaacgtg agttttcgtt ccactgagcg
5941 tcagaccccg tagaaaagat caaaggatct tcttgagatc ctttttttct gcgcgtaatc
6001 tgctgcttgc aaacaaaaaa accaccgcta ccagcggtgg tttgtttgcc ggatcaagag
6061 ctaccaactc tttttccgaa ggtaactggc ttcagcagag cgcagatacc aaatactgtc
6121 cttctagtgt agccgtagtt aggccaccac ttcaagaact ctgtagcacc gcctacatac
6181 ctcgctctgc taatcctgtt accagtggct gctgccagtg gcgataagtc gtgtcttacc
6241 gggttggact caagacgata gttaccggat aaggcgcagc ggtcgggctg aacggggggt
6301 tcgtgcacac agcccagctt ggagcgaacg acctacaccg aactgagata cctacagcgt
6361 gagctatgag aaagcgccac gcttcccgaa gggagaaagg cggacaggta tccggtaagc
6421 ggcagggtcg gaacaggaga gcgcacgagg gagcttccag ggggaaacgc ctggtatctt
6481 tatagtcctg tcgggtttcg ccacctctga cttgagcgtc gatttttgtg atgctcgtca
6541 gggggcggga gcctatggaa aaacgccagc aacgcggcct ttttacggtt cctggccttt
6601 tgctggcctt ttgctcacat gttctttcct gcgttatccc ctgattctgt ggataaccgt
6661 attaccgcca tgcat
//

Plasmid: pSC113_NAT for GFP tagging with NAT

```
LOCUS       pSC113_NAT              6515 bp    DNA     circular SYN 04-
FEB-2020
DEFINITION  synthetic circular DNA
ACCESSION   .
VERSION     .
KEYWORDS    .
SOURCE      synthetic DNA construct
  ORGANISM  synthetic DNA construct
REFERENCE   1  (bases 1 to 6515)
  AUTHORS   .
  TITLE     Direct Submission
  JOURNAL   Exported Dec 22, 2025 from SnapGene 8.2.1
            https://www.snapgene.com
FEATURES             Location/Qualifiers
     source          1..6515
                     /mol_type="other DNA"
                     /organism="synthetic DNA construct"
     enhancer        61..364
                     /label=CMV enhancer
                     /note="human cytomegalovirus immediate early
enhancer"
     promoter        365..568
                     /label=CMV promoter
                     /note="human cytomegalovirus (CMV) immediate early
                     promoter"
     misc_feature    586..612
                     /label=Primer and Link
     primer_bind     586..606
                     /label=Forward
     regulatory      607..616
                     /label=Kozak sequence
                     /note="vertebrate consensus sequence for strong
initiation
                     of translation (Kozak, 1987)"
                     /regulatory_class="other"
     CDS             613..1329
                     /codon_start=1
                     /product="Aequoria victoria green fluorescent
protein"
                     /label=GFP

/translation="MVSKGEELFTGVVPILVELDGDVNGHKFSVSGEGEGDATYGKLTL

KFICTTGKLPVPWPTLVTTFTYGVQCFSRYPDHMKRHDFFKSAMPEGYVQERTIFFKDD

GNYKTRAEVKFEGDTLVNRIELKGIDFKEDGNILGHKLEYNYNSHNVYIMADKQKNGIK

VNFKIRHNIEDGSVQLADHYQQNTPIGDGPVLLPDNHYLSTQSALSKDPNEKRDHMVLL
                     EFVTAAGITHGMDELYK"
     promoter        1349..1367
                     /label=SP6 promoter
```

```
                     /note="promoter for bacteriophage SP6 RNA
polymerase"
     CDS             1381..1446
                     /codon_start=1
                     /product="three tandem FLAG(R) epitope tags,
followed by an
                     enterokinase cleavage site"
                     /label=3xFLAG
                     /translation="DYKDHDGDYKDHDIDYKDDDDK"
     CDS             1447..1464
                     /codon_start=1
                     /product="6xHis affinity tag"
                     /label=6xHis
                     /translation="HHHHHH"
     misc_feature    1479..1691
                     /label=KlLeu
                     /note="Portion is different from the URA3 plasmid"
     CDS             complement(1912..2484)
                     /codon_start=1
                     /gene="Streptomyces noursei nat1"
                     /product="nourseothricin acetyltransferase"
                     /label=NrsR
                     /note="confers resistance to nourseothricin"

/translation="MGTTLDDTAYRYRTSVPGDAEAIEALDGSFTTDTVFRVTATGDGF

TLREVPVDPPLTKVFPDDESDDESDDGEDGDPDSRTFVAYGDDGDLAGFVVVSYSGWNR

RLTVEDIEVAPEHRGHGVGRALMGLATEFARERGAGHLWLEVTNVNAPAIHAYRRMGFT
                     LCGLDTALYDGTASDGEQALYMSMPCP"
     primer_bind     complement(3146..3169)
                     /label=Reverse
     promoter        complement(3151..3169)
                     /label=T7 promoter
                     /note="promoter for bacteriophage T7 RNA polymerase"
     polyA_signal    3303..3424
                     /label=SV40 poly(A) signal
                     /note="SV40 polyadenylation signal"
     rep_origin      complement(3431..3886)
                     /direction=LEFT
                     /label=f1 ori
                     /note="f1 bacteriophage origin of replication; arrow
                     indicates direction of (+) strand synthesis"
     promoter        3913..4017
                     /gene="bla"
                     /label=AmpR promoter
     promoter        4019..4376
                     /label=SV40 promoter
                     /note="SV40 enhancer and early promoter"
     rep_origin      4227..4362
                     /label=SV40 ori
                     /note="SV40 origin of replication"
     CDS             4411..5205
                     /codon_start=1
```

/gene="aph(3')-II (or nptII)"
/product="aminoglycoside phosphotransferase from Tn5"
/label=NeoR/KanR
/note="confers resistance to neomycin, kanamycin, and G418
(Geneticin(R))"

/translation="MIEQDGLHAGSPAAWVERLFGYDWAQQTIGCSDAAVFRLSAQGRP

VLFVKTDLSGALNELQDEAARLSWLATTGVPCAAVLDVVTEAGRDWLLLGEVPGQDLLS

SHLAPAEKVSIMADAMRRLHTLDPATCPFDHQAKHRIERARTRMEAGLVDQDDLDEEHQ

GLAPAELFARLKASMPDGEDLVVTHGDACLPNIMVENGRFSGFIDCGRLGVADRYQDIA
LATRDIAEELGGEWADRFLVLYGIAAPDSQRIAFYRLLDEFF"

polyA_signal 5437..5484
/label=HSV TK poly(A) signal
/note="herpes simplex virus thymidine kinase
polyadenylation signal (Cole and Stacy, 1985)"

rep_origin 5813..6401
/direction=RIGHT
/label=ori
/note="high-copy-number ColE1/pMB1/pBR322/pUC origin of
replication"

ORIGIN

```
        1 tagttattaa tagtaatcaa ttacggggtc attagttcat agcccatata tggagttccg
       61 cgttacataa cttacggtaa atggcccgcc tggctgaccg cccaacgacc cccgcccatt
      121 gacgtcaata atgacgtatg ttcccatagt aacgccaata gggactttcc attgacgtca
      181 atgggtggag tatttacggt aaactgccca cttggcagta catcaagtgt atcatatgcc
      241 aagtacgccc cctattgacg tcaatgacgg taaatggccc gcctggcatt atgcccagta
      301 catgacctta tgggactttc ctacttggca gtacatctac gtattagtca tcgctattac
      361 catggtgatg cggttttggc agtacatcaa tgggcgtgga tagcggtttg actcacgggg
      421 atttccaagt ctccacccca ttgacgtcaa tgggagtttg ttttggcacc aaaatcaacg
      481 ggactttcca aaatgtcgta acaactccgc cccattgacg caaatgggcg gtaggcgtgt
      541 acggtgggag gtctatataa gcagagctgg tttagtgaac cgtcagatcc gctagcgcta
      601 ccggtcgcca ccatggtgag taaaggagaa gaacttttca ctggagttgt cccaattctt
      661 gttgaattag atggtgatgt taatgggcac aaattttctg tcagtggaga gggtgaaggt
      721 gatgcaacat acggaaaact tacccttaaa tttatttgca ctactggaaa actacctgtt
```

781 ccatggccaa cacttgtcac tactttcact tatggtgttc aatgcttttc
aagataccca
841 gatcatatga aacggcatga ctttttcaag agtgccatgc ccgaaggtta
tgtacaggaa
901 agaactatat ttttcaaaga tgacgggaac tacaagacac gtgctgaagt
caagtttgaa
961 ggtgataccc ttgttaatag aatcgagtta aaaggtattg attttaaaga
agatggaaac
1021 attcttggac acaaattgga atacaactat aactcacaca atgtatacat
catggcagac
1081 aaacaaaaga atggaatcaa agttaacttc aaaattagac acaacattga
agatggaagc
1141 gttcaactag cagaccatta tcaacaaaat actccaattg gcgatggccc
tgtcctttta
1201 ccagacaacc attacctgtc cacacaatct gccctttcga aagatcccaa
cgaaaagaga
1261 gaccacatgg tccttcttga gtttgtaaca gctgctggga ttacacatgg
catggatgaa
1321 ctatacaaat ccggactcag atcttacgat ttaggtgaca ctatagaacg
cggccgccag
1381 gactacaagg accatgacgg tgattacaag gatcatgaca tcgactacaa
ggatgacgat
1441 gacaagcatc atcaccatca ccatggttga ggcgcgcctc ctgcatcaaa
ttaaattgat
1501 taacctatca cattatttat cgtacatcaa tttactattg aaccattatt
tatttattac
1561 atactttgcc tagtatttaa gtcacattat ttcaagtcat aaccgtcatg
aagtcagttg
1621 agattgatta aatacagttc ttatgatcta aattacaaat tattactcca
atcaagactg
1681 aaaactggga aatccgctag ggataacagg gtaatatata agcttcgtac
gtgactgcag
1741 aatgcttcag tattgttttg tatatatata tatatatata tatatataac
taaggcgaat
1801 aatatacaaa gtctcaatcg cggtctacat cataaaagga ccctttagga
ttactaacaa
1861 ccaccttccc agaagatgag gatacgaaat agaaaaaaaa aggttcagct
tttaggggca
1921 gggcatgctc atgtagagcg cctgctcgcc gtccgaggcg gtgccgtcgt
acagggcggt
1981 gtccaggccg cagagggtga accccatccg ccggtacgcg tggatcgccg
gtgcgttgac
2041 gttggtgacc tccagccaga ggtgcccggc gccccgctcg cgggcgaact
ccgtcgcgag
2101 ccccatcaac gcgcgcccga ccccgtgccc ccggtgctcc ggggcgacct
cgatgtcctc
2161 gacggtcagc cggcggttcc agccggagta cgagacgacc acgaagcccg
ccaggtcgcc
2221 gtcgtccccg tacgcgacga acgtccggga gtccgggtcg ccgtcctccc
cgtcgtccga
2281 ttcgtcgtcc gattcgtcgt cggggaacac cttggtcagg ggcgggtcca
ccggcacctc
2341 ccgcagggtg aagccgtccc cggtggcggt gacgcggaag acggtgtcgg
tggtgaagga

2401 cccatccagt gcctcgatgg cctcggcgtc ccccgggaca ctggtgcggt
accggtaagc
2461 cgtgtcgtca agagtggtac ccatttttaa tgttacttct cttgcagtta
gggaactata
2521 atgtaactca aaataagatt aaacaaacta aaataaaaag aagttataca
gaaaaaccca
2581 tataaaccag tactaatcca taataataat acacaaaaaa actatcaaat
aaaaccagaa
2641 aacagattga atagaaaaat tttttcgatc tccttttata ttcaaaattc
gatatatgaa
2701 aaagggaact ctcagaaaat caccaaatca atttaattag atttttcttt
tccttctagc
2761 gttggaaaga aaaattttc tttttttttt tagaaatgaa aaatttttgc
cgtaggaatc
2821 accgtataaa ccctgtataa acgctactct gttcacctgt gtaggctatg
attgacccag
2881 tgttcattgt tattgcgaga gagcgggaga aaagaaccga tacaagagat
ccatgctggt
2941 atagttgtct gtccaacact ttgatgaact tgtaggacga tgatgtgtat
ttagacgagt
3001 acgtgtgtga ctattaagta gttatgatag agaggtttgt acggtgtgtt
ctgtgtaatt
3061 cgattgagaa aatggttatg aatcgagaat actacgccgt ctgatatcag
atccactagt
3121 ggcctatgcg gccgcggatc tgccggtctc cctatagtga gtcgtattac
ccgggatcca
3181 ccggatctag ataactgatc ataatcagcc ataccacatt tgtagaggtt
ttacttgctt
3241 taaaaaacct cccacacctc cccctgaacc tgaaacataa aatgaatgca
attgttgttg
3301 ttaacttgtt tattgcagct tataatggtt acaaataaag caatagcatc
acaaatttca
3361 caaataaagc atttttttca ctgcattcta gttgtggttt gtccaaactc
atcaatgtat
3421 cttaaggcgt aaattgtaag cgttaatatt ttgttaaaat tcgcgttaaa
tttttgttaa
3481 atcagctcat tttttaacca ataggccgaa atcggcaaaa tcccttataa
atcaaaagaa
3541 tagaccgaga tagggttgag tgttgttcca gtttggaaca agagtccact
attaaagaac
3601 gtggactcca acgtcaaagg gcgaaaaacc gtctatcagg gcgatggccc
actacgtgaa
3661 ccatcaccct aatcaagttt tttggggtcg aggtgccgta aagcactaaa
tcggaaccct
3721 aaagggagcc cccgatttag agcttgacgg ggaaagccgg cgaacgtggc
gagaaaggaa
3781 gggaagaaag cgaaaggagc gggcgctagg gcgctggcaa gtgtagcggt
cacgctgcgc
3841 gtaaccacca cacccgccgc gcttaatgcg ccgctacagg gcgcgtcagg
tggcactttt
3901 cggggaaatg tgcgcggaac ccctatttgt ttatttttct aaatacattc
aaatatgtat
3961 ccgctcatga gacaataacc ctgataaatg cttcaataat attgaaaaag
gaagagtcct

4021 gaggcggaaa gaaccagctg tggaatgtgt gtcagttagg gtgtggaaag tccccaggct
4081 ccccagcagg cagaagtatg caaagcatgc atctcaatta gtcagcaacc aggtgtggaa
4141 agtccccagg ctccccagca ggcagaagta tgcaaagcat gcatctcaat tagtcagcaa
4201 ccatagtccc gcccctaact ccgcccatcc cgcccctaac tccgcccagt tccgcccatt
4261 ctccgcccca tggctgacta attttttttа tttatgcaga ggccgaggcc gcctcggcct
4321 ctgagctatt ccagaagtag tgaggaggct tttttggagg cctaggcttt tgcaaagatc
4381 gatcaagaga caggatgagg atcgtttcgc atgattgaac aagatggatt gcacgcaggt
4441 tctccggccg cttgggtgga gaggctattc ggctatgact gggcacaaca gacaatcggc
4501 tgctctgatg ccgccgtgtt ccggctgtca gcgcaggggc gcccggttct ttttgtcaag
4561 accgacctgt ccggtgccct gaatgaactg caagacgagg cagcgcggct atcgtggctg
4621 gccacgacgg gcgttccttg cgcagctgtg ctcgacgttg tcactgaagc gggaagggac
4681 tggctgctat tgggcgaagt gccggggcag gatctcctgt catctcacct tgctcctgcc
4741 gagaaagtat ccatcatggc tgatgcaatg cggcggctgc atacgcttga tccggctacc
4801 tgcccattcg accaccaagc gaaacatcgc atcgagcgag cacgtactcg gatggaagcc
4861 ggtcttgtcg atcaggatga tctggacgaa gagcatcagg ggctcgcgcc agccgaactg
4921 ttcgccaggc tcaaggcgag catgcccgac ggcgaggatc tcgtcgtgac ccatggcgat
4981 gcctgcttgc cgaatatcat ggtggaaaat ggccgctttt ctggattcat cgactgtggc
5041 cggctgggtg tggcggaccg ctatcaggac atagcgttgg ctacccgtga tattgctgaa
5101 gagcttggcg gcgaatgggc tgaccgcttc ctcgtgcttt acggtatcgc cgctcccgat
5161 tcgcagcgca tcgccttcta tcgccttctt gacgagttct tctgagcggg actctggggt
5221 tcgaaatgac cgaccaagcg acgcccaacc tgccatcacg agatttcgat tccaccgccg
5281 ccttctatga aaggttgggc ttcggaatcg ttttccggga cgccggctgg atgatcctcc
5341 agcgcgggga tctcatgctg gagttcttcg cccaccctag ggggaggcta actgaaacac
5401 ggaaggagac aataccggaa ggaacccgcg ctatgacggc aataaaaaga cagaataaaa
5461 cgcacggtgt tgggtcgttt gttcataaac gcggggttcg gtcccagggc tggcactctg
5521 tcgatacccc accgagaccc cattggggcc aatacgcccg cgtttcttcc ttttccccac
5581 cccaccccсc aagttcgggt gaaggcccag ggctcgcagc caacgtcggg gcggcaggcc

5641 ctgccatagc ctcaggttac tcatatatac tttagattga tttaaaactt
catttttaat
5701 ttaaaaggat ctaggtgaag atcctttttg ataatctcat gaccaaaatc
ccttaacgtg
5761 agttttcgtt ccactgagcg tcagaccccg tagaaaagat caaaggatct
tcttgagatc
5821 cttttttct gcgcgtaatc tgctgcttgc aaacaaaaaa accaccgcta
ccagcggtgg
5881 tttgtttgcc ggatcaagag ctaccaactc tttttccgaa ggtaactggc
ttcagcagag
5941 cgcagatacc aaatactgtc cttctagtgt agccgtagtt aggccaccac
ttcaagaact
6001 ctgtagcacc gcctacatac ctcgctctgc taatcctgtt accagtggct
gctgccagtg
6061 gcgataagtc gtgtcttacc gggttggact caagacgata gttaccggat
aaggcgcagc
6121 ggtcgggctg aacggggggt tcgtgcacac agcccagctt ggagcgaacg
acctacaccg
6181 aactgagata cctacagcgt gagctatgag aaagcgccac gcttcccgaa
gggagaaagg
6241 cggacaggta tccggtaagc ggcagggtcg gaacaggaga gcgcacgagg
gagcttccag
6301 ggggaaacgc ctggtatctt tatagtcctg tcgggtttcg ccacctctga
cttgagcgtc
6361 gatttttgtg atgctcgtca ggggggcgga gcctatggaa aaacgccagc
aacgcggcct
6421 ttttacggtt cctggccttt tgctggcctt ttgctcacat gttctttcct
gcgttatccc
6481 ctgattctgt ggataaccgt attaccgcca tgcat
//

Plasmid: Z4EM_RPN11_Mcherry_Sv40NLS to make the inducible Rpn11p nuclear tag

```
LOCUS       Z4EM_RPN11_Mcher       12457 bp    DNA     circular SYN 23-
SEP-2022
DEFINITION  synthetic circular DNA
ACCESSION   .
VERSION     .
KEYWORDS    .
SOURCE      synthetic DNA construct
  ORGANISM  recombinant plasmid
REFERENCE   1  (bases 1 to 12457)
  AUTHORS   Trial User
  TITLE     Direct Submission
  JOURNAL   Exported Dec 22, 2025 from SnapGene 8.2.1
            https://www.snapgene.com
FEATURES             Location/Qualifiers
     source          1..12457
                     /mol_type="other DNA"
                     /organism="recombinant plasmid"
     primer_bind     complement(6..22)
                     /label=M13 fwd
                     /note="common sequencing primer, one of multiple
similar
                     variants"
     primer_bind     6..22
                     /label=Leu2_NCoI_Forward_1
     rep_origin      163..618
                     /direction=RIGHT
                     /label=f1 ori
                     /note="f1 bacteriophage origin of replication; arrow
                     indicates direction of (+) strand synthesis"
     promoter        1256..1360
                     /gene="bla"
                     /label=AmpR promoter
     CDS             join(1361..1429,1430..2221)
                     /codon_start=1
                     /gene="bla"
                     /product="beta-lactamase"
                     /label=AmpR
                     /note="confers resistance to ampicillin,
carbenicillin, and
                     related antibiotics"

/translation="MSIQHFRVALIPFFAAFCLPVFAHPETLVKVKDAEDQLGARVGYI

ELDLNSGKILESFRPEERFPMMSTFKVLLCGAVLSRIDAGQEQLGRRIHYSQNDLVEYS

PVTEKHLTDGMTVRELCSAAITMSDNTAANLLLTTIGGPKELTAFLHNMGDHVTRLDRW

EPELNEAIPNDERDTTMPVAMATTLRKLLTGELLTLASRQQLIDWMEADKVAGPLLRSA

LPAGWFIADKSGAGERGSRGIIAALGPDGKPSRIVVIYTTGSQATMDERNRQIAEIGAS
                     LIKHW"
```

```
     rep_origin      2392..2980
                     /direction=RIGHT
                     /label=ori
                     /note="high-copy-number ColE1/pMB1/pBR322/pUC origin
of
                     replication"
     primer_bind     complement(3131..3152)
                     /label=RPR1_TETR_Reverse_4
     protein_bind    3268..3289
                     /label=CAP binding site
                     /bound_moiety="E. coli catabolite activator protein"
                     /note="CAP binding activates transcription in the
presence
                     of cAMP."
     promoter        join(3304..3309,3310..3327,3328..3334)
                     /label=lac promoter
                     /note="promoter for the E. coli lac operon"
     protein_bind    3342..3358
                     /label=lac operator
                     /bound_moiety="lac repressor encoded by lacI"
                     /note="The lac repressor binds to the lac operator
to
                     inhibit transcription in E. coli. This inhibition
can be
                     relieved by adding lactose or
                     isopropyl-beta-D-thiogalactopyranoside (IPTG)."
     primer_bind     3366..3382
                     /label=M13 rev
                     /note="common sequencing primer, one of multiple
similar
                     variants"
     primer_bind     3386..3402
                     /label=TOP_Check_Forward
     promoter        3403..3421
                     /label=T3 promoter
                     /note="promoter for bacteriophage T3 RNA polymerase"
     primer_bind     3429..3479
                     /label=HO_FSEI_Forward
     misc_feature    3458..3957

                     /note="URA3 forward for homology site cut with PMEI,
FSEI,
                     with NCOI "
     primer_bind     complement(3939..3966)
                     /label=NHEI_HO_Rever
     primer_bind     3956..3992
                     /label=NHEI_prestra_For
     misc_feature    3966..4190
                     /locus_tag="Sc tENO2"
                     /label=Sc tENO2
     primer_bind     4188..4208
                     /label=prestra_seq_for
                     /note="sequencing 2044bp"
     misc_feature    4195..4219
```

```
                     /locus_tag="Con1 scar"
                     /label=Con1 scar
     promoter        4224..4907
                     /locus_tag="pZ4 (-Gal4 site)"
                     /label=pZ4 (-Gal4 site)
     misc_feature    4456..4467
                     /locus_tag="Z4 Binding Site(2)"
                     /label=Z4 Binding Site
                     /label=Z4 Binding Site(2)
                     /label=nonstandard type: Binding
     misc_feature    4469..4480
                     /locus_tag="Z4 Binding Site(1)"
                     /label=Z4 Binding Site
                     /label=Z4 Binding Site(1)
                     /label=nonstandard type: Binding
     misc_feature    4483..4494
                     /locus_tag="Z4 Binding Site(3)"
                     /label=Z4 Binding Site
                     /label=Z4 Binding Site(3)
                     /label=nonstandard type: Binding
     misc_feature    4496..4507
                     /locus_tag="Z4 Binding Site(4)"
                     /label=Z4 Binding Site
                     /label=Z4 Binding Site(4)
                     /label=nonstandard type: Binding
     misc_feature    4510..4521
                     /locus_tag="Z4 Binding Site(5)"
                     /label=Z4 Binding Site
                     /label=Z4 Binding Site(5)
                     /label=nonstandard type: Binding
     misc_feature    4523..4534
                     /locus_tag="Z4 Binding Site"
                     /label=Z4 Binding Site
                     /label=nonstandard type: Binding
     misc_feature    complement(4547..4550)
                     /locus_tag="Bbs1(6)"
                     /label=Bbs1
                     /label=Bbs1(6)
     misc_feature    complement(4553..4558)
                     /locus_tag="Bbs1(4)"
                     /label=Bbs1
                     /label=Bbs1(4)
     promoter        4908..4920
                     /locus_tag="pZ4 (-Gal4 site)(1)"
                     /label=pZ4 (-Gal4 site)
                     /label=pZ4 (-Gal4 site)(1)
     promoter        4921..4947
                     /locus_tag="pZ4 (-Gal4 site)(2)"
                     /label=pZ4 (-Gal4 site)
                     /label=pZ4 (-Gal4 site)(2)
     primer_bind     complement(4922..4965)
                     /label=BSu36I_pestra_Rev
     misc_feature    join(4948..4960,4961..4965)
                     /label=Sequence_insert
```

```
     misc_feature    4954..4959
                     /label=Sequencing_insert
     primer_bind     4957..4995
                     /label=Rpn11_AvrII_mcherry_for
     misc_feature    4966..4971
                     /label=Kozak
     misc_feature    4972..5889
                     /label=RPN11
     primer_bind     5890..5916
                     /label=Forward
     misc_feature    5890..5893
                     /gene="pCMV"
                     /label=pCMV
     primer_bind     5901..5940
                     /label=mCherry-S
     CDS             5917..6624
                     /codon_start=1
                     /gene="mCherry"
                     /label=mCherry

/translation="MVSKGEEDNMAIIKEFMRFKVHMEGSVNGHEFEIEGEGEGRPYEG

TQTAKLKVTKGGPLPFAWDILSPQFMYGSKAYVKHPADIPDYLKLSFPEGFKWERVMNF

EDGGVVTVTQDSSLQDGEFIYKVKLRGTNFPSDGPVMQKKTMGWEASSERMYPEDGALK

GEIKQRLKLKDGGHYDAEVKTTYKAKKPVQLPGAYNVNIKLDITSHNEDYTIVEQYERA
                     EGRHSTGGMDELYK"
     primer_bind     6248..6295
                     /label=mcherry_site_mut_forward
     primer_bind     complement(6248..6280)
                     /label=mcherry_site_directed_reverse
     misc_feature    6268..6270
                     /label=site_mutagenesis
                     /note="original 'ctg' "
     misc_feature    6274..6276
                     /label=site_mutagensis
                     /note="gac"
     primer_bind     6628..6699
                     /label=Sv40_HR_For
     primer_bind     complement(6656..6675)
                     /label=Rpn11_Mcherry_rev
                     /note="ctggcggccgcgttctatag"
     CDS             6688..6708
                     /codon_start=1
                     /product="nuclear localization signal of SV40
(simian virus
                     40) large T antigen"
                     /label=SV40 NLS
                     /translation="PKKKRKV"
     CDS             6712..6732
                     /codon_start=1
                     /product="nuclear localization signal of SV40
(simian virus
```

```
                     40) large T antigen"
                     /label=SV40 NLS
                     /translation="PKKKRKV"
     primer_bind     6733..6747
                     /label=Sv40_HR_For
     CDS             6736..6756
                     /codon_start=1
                     /product="nuclear localization signal of SV40
(simian virus
                     40) large T antigen"
                     /label=SV40 NLS
                     /translation="PKKKRKV"
     primer_bind     complement(6745..6815)
                     /label=SV40_HR_Adh1_rev
     primer_bind     6771..6791
                     /label=tAdh1_term_for
     terminator      6779..6981
                     /gene="tADH1"
     primer_bind     complement(6963..6996)
                     /label=tAdh1_term_SBFI_Rev
     primer_bind     complement(6965..6996)
                     /label=SBFI_Mcherry_Reverse
     primer_bind     6985..7014
                     /label=SBFI_Estro_For
     misc_feature    6992..6993
                     /locus_tag="Con1 scar"
                     /label=Con1 scar
     misc_feature    6994..7693
                     /locus_tag="Sc pRET2"
                     /label=Sc pRET2
     misc_feature    7700..8050
                     /locus_tag="pZ4 DNA Binding Domain"
                     /label=pZ4 DNA Binding Domain
                     /label=nonstandard type:  ORF
     misc_feature    8081..9022
                     /locus_tag="hER-LBD"
                     /label=hER-LBD
     misc_feature    9056..9979
                     /locus_tag="Msn2 Activation Domain (27 more nt's,
encoding
                     FPSMTNSRN)"
                     /label=Msn2 Activation Domain (27 more nt's,
                     encoding ...
                     /label=Msn2 Activation Domain (27 more nt's,
encoding
                     FPSMTNSRN)
                     /label=nonstandard type:  ORF
     primer_bind     complement(9960..9991)
                     /label=AFIII_Estro_RE_Rev
     primer_bind     complement(9960..9980)
                     /label=estra_seq_reverse
                     /note="3008bp"
     misc_feature    9985..10382
                     /label=tGMP1_Termin
```

```
     primer_bind     complement(10478..10505)
                     /label=RPR1_TETR_Reverse_4
     gene            10506..11862
                     /label=kanMX
                     /note="yeast selectable marker conferring kanamycin
                     resistance (Wach et al., 1994)"
     promoter        10506..10849
                     /label=TEF promoter
                     /note="Ashbya gossypii TEF promoter"
     CDS             10850..11659
                     /codon_start=1
                     /gene="aph(3')-Ia"
                     /product="aminoglycoside phosphotransferase"
                     /label=KanR
                     /note="confers resistance to kanamycin"

/translation="MGKEKTHVSRPRLNSNMDADLYGYKWARDNVGQSGATIYRLYGKP

DAPELFLKHGKGSVANDVTDEMVRLNWLTEFMPLPTIKHFIRTPDDAWLLTTAIPGKTA

FQVLEEYPDSGENIVDALAVFLRRLHSIPVCNCPFNSDRVFRLAQAQSRMNNGLVDASD

FDDERNGWPVEQVWKEMHKLLPFSPDSVVTHGDFSLDNLIFDEGKLIGCIDVGRVGIAD
                     RYQDLAILWNCLGEFSPSLQKRLFQKYGIDNPDMNKLQFHLMLDEFF"
     terminator      11665..11862
                     /label=TEF terminator
                     /note="Ashbya gossypii TEF terminator"
     primer_bind     11863..11912
                     /label=HO_NOTI_For
     misc_feature    complement(11871..11889)
                     /label=T7 promoter
     primer_bind     complement(11871..11889)
                     /label=NAT_Reverse_5
                     /note="its been butched.
                     gaacttcgggcacagaatactttaatacgactcactatagggagac"
     misc_feature    11926..12425
                     /label=HO_End
                     /note="This will go downstream of the URA3 site and
remove
                     it
                     to insert the plasmid cut with NCOI with eitehr PMEI
or
                     FSEI. Or just use FSEI and PEMI"
     primer_bind     complement(12410..12457)
                     /label=HO_NCOI_Rev
     primer_bind     complement(12441..12457)
                     /label=End_Reverse_check
                     /note="To check sequence"
ORIGIN
        1 gcttcactgg ccgtcgtttt acaacgtcgt gactgggaaa accctggcgt
tacccaactt
       61 aatcgccttg cagcacatcc cccttttcgcc agctggcgta atagcgaaga
ggcccgcacc
```

121 gatcgccctt cccaacagtt gcgcagcctg aatggcgaat ggacgcgccc tgtagcggcg
181 cattaagcgc ggcgggtgtg gtggttacgc gcagcgtgac cgctacactt gccagcgccc
241 tagcgcccgc tcctttcgct ttcttccctt cctttctcgc cacgttcgcc ggctttcccc
301 gtcaagctct aaatcggggg ctccctttag ggttccgatt tagtgcttta cggcacctcg
361 accccaaaaa acttgattag ggtgatggtt cacgtagtgg gccatcgccc tgatagacgg
421 tttttcgccc tttgacgttg gagtccacgt tctttaatag tggactcttg ttccaaactg
481 gaacaacact caaccctatc tcggtctatt cttttgattt ataagggatt ttgccgattt
541 cggcctattg gttaaaaaat gagctgattt aacaaaaatt taacgcgaat tttaacaaaa
601 tattaacgct tacaatttcc tgatgcggta ttttctcctt acgcatctgt gcggtatttc
661 acaccgcata gggtaataac tgatataatt aaattgaagc tctaatttgt gagtttagta
721 ttaggatctc tacccttggc gaaaagtcct ctgccaacaa tgatgatatc tgatccacca
781 cttacaactt cgtcgacggt tctgtactgc tgacccaata tgcgtatata taccaatcta
841 agtctgtgct ccttccttcg ttcttccttc tgttcggaga ttaccgaatc aaaaaaattt
901 caaggaaacc gaaatcaaaa aaaagaataa aaaaaaaatg atgaattgaa aaggtggtat
961 ggtgcactct cagtacaatc tgctctgatg ccgcatagtt aagccagccc cgacacccgc
1021 caacacccgc tgacgcgccc tgacgggctt gtctgctccc ggcatccgct tacagacaag
1081 ctgtgaccgt ctccgggagc tgcatgtgtc agaggttttc accgtcatca ccgaaacgcg
1141 cgagacgaaa gggcctcgtg atacgcctat ttttataggt taatgtcatg ataataatgg
1201 tttcttagac gtcggcgcgc cgaggacgtc aggtggcact tttcggggaa atgtgcgcgg
1261 aacccctatt tgtttatttt tctaaataca ttcaaatatg tatccgctca tgagacaata
1321 accctgataa atgcttcaat aatattgaaa aaggaagagt atgagtattc aacatttccg
1381 tgtcgccctt attccctttt ttgcggcatt ttgccttcct gtttttgctc acccagaaac
1441 gctggtgaaa gtaaaagatg ctgaagatca gttgggtgca cgagtgggtt acatcgaact
1501 ggatctcaac agcggtaaga tccttgagag ttttcgcccc gaagaacgtt ttccaatgat
1561 gagcactttt aaagttctgc tatgtggcgc ggtattatcc cgtattgacg ccgggcaaga
1621 gcaactcggt cgccgcatac actattctca gaatgacttg gttgagtact caccagtcac
1681 agaaaagcat cttacggatg gcatgacagt aagagaatta tgcagtgctg ccataaccat

1741 gagtgataac actgcggcca acttacttct gacaacgatc ggaggaccga aggagctaac
1801 cgcttttttg cacaacatgg gggatcatgt aactcgcctt gatcgttggg aaccggagct
1861 gaatgaagcc ataccaaacg acgagcgtga caccacgatg cctgtagcaa tggcaacaac
1921 gttgcgcaaa ctattaactg gcgaactact tactctagct tcccggcaac aattaataga
1981 ctggatggag gcggataaag ttgcaggacc acttctgcgc tcggcccttc cggctggctg
2041 gtttattgct gataaatctg gagccggtga gcgtgggtct cgcggtatca ttgcagcact
2101 ggggccagat ggtaagccct cccgtatcgt agttatctac acgacgggga gtcaggcaac
2161 tatggatgaa cgaaatagac agatcgctga gataggtgcc tcactgatta agcattggta
2221 actgtcagac caagtttact catatatact ttagattgat ttaaaacttc atttttaatt
2281 taaaaggatc taggtgaaga tcctttttga taatctcatg accaaaatcc cttaacgtga
2341 gttttcgttc cactgagcgt cagaccccgt agaaaagatc aaaggatctt cttgagatcc
2401 tttttttctg cgcgtaatct gctgcttgca aacaaaaaaa ccaccgctac cagcggtggt
2461 ttgtttgccg gatcaagagc taccaactct ttttccgaag gtaactggct tcagcagagc
2521 gcagatacca aatactgttc ttctagtgta gccgtagtta ggccaccact tcaagaactc
2581 tgtagcaccg cctacatacc tcgctctgct aatcctgtta ccagtggctg ctgccagtgg
2641 cgataagtcg tgtcttaccg ggttggactc aagacgatag ttaccggata aggcgcagcg
2701 gtcgggctga acggggggtt cgtgcacaca gcccagcttg gagcgaacga cctacaccga
2761 actgagatac ctacagcgtg agctatgaga aagcgccacg cttcccgaag ggagaaaggc
2821 ggacaggtat ccggtaagcg gcagggtcgg aacaggagag cgcacgaggg agcttccagg
2881 gggaaacgcc tggtatcttt atagtcctgt cgggtttcgc cacctctgac ttgagcgtcg
2941 atttttgtga tgctcgtcag gggggcggag cctatggaaa aacgccagca acgcggcctt
3001 tttacggttc ctggcctttt gctggccttt tgctcacatg ttctttcctg cgttatcccc
3061 tgattctgtg gataaccgta ttaccgcctt tgagtgagct gataccgctc gccgcagccg
3121 aacgaccgag cgcagcgagt cagtgagcga ggaagcggaa gagcgcccaa tacgcaaacc
3181 gcctctcccc gcgcgttggc cgattcatta atgcagctgg cacgacaggt ttcccgactg
3241 gaaagcgggc agtgagcgca acgcaattaa tgtgagttag ctcactcatt aggcacccca
3301 ggctttacac tttatgcttc cggctcctat gttgtgtgga attgtgagcg gataacaatt

3361 tcacacagga aacagctatg accatgatta cgccaagctc ggaattaacc ctcactaaag
3421 ggaacaaaag ctgggccggc catttaaatg tttaaacgct aaattcgagt gaaacacagg
3481 aagatcagaa aatcctcatt tcatccatat taacaataat ttcaaatgtt tatttgcatt
3541 atttgaaact aggcaagaca agcaacgaaa cgtttttgaa aattttgagt attttcaata
3601 aatttgtaga ggactcagat attgaaaaaa agctacagca attaatactt gataagaaga
3661 gtattgagaa gggcaacggt tcatcatctc atggatctgc acatgaacaa acaccagagt
3721 caaacgacgt tgaaattgag gctactgcgc caattgatga caatacagac gatgataaca
3781 aaccgaagtt atctgatgta gaaaaggatt aaagatgcta agagatagtg atgatatttc
3841 ataaataatg taattctata tatgttaatt acctttttttg cgaggcatat ttatggtgaa
3901 ggataagttt tgaccatcaa agaaggttaa tgtggctgtg gtttcagggt ccataaagcg
3961 ctagcagtgc ttttaactaa gaattattag tcttttctgc ttattttttc atcatagttt
4021 agaacacttt atattaacga atagtttatg aatctattta ggtttaaaaa ttgatacagt
4081 tttataagtt actttttcaa agactcgtgc tgtctattgc ataatgcact ggaaggggaa
4141 aaaaaaggtg cacacgcgtg gctttttctt gaatttgcag tttgaaaaat gctgccaaac
4201 cagatgtcaa cacagctaca acgttatatt gaattttcaa aaattcttac tttttttttg
4261 gatggacgca aagaagttta ataatcatat tacatggcat taccaccata tacatatcca
4321 tatacatatc catatctaat cttacttata tgttgtggaa atgtaaagag ccccattatc
4381 ttagcctaaa aaaaccttct ctttggaact ttcagtaata cgcttaactg ctcattgcta
4441 tattgaagtg tggccgcggc ggaggagtgc ggcggaggag gagcggcgga ggagtgcggc
4501 ggaggaggag cggcggagga gtgcggcgga ggagtctaga aattgcgtcc tcgtcttcac
4561 cggtcgcgtt cctgaaacgc agatgtgcct aatgccgcac tgctccgaac aataaagatt
4621 ctacaatact agcttttatg gttatgaaga ggaaaaattg gcagtaacct ggccccacaa
4681 accttcaaat taacgaatca aattaacaac cataggatga taatgcgatt agtttttttag
4741 ccttatttct ggggtaatta atcagcgaag cgatgatttt tgatctatta acagatatat
4801 aaatggaaaa gctgcataac cactttaact aatactttca acattttcag tttgtattac
4861 ttcttattca aatgtcataa aagtatcaac aaaaaattgt taatatacct ctatacttta
4921 acgtcaagga gaaaaaacta taagatccac gtgagctgcc ctagggccac catggaacga

4981 ctacagagat tgatgatgaa tagtaaggtg gggtccgcgg acaccggccg
tgacgatacg
5041 aaagaaactg tttatatttc ttcgattgcg cttttaaaga tgctaaagca
tggtagagct
5101 ggtgttccca tggaagtcat ggggttgatg ttaggtgagt ttgtcgatga
ttatacggtt
5161 aacgttgtgg acgtgtttgc gatgcctcaa tcgggtaccg gagtttctgt
tgaggctgtc
5221 gatgatgttt tccaagcgaa gatgatggac atgttaaaac aaacgggcag
agaccaaatg
5281 gtcgttggct ggtaccactc tcatccaggg tttggctgtt ggctatcttc
tgttgatgtt
5341 aatactcaaa aatcttttga acaactaaac agcagagctg ttgctgtcgt
tgttgaccct
5401 attcaatccg ttaagggaaa agttgtcatc gatgctttta gattgattga
cacgggcgca
5461 ttgataaata acttagagcc tagacaaaca acctccaaca caggcttatt
gaacaaggcc
5521 aacattcaag ccttaattca cggtctgaat aggcattact attctttaaa
tattgattat
5581 cataaaaccg cgaaggaaac caagatgtta atgaacttac ataaagaaca
gtggcaatca
5641 ggtcttaaga tgtacgatta tgaagaaaaa gaagaatcaa atttggctgc
tacaaagagt
5701 atggttaaga tagccgaaca gtactctaag agaatagaag aggaaaagga
attaaccgaa
5761 gaagaactta agacaagata cgttggtagg caagatccaa agaagcacct
ttccgaaaca
5821 gcagatgaga cactagagaa caatattgtt tctgtgctga cggcgggtgt
taattcagtg
5881 gcaattaaag atccgctagc gctaccggtc gccaccatgg tgagcaaggg
cgaggaggat
5941 aacatggcca tcatcaagga gttcatgcgc ttcaaggtgc acatggaggg
ctccgtgaac
6001 ggccacgagt tcgagatcga gggcgagggc gagggccgcc cctacgaggg
cacccagacc
6061 gccaagctga aggtgaccaa gggtggcccc ctgcccttcg cctgggacat
cctgtcccct
6121 cagttcatgt acggctccaa ggcctacgtg aagcaccccg ccgacatccc
cgactacttg
6181 aagctgtcct tccccgaggg cttcaagtgg gagcgcgtga tgaacttcga
ggacggcggc
6241 gtggtgaccg tgacccagga ctcctccttg caggatggcg agttcatcta
caaggtgaag
6301 ctgcgcggca ccaacttccc ctccgacggc cccgtaatgc agaagaagac
catgggctgg
6361 gaggcctcct ccgagcggat gtaccccgag gacggcgccc tgaagggcga
gatcaagcag
6421 aggctgaagc tgaaggacgg cggccactac gacgctgagg tcaagaccac
ctacaaggcc
6481 aagaagcccg tgcagctgcc cggcgcctac aacgtcaaca tcaagttgga
catcacctcc
6541 cacaacgagg actacaccat cgtggaacag tacgaacgcg ccgagggccg
ccactccacc

6601 ggcggcatgg acgagctgta caagtccgga ctcagatctt acgatttagg
tgacactata
6661 gaacgcggcc gccaggaggg agctgatcca aaaaagaaga gaaaggtaga
tccaaaaaag
6721 aagagaaagg tagatccaaa aaagaagaga aaggtataga attctaccgg
ggcgcgccac
6781 ttctaaataa gcgaatttct tatgatttat gatttttatt attaaataag
ttataaaaaa
6841 aataagtgta tacaaatttt aaagtgactc ttaggtttta aaacgaaaat
tcttattctt
6901 gagtaactct ttcctgtagg tcaggttgct ttctcaggta tagtatgagg
tcgctcttat
6961 tgaccacacc tctaccggca gatcccctgc aggacgatgg cttcttatct
cacttcaata
7021 gtactttcca ccggttatac ttccggcttt tccctattaa tacaagctac
aatttcaatg
7081 ggtggcaaat aatgtgtaga atagaaaata agccgacagg gtaataaaga
aaatttttag
7141 aaaaaaaagg ttagatggct tatttaagtt acaggctagc gaaaaaagga
acttcagggc
7201 aagtaaagtg tttgattggg cactagcatg gcttataaag gcgagcaatt
gtcgaaacta
7261 attaatgttg tacggactat tgctgtcatc tcgtggtaaa tgcgtgttcc
aggtcgaata
7321 ctacttgcac acaggcgagc ggggccccat aaaagtgttg ccgatttgtt
aagttgtctt
7381 ttcggttttt ctactctgtt attccttact tccctttta agaactcttt
ttatccttca
7441 tttaggatct tgcacgtttc cgcctcatca cttgaattaa aacatgtctc
tgtcagtaaa
7501 ccttggcgtt tctattgttc ttcatagttc aacttttatt attacccgcc
ctgcgcgttt
7561 acattttcc agcaacagcc agcgaaaaat tagaaaatct ggttgttgac
acctcaagaa
7621 caagggcaat tagcctcagc gtcgaatata gatcatatta gaatacctat
agctccatca
7681 aaagaaatac acaagatcta tgggtacccg cccatatgct tgccctgtcg
agtcctgcga
7741 tcgccgcttt tctcgccacg ccaatcttac ccgccatatc cgcatccata
ccggtcagaa
7801 gcccttccag tgtcgaatct gcatgcgtaa cttcagtcgt aatgcgaacc
ttgtgcgcca
7861 catccgcacc cacacaggat cccaaaagcc gttccaatgt cggatctgta
tgcggaactt
7921 tagtcgaaag gccgacctga ggcgtcacat tcgcacgcac accggcgaga
agccttttgc
7981 ctgtgacatt tgtgggagga agtttgccag gaagggcgac ctcaagaggc
ataccaaaat
8041 ccatacaggt agatccggtg acggtgctgg tttaattaac tctgctggag
acatgagagc
8101 tgccaacctt tggccaagcc cgctcatgat caaacgctct aagaagaaca
gcctggcctt
8161 gtccctgacg gccgaccaga tggtcagtgc cttgttggat gctgagcccc
ccatactcta

8221 ttccgagtat gatcctacca gacccttcag tgaagcttcg atgatgggct
tactgaccaa
8281 cctggcagac agggagctgg ttcacatgat caactgggcg aagagggtgc
caggctttgt
8341 ggatttgacc ctccatgatc aggtccacct tctagaatgt gcctggctag
agatcctgat
8401 gattggactc gtctggcgct ccatggagca cccagggaag ctactgtttg
ctcctaactt
8461 gctcttggac aggaaccagg gaaaatgtgt agagggcatg gtggagatct
tcgacatgct
8521 gctggctaca tcatctcggt tccgcatgat gaatctgcag ggagaggagt
ttgtgtgcct
8581 caaatctatt attttgctta attctggagt gtacacattt ctgtccagca
ccctgaagtc
8641 tctggaagag aaggaccata tccaccgagt cctggacaag atcacagaca
ctttgatcca
8701 cctgatggcc aaggcaggcc tgaccctgca gcagcagcac cagcggctgg
cccagctcct
8761 cctcatcctc tcccacatca ggcacatgag taacaaaggc atggagcatc
tgtacagcat
8821 gaagtgcaag aacgtggtgc ccctctatga cctgctgctg gagatgctgg
acgcccaccg
8881 cctacatgcg cccactagcc gtggaggggc atccgtggag gaaacggacc
aaagccactt
8941 ggccactgcg ggctctactt catcgcattc cttgcaaaag tattacatca
cgggggaggc
9001 agagggtttc cctgccacag tcgcggctgc aggtgacggt gctggtttaa
ttaacatgac
9061 ggtcgaccat gatttcaata gcgaagatat tttattcccc atagaaagca
tgagtagtat
9121 acaatacgtg gagaataata acccaaataa tattaacaac gatgttatcc
cgtattctct
9181 agatatcaaa aacactgtct tagatagtgc ggatctcaat gacattcaaa
atcaagaaac
9241 ttcactgaat ttggggcttc ctccactatc tttcgactct ccactgcccg
taacggaaac
9301 gataccatcc actaccgata acagcttgca tttgaaagct gatagcaaca
aaaatcgcga
9361 tgcaagaact attgaaaatg atagtgaaat taagagtact aataatgcta
gtggctctgg
9421 ggcaaatcaa tacacaactc ttacttcacc ttatcctatg aacgacattt
tgtacaacat
9481 gaacaatccg ttacaatcac cgtcaccttc atcggtacct caaaatccga
ctataaatcc
9541 tcccataaat acagcaagta acgaaactaa tttatcgcct caaacttcaa
atggtaatga
9601 aactcttata tctcctcgag cccaacaaca tacgtccatt aaagataatc
gtctgtcctt
9661 acctaatggt gctaattcga atcttttcat tgacactaac ccaaacaatt
tgaacgaaaa
9721 actaagaaat caattgaact cagatacaaa ttcatattct aactccattt
ctaattcaaa
9781 ctccaattct acgggtaatt taaattccag ttattttaat tcactgaaca
tagactccat

9841 gctagatgat tacgtttcta gtgatctctt attgaatgat gatgatgatg acactaattt
9901 atcacgccga agatttagcg acgttataac aaaccaattt ccgtcaatga caaattcgag
9961 gaatgagctc ggatcctaac ttaagtctga agaatgaatg atttgatgat ttctttttcc
10021 ctccattttt cttactgaat atatcaatga tatagacttg tatagtttat tatttcaaat
10081 taagtagcta tatatagtca agataacgtt tgtttgacac gattacatta ttcgtcgaca
10141 tctttttca gcctgtcgtg gtagcaattt gaggagtatt attaattgaa taggttcatt
10201 ttgcgctcgc ataaacagtt ttcgtcaggg acagtatgtt ggaatgagtg gtaattaatg
10261 gtgacatgac atgttatagc aataaccttg atgtttacat cgtagtttaa tgtacacccc
10321 gcgaattcgt tcaagtagga gtgcaccaat tgcaaaggga aaagctgaat gggcagttcg
10381 aaccgcggtc tttcctgcgt tatcccctga ttctgtggat aaccgtatta ccgcctttga
10441 gtgagctgat accgctcgcc gcagccgaac gaccgagcgc agcgagtcag tgagcgaggc
10501 tgagcgacat ggaggcccag aataccctcc ttgacagtct tgacgtgcgc agctcagggg
10561 catgatgtga ctgtcgcccg tacatttagc ccatacatcc ccatgtataa tcatttgcat
10621 ccatacattt tgatggccgc acggcgcgaa gcaaaaatta cggctcctcg ctgcagacct
10681 gcgagcaggg aaacgctccc ctcacagacg cgttgaattg tccccacgcc gcgcccctgt
10741 agagaaatat aaaaggttag gatttgccac tgaggttctt ctttcatata cttcctttta
10801 aaatcttgct aggatacagt tctcacatca catccgaaca taaacaacca tgggtaagga
10861 aaagactcac gtttcgaggc cgcgattaaa ttccaacatg gatgctgatt tatatgggta
10921 taaatgggct cgcgataatg tcgggcaatc aggtgcgaca atctatcgat tgtatgggaa
10981 gcccgatgcg ccagagttgt ttctgaaaca tggcaaaggt agcgttgcca atgatgttac
11041 agatgagatg gtcagactaa actggctgac ggaatttatg cctcttccga ccatcaagca
11101 ttttatccgt actcctgatg atgcatggtt actcaccact gcgatccccg gcaaaaacagc
11161 attccaggta ttagaagaat atcctgattc aggtgaaaat attgttgatg cgctggcagt
11221 gttcctgcgc cggttgcatt cgattcctgt ttgtaattgt ccttttaaca gcgatcgcgt
11281 atttcgtctc gctcaggcgc aatcacgaat gaataacggt ttggttgatg cgagtgattt
11341 tgatgacgag cgtaatggct ggcctgttga acaagtctgg aaagaaatgc ataagctttt
11401 gccattctca ccggattcag tcgtcactca tggtgatttc tcacttgata accttatttt

11461 tgacgagggg aaattaatag gttgtattga tgttggacga gtcggaatcg cagaccgata
11521 ccaggatctt gccatcctat ggaactgcct cggtgagttt tctccttcat tacagaaacg
11581 gctttttcaa aaatatggta ttgataatcc tgatatgaat aaattgcagt ttcatttgat
11641 gctcgatgag tttttctaat cagtactgac aataaaaaga ttcttgtttt caagaacttg
11701 tcatttgtat agttttttta tattgtagtt gttctatttt aatcaaatgt tagcgtgatt
11761 tatatttttt ttcgcctcga catcatctgc ccagatgcga agttaagtgc gcagaaagta
11821 atatcatgcg tcaatcgtat gtgaatgctg gtcgctatac tggcggccgc cctatagtga
11881 gtcgtattag agttattacc cgagtagagc acttgaatcc actgccccgg gaatctcggt
11941 cgtaatgatt tctataatga cgaaaaaaaa aaaattggaa agaaaaagct tcatggcctt
12001 tataaaaagg aactatccaa tacctcgcca gaaccaagta acagtatttt acggggcaca
12061 aatcaagaac aataagacag gactgtaaag atggacgcat tgaactccaa agaacaacaa
12121 gagttccaaa aagtagtgga acaaaagcaa atgaaggatt tcatgcgttt gtactctaat
12181 ctggtagaaa gatgtttcac agactgtgtc aatgacttca caacatcaaa gctaaccaat
12241 aaggaacaaa catgcatcat gaagtgctca gaaaagttct tgaagcatag cgaacgtgta
12301 gggcagcgtt tccaagaaca aaacgctgcc ttgggacaag gcttgggccg ataaggtgta
12361 ctggcgtata tatatctaat tatgtatctc tggtgtagcc catttttagc atgtaaatat
12421 aaagaccgag cgggccggcc tcgggccatg gtcactg
//

Plasmid: Z4EM_RPN11_Mcherry for the inducible Rpn11p-mcherry

```
LOCUS       Z4EM_RPN11_Mcher       12446 bp    DNA     circular SYN 30-
JUN-2022
DEFINITION  synthetic circular DNA
ACCESSION   .
VERSION     .
KEYWORDS    .
SOURCE      synthetic DNA construct
  ORGANISM  recombinant plasmid
REFERENCE   1  (bases 1 to 12446)
  AUTHORS   Trial User
  TITLE     Direct Submission
  JOURNAL   Exported Dec 22, 2025 from SnapGene 8.2.1
            https://www.snapgene.com
FEATURES             Location/Qualifiers
     source          1..12446
                     /mol_type="other DNA"
                     /organism="recombinant plasmid"
     primer_bind     complement(6..22)
                     /label=M13 fwd
                     /note="common sequencing primer, one of multiple 
similar
                     variants"
     primer_bind     6..22
                     /label=Leu2_NCoI_Forward_1
     rep_origin      163..618
                     /direction=RIGHT
                     /label=f1 ori
                     /note="f1 bacteriophage origin of replication; arrow
                     indicates direction of (+) strand synthesis"
     promoter        1256..1360
                     /gene="bla"
                     /label=AmpR promoter
     CDS             join(1361..1429,1430..2221)
                     /codon_start=1
                     /gene="bla"
                     /product="beta-lactamase"
                     /label=AmpR
                     /note="confers resistance to ampicillin, 
carbenicillin, and
                     related antibiotics"

/translation="MSIQHFRVALIPFFAAFCLPVFAHPETLVKVKDAEDQLGARVGYI

ELDLNSGKILESFRPEERFPMMSTFKVLLCGAVLSRIDAGQEQLGRRIHYSQNDLVEYS

PVTEKHLTDGMTVRELCSAAITMSDNTAANLLLTTIGGPKELTAFLHNMGDHVTRLDRW

EPELNEAIPNDERDTTMPVAMATTLRKLLTGELLTLASRQQLIDWMEADKVAGPLLRSA

LPAGWFIADKSGAGERGSRGIIAALGPDGKPSRIVVIYTTGSQATMDERNRQIAEIGAS
                     LIKHW"
     rep_origin      2392..2980
```

```
                     /direction=RIGHT
                     /label=ori
                     /note="high-copy-number ColE1/pMB1/pBR322/pUC origin
of
                     replication"
     primer_bind     complement(3131..3152)
                     /label=RPR1_TETR_Reverse_4
     protein_bind    3268..3289
                     /label=CAP binding site
                     /bound_moiety="E. coli catabolite activator protein"
                     /note="CAP binding activates transcription in the
presence
                     of cAMP."
     promoter        join(3304..3309,3310..3327,3328..3334)
                     /label=lac promoter
                     /note="promoter for the E. coli lac operon"
     protein_bind    3342..3358
                     /label=lac operator
                     /bound_moiety="lac repressor encoded by lacI"
                     /note="The lac repressor binds to the lac operator
to
                     inhibit transcription in E. coli. This inhibition
can be
                     relieved by adding lactose or
                     isopropyl-beta-D-thiogalactopyranoside (IPTG)."
     primer_bind     3366..3382
                     /label=M13 rev
                     /note="common sequencing primer, one of multiple
similar
                     variants"
     primer_bind     3386..3402
                     /label=TOP_Check_Forward
     promoter        3403..3421
                     /label=T3 promoter
                     /note="promoter for bacteriophage T3 RNA polymerase"
     primer_bind     3429..3479
                     /label=HO_FSEI_Forward
     misc_feature    3458..3957

                     /note="URA3 forward for homology site cut with PMEI,
FSEI,
                     with NCOI "
     primer_bind     complement(3939..3966)
                     /label=NHEI_HO_Rever
     primer_bind     3956..3992
                     /label=NHEI_prestra_For
     misc_feature    3966..4190
                     /locus_tag="Sc tENO2"
                     /label=Sc tENO2
     primer_bind     4188..4208
                     /label=prestra_seq_for
                     /note="sequencing 2044bp"
     misc_feature    4195..4219
                     /locus_tag="Con1 scar"
```

```
                /label=Con1 scar
promoter        4224..4907
                /locus_tag="pZ4 (-Gal4 site)"
                /label=pZ4 (-Gal4 site)
misc_feature    4456..4467
                /locus_tag="Z4 Binding Site(2)"
                /label=Z4 Binding Site
                /label=Z4 Binding Site(2)
                /label=nonstandard type: Binding
misc_feature    4469..4480
                /locus_tag="Z4 Binding Site(1)"
                /label=Z4 Binding Site
                /label=Z4 Binding Site(1)
                /label=nonstandard type: Binding
misc_feature    4483..4494
                /locus_tag="Z4 Binding Site(3)"
                /label=Z4 Binding Site
                /label=Z4 Binding Site(3)
                /label=nonstandard type: Binding
misc_feature    4496..4507
                /locus_tag="Z4 Binding Site(4)"
                /label=Z4 Binding Site
                /label=Z4 Binding Site(4)
                /label=nonstandard type: Binding
misc_feature    4510..4521
                /locus_tag="Z4 Binding Site(5)"
                /label=Z4 Binding Site
                /label=Z4 Binding Site(5)
                /label=nonstandard type: Binding
misc_feature    4523..4534
                /locus_tag="Z4 Binding Site"
                /label=Z4 Binding Site
                /label=nonstandard type: Binding
misc_feature    complement(4547..4550)
                /locus_tag="Bbs1(6)"
                /label=Bbs1
                /label=Bbs1(6)
misc_feature    complement(4553..4558)
                /locus_tag="Bbs1(4)"
                /label=Bbs1
                /label=Bbs1(4)
promoter        4908..4920
                /locus_tag="pZ4 (-Gal4 site)(1)"
                /label=pZ4 (-Gal4 site)
                /label=pZ4 (-Gal4 site)(1)
promoter        4921..4947
                /locus_tag="pZ4 (-Gal4 site)(2)"
                /label=pZ4 (-Gal4 site)
                /label=pZ4 (-Gal4 site)(2)
primer_bind     complement(4922..4965)
                /label=BSu36I_pestra_Rev
misc_feature    join(4948..4960,4961..4965)
                /label=Sequence_insert
misc_feature    4954..4959
```

```
                     /label=Sequencing_insert
     misc_feature    4966..5883
                     /label=RPN11
     primer_bind     5884..5910
                     /label=Forward
     misc_feature    5884..5887
                     /gene="pCMV"
                     /label=pCMV
     primer_bind     5895..5934
                     /label=mCherry-S
     CDS             5911..6618
                     /codon_start=1
                     /gene="mCherry"
                     /label=mCherry

/translation="MVSKGEEDNMAIIKEFMRFKVHMEGSVNGHEFEIEGEGEGRPYEG

TQTAKLKVTKGGPLPFAWDILSPQFMYGSKAYVKHPADIPDYLKLSFPEGFKWERVMNF

EDGGVVTVTQDSSLQDGEFIYKVKLRGTNFPSDGPVMQKKTMGWEASSERMYPEDGALK

GEIKQRLKLKDGGHYDAEVKTTYKAKKPVQLPGAYNVNIKLDITSHNEDYTIVEQYERA
                     EGRHSTGGMDELYK"
     primer_bind     6242..6289
                     /label=mcherry_site_mut_forward
     primer_bind     complement(6242..6274)
                     /label=mcherry_site_directed_reverse
     misc_feature    6262..6264
                     /label=site_mutagenesis
                     /note="original 'ctg' "
     misc_feature    6268..6270
                     /label=site_mutagensis
                     /note="gac"
     primer_bind     complement(6598..6636)
                     /label=mCherry-A
     primer_bind     6634..6657
                     /label=FT-S
     CDS             6670..6759
                     /codon_start=1
                     /gene="3F6H"
                     /label=3F6H
                     /translation="DYKDHDGDYKDHDIDYKDDDDKHHHHHHG"
     primer_bind     complement(6739..6759)
                     /label=SBFI_REverse
     primer_bind     6741..6783
                     /label=tADH1-S
     terminator      6768..6970
                     /gene="tADH1"
     primer_bind     complement(6954..6985)
                     /label=SBFI_Mcherry_Reverse
     primer_bind     6974..7003
                     /label=SBFI_Estro_For
     misc_feature    6981..6982
                     /locus_tag="Con1 scar"
```

```
                     /label=Con1 scar
     misc_feature    6983..7682
                     /locus_tag="Sc pRET2"
                     /label=Sc pRET2
     misc_feature    7689..8039
                     /locus_tag="pZ4 DNA Binding Domain"
                     /label=pZ4 DNA Binding Domain
                     /label=nonstandard type:  ORF
     misc_feature    8070..9011
                     /locus_tag="hER-LBD"
                     /label=hER-LBD
     misc_feature    9045..9968
                     /locus_tag="Msn2 Activation Domain (27 more nt's,
encoding
                     FPSMTNSRN)"
                     /label=Msn2 Activation Domain (27 more nt's,
                     encoding ...
                     /label=Msn2 Activation Domain (27 more nt's,
encoding
                     FPSMTNSRN)
                     /label=nonstandard type:  ORF
     primer_bind     complement(9949..9980)
                     /label=AFIII_Estro_RE_Rev
     primer_bind     complement(9949..9969)
                     /label=estra_seq_reverse
                     /note="3008bp"
     misc_feature    9974..10371
                     /label=tGMP1_Termin
     primer_bind     complement(10467..10494)
                     /label=RPR1_TETR_Reverse_4
     gene            10495..11851
                     /label=kanMX
                     /note="yeast selectable marker conferring kanamycin
                     resistance (Wach et al., 1994)"
     promoter        10495..10838
                     /label=TEF promoter
                     /note="Ashbya gossypii TEF promoter"
     CDS             10839..11648
                     /codon_start=1
                     /gene="aph(3')-Ia"
                     /product="aminoglycoside phosphotransferase"
                     /label=KanR
                     /note="confers resistance to kanamycin"

/translation="MGKEKTHVSRPRLNSNMDADLYGYKWARDNVGQSGATIYRLYGKP

DAPELFLKHGKGSVANDVTDEMVRLNWLTEFMPLPTIKHFIRTPDDAWLLTTAIPGKTA

FQVLEEYPDSGENIVDALAVFLRRLHSIPVCNCPFNSDRVFRLAQAQSRMNNGLVDASD

FDDERNGWPVEQVWKEMHKLLPFSPDSVVTHGDFSLDNLIFDEGKLIGCIDVGRVGIAD
                     RYQDLAILWNCLGEFSPSLQKRLFQKYGIDNPDMNKLQFHLMLDEFF"
     terminator      11654..11851
                     /label=TEF terminator
```

```
                     /note="Ashbya gossypii TEF terminator"
     primer_bind     11852..11901
                     /label=HO_NOTI_For
     misc_feature    complement(11860..11878)
                     /label=T7 promoter
     primer_bind     complement(11860..11878)
                     /label=NAT_Reverse_5
                     /note="its been butched.
                     gaacttcgggcacagaatactttaatacgactcactatagggagac"
     misc_feature    11915..12414
                     /label=HO_End
                     /note="This will go downstream of the URA3 site and
remove
                     it
                     to insert the plasmid cut with NCOI with eitehr PMEI
or
                     FSEI. Or just use FSEI and PEMI"
     primer_bind     complement(12399..12446)
                     /label=HO_NCOI_Rev
     primer_bind     complement(12430..12446)
                     /label=End_Reverse_check
                     /note="To check sequence"
ORIGIN
        1 gcttcactgg ccgtcgtttt acaacgtcgt gactgggaaa accctggcgt
tacccaactt
       61 aatcgccttg cagcacatcc ccctttcgcc agctggcgta atagcgaaga
ggcccgcacc
      121 gatcgccctt cccaacagtt gcgcagcctg aatggcgaat ggacgcgccc
tgtagcggcg
      181 cattaagcgc ggcgggtgtg gtggttacgc gcagcgtgac cgctacactt
gccagcgccc
      241 tagcgcccgc tcctttcgct ttcttccctt cctttctcgc cacgttcgcc
ggctttcccc
      301 gtcaagctct aaatcggggg ctccctttag ggttccgatt tagtgcttta
cggcacctcg
      361 accccaaaaa acttgattag ggtgatggtt cacgtagtgg gccatcgccc
tgatagacgg
      421 tttttcgccc tttgacgttg gagtccacgt tctttaatag tggactcttg
ttccaaactg
      481 gaacaacact caaccctatc tcggtctatt cttttgattt ataagggatt
ttgccgattt
      541 cggcctattg gttaaaaaat gagctgattt aacaaaaatt taacgcgaat
tttaacaaaa
      601 tattaacgct tacaatttcc tgatgcggta ttttctcctt acgcatctgt
gcggtatttc
      661 acaccgcata gggtaataac tgatataatt aaattgaagc tctaatttgt
gagtttagta
      721 ttaggatctc taccettggc gaaaagtcct ctgccaacaa tgatgatatc
tgatccacca
      781 cttacaactt cgtcgacggt tctgtactgc tgacccaata tgcgtatata
taccaatcta
      841 agtctgtgct ccttccttcg ttcttccttc tgttcggaga ttaccgaatc
aaaaaaattt
```

901 caaggaaacc gaaatcaaaa aaaagaataa aaaaaaaatg atgaattgaa aaggtggtat
961 ggtgcactct cagtacaatc tgctctgatg ccgcatagtt aagccagccc cgacacccgc
1021 caacacccgc tgacgcgccc tgacgggctt gtctgctccc ggcatccgct tacagacaag
1081 ctgtgaccgt ctccgggagc tgcatgtgtc agaggttttc accgtcatca ccgaaacgcg
1141 cgagacgaaa gggcctcgtg atacgcctat ttttataggt taatgtcatg ataataatgg
1201 tttcttagac gtcggcgcgc cgaggacgtc aggtggcact tttcggggaa atgtgcgcgg
1261 aacccctatt tgtttatttt tctaaataca ttcaaatatg tatccgctca tgagacaata
1321 accctgataa atgcttcaat aatattgaaa aaggaagagt atgagtattc aacatttccg
1381 tgtcgccctt attccctttt ttgcggcatt ttgccttcct gtttttgctc acccagaaac
1441 gctggtgaaa gtaaaagatg ctgaagatca gttgggtgca cgagtgggtt acatcgaact
1501 ggatctcaac agcggtaaga tccttgagag ttttcgcccc gaagaacgtt ttccaatgat
1561 gagcactttt aaagttctgc tatgtggcgc ggtattatcc cgtattgacg ccgggcaaga
1621 gcaactcggt cgccgcatac actattctca gaatgacttg gttgagtact caccagtcac
1681 agaaaagcat cttacggatg gcatgacagt aagagaatta tgcagtgctg ccataaccat
1741 gagtgataac actgcggcca acttacttct gacaacgatc ggaggaccga aggagctaac
1801 cgcttttttg cacaacatgg gggatcatgt aactcgcctt gatcgttggg aaccggagct
1861 gaatgaagcc ataccaaacg acgagcgtga caccacgatg cctgtagcaa tggcaacaac
1921 gttgcgcaaa ctattaactg gcgaactact tactctagct tcccggcaac aattaataga
1981 ctggatggag gcggataaag ttgcaggacc acttctgcgc tcggcccttc cggctggctg
2041 gtttattgct gataaatctg gagccggtga gcgtgggtct cgcggtatca ttgcagcact
2101 ggggccagat ggtaagccct cccgtatcgt agttatctac acgacgggga gtcaggcaac
2161 tatggatgaa cgaaatagac agatcgctga gataggtgcc tcactgatta agcattggta
2221 actgtcagac caagtttact catatatact ttagattgat ttaaaacttc atttttaatt
2281 taaaaggatc taggtgaaga tcctttttga taatctcatg accaaaatcc cttaacgtga
2341 gttttcgttc cactgagcgt cagaccccgt agaaaagatc aaaggatctt cttgagatcc
2401 tttttttctg cgcgtaatct gctgcttgca aacaaaaaaa ccaccgctac cagcggtggt
2461 ttgtttgccg gatcaagagc taccaactct ttttccgaag gtaactggct tcagcagagc

2521 gcagatacca aatactgttc ttctagtgta gccgtagtta ggccaccact tcaagaactc
2581 tgtagcaccg cctacatacc tcgctctgct aatcctgtta ccagtggctg ctgccagtgg
2641 cgataagtcg tgtcttaccg ggttggactc aagacgatag ttaccggata aggcgcagcg
2701 gtcgggctga acggggggtt cgtgcacaca gcccagcttg gagcgaacga cctacaccga
2761 actgagatac ctacagcgtg agctatgaga aagcgccacg cttcccgaag ggagaaaggc
2821 ggacaggtat ccggtaagcg gcagggtcgg aacaggagag cgcacgaggg agcttccagg
2881 gggaaacgcc tggtatcttt atagtcctgt cgggtttcgc cacctctgac ttgagcgtcg
2941 atttttgtga tgctcgtcag gggggcggag cctatggaaa aacgccagca acgcggcctt
3001 tttacggttc ctggcctttt gctggccttt tgctcacatg ttctttcctg cgttatcccc
3061 tgattctgtg gataaccgta ttaccgcctt tgagtgagct gataccgctc gccgcagccg
3121 aacgaccgag cgcagcgagt cagtgagcga ggaagcggaa gagcgcccaa tacgcaaacc
3181 gcctctcccc gcgcgttggc cgattcatta atgcagctgg cacgacaggt ttcccgactg
3241 gaaagcgggc agtgagcgca acgcaattaa tgtgagttag ctcactcatt aggcacccca
3301 ggctttacac tttatgcttc cggctcctat gttgtgtgga attgtgagcg gataacaatt
3361 tcacacagga aacagctatg accatgatta cgccaagctc ggaattaacc ctcactaaag
3421 ggaacaaaag ctgggccggc catttaaatg tttaaacgct aaattcgagt gaaacacagg
3481 aagatcagaa aatcctcatt tcatccatat taacaataat ttcaaatgtt tatttgcatt
3541 atttgaaact aggcaagaca agcaacgaaa cgtttttgaa aattttgagt attttcaata
3601 aatttgtaga ggactcagat attgaaaaaa agctacagca attaatactt gataagaaga
3661 gtattgagaa gggcaacggt tcatcatctc atggatctgc acatgaacaa acaccagagt
3721 caaacgacgt tgaaattgag gctactgcgc caattgatga caatacagac gatgataaca
3781 aaccgaagtt atctgatgta gaaaaggatt aaagatgcta agagatagtg atgatatttc
3841 ataaataatg taattctata tatgttaatt accttttttg cgaggcatat ttatggtgaa
3901 ggataagttt tgaccatcaa agaaggttaa tgtggctgtg gtttcagggt ccataaagcg
3961 ctagcagtgc ttttaactaa gaattattag tcttttctgc ttattttttc atcatagttt
4021 agaacacttt atattaacga atagtttatg aatctattta ggtttaaaaa ttgatacagt
4081 tttataagtt actttttcaa agactcgtgc tgtctattgc ataatgcact ggaaggggaa

4141 aaaaaaggtg cacacgcgtg gctttttctt gaatttgcag tttgaaaaat gctgccaaac
4201 cagatgtcaa cacagctaca acgttatatt gaattttcaa aaattcttac tttttttttg
4261 gatggacgca aagaagttta ataatcatat tacatggcat taccaccata tacatatcca
4321 tatacatatc catatctaat cttacttata tgttgtggaa atgtaaagag ccccattatc
4381 ttagcctaaa aaaaccttct ctttggaact ttcagtaata cgcttaactg ctcattgcta
4441 tattgaagtg tggccgcggc ggaggagtgc ggcggaggag gagcggcgga ggagtgcggc
4501 ggaggaggag cggcggagga gtgcggcgga ggagtctaga aattgcgtcc tcgtcttcac
4561 cggtcgcgtt cctgaaacgc agatgtgcct aatgccgcac tgctccgaac aataaagatt
4621 ctacaatact agcttttatg gttatgaaga ggaaaaattg gcagtaacct ggccccacaa
4681 accttcaaat taacgaatca aattaacaac cataggatga taatgcgatt agttttttag
4741 ccttatttct ggggtaatta atcagcgaag cgatgatttt tgatctatta acagatatat
4801 aaatggaaaa gctgcataac cactttaact aatactttca acattttcag tttgtattac
4861 ttcttattca aatgtcataa aagtatcaac aaaaaattgt taatatacct ctatacttta
4921 acgtcaagga gaaaaaacta taagatccac gtgagctgcc ctaggatgga acgactacag
4981 agattgatga tgaatagtaa ggtggggtcc gcggacaccg gccgtgacga tacgaaagaa
5041 actgtttata tttcttcgat tgcgctttta aagatgctaa agcatggtag agctggtgtt
5101 cccatggaag tcatggggtt gatgttaggt gagtttgtcg atgattatac ggttaacgtt
5161 gtggacgtgt ttgcgatgcc tcaatcgggt accggagttt ctgttgaggc tgtcgatgat
5221 gttttccaag cgaagatgat ggacatgtta aaacaaacgg gcagagacca aatggtcgtt
5281 ggctggtacc actctcatcc agggtttggc tgttggctat cttctgttga tgttaatact
5341 caaaaatctt ttgaacaact aaacagcaga gctgttgctg tcgttgttga ccctattcaa
5401 tccgttaagg gaaaagttgt catcgatgct tttagattga ttgacacggg cgcattgata
5461 aataacttag agcctagaca aacaacctcc aacacaggct tattgaacaa ggccaacatt
5521 caagccttaa ttcacggtct gaataggcat tactattctt taaatattga ttatcataaa
5581 accgcgaagg aaaccaagat gttaatgaac ttacataaag aacagtggca atcaggtctt
5641 aagatgtacg attatgaaga aaaagaagaa tcaaatttgg ctgctacaaa gagtatggtt
5701 aagatagccg aacagtactc taagagaata gaagaggaaa aggaattaac cgaagaagaa

5761 cttaagacaa gatacgttgg taggcaagat ccaaagaagc acctttccga
aacagcagat
5821 gagacactag agaacaatat tgtttctgtg ctgacggcgg gtgttaattc
agtggcaatt
5881 aaagatccgc tagcgctacc ggtcgccacc atggtgagca agggcgagga
ggataacatg
5941 gccatcatca aggagttcat gcgcttcaag gtgcacatgg agggctccgt
gaacggccac
6001 gagttcgaga tcgagggcga gggcgagggc cgcccctacg agggcaccca
gaccgccaag
6061 ctgaaggtga ccaagggtgg cccccctgccc ttcgcctggg acatcctgtc
ccctcagttc
6121 atgtacggct ccaaggccta cgtgaagcac cccgccgaca tccccgacta
cttgaagctg
6181 tccttccccg agggcttcaa gtgggagcgc gtgatgaact tcgaggacgg
cggcgtggtg
6241 accgtgaccc aggactcctc cttgcaggat ggcgagttca tctacaaggt
gaagctgcgc
6301 ggcaccaact tcccctccga cggccccgta atgcagaaga agaccatggg
ctgggaggcc
6361 tcctccgagc ggatgtaccc cgaggacggc gccctgaagg gcgagatcaa
gcagaggctg
6421 aagctgaagg acggcggcca ctacgacgct gaggtcaaga ccacctacaa
ggccaagaag
6481 cccgtgcagc tgcccggcgc ctacaacgtc aacatcaagt tggacatcac
ctcccacaac
6541 gaggactaca ccatcgtgga acagtacgaa cgcgccgagg gccgccactc
caccggcggc
6601 atggacgagc tgtacaagtc cggactcaga tcttacgatt taggtgacac
tatagaacgc
6661 ggccgccagg actacaagga ccatgacggt gattacaagg atcatgacat
cgactacaag
6721 gatgacgatg acaagcatca tcaccatcac catggttgag gcgcgccact
tctaaataag
6781 cgaatttctt atgatttatg atttttatta ttaaataagt tataaaaaaa
ataagtgtat
6841 acaaatttta aagtgactct taggttttaa aacgaaaatt cttattcttg
agtaactctt
6901 tcctgtaggt caggttgctt tctcaggtat agtatgaggt cgctcttatt
gaccacacct
6961 ctaccggcag atcccctgca ggacgatggc ttcttatctc acttcaatag
tactttccac
7021 cggttatact tccggctttt ccctattaat acaagctaca atttcaatgg
gtggcaaata
7081 atgtgtagaa tagaaaataa gccgacaggg taataaagaa aatttttaga
aaaaaaaggt
7141 tagatggctt atttaagtta caggctagcg aaaaaaggaa cttcagggca
agtaaagtgt
7201 ttgattgggc actagcatgg cttataaagg cgagcaattg tcgaaactaa
ttaatgttgt
7261 acggactatt gctgtcatct cgtggtaaat gcgtgttcca ggtcgaatac
tacttgcaca
7321 caggcgagcg gggccccata aaagtgttgc cgatttgtta agttgtcttt
tcggtttttc

7381 tactctgtta ttccttactt cccttttttaa gaactctttt tatccttcat
ttaggatctt
7441 gcacgtttcc gcctcatcac ttgaattaaa acatgtctct gtcagtaaac
cttggcgttt
7501 ctattgttct tcatagttca acttttatta ttacccgccc tgcgcgttta
catttttcca
7561 gcaacagcca gcgaaaaatt agaaaatctg gttgttgaca cctcaagaac
aagggcaatt
7621 agcctcagcg tcgaatatag atcatattag aatacctata gctccatcaa
aagaaataca
7681 caagatctat gggtacccgc ccatatgctt gccctgtcga gtcctgcgat
cgccgctttt
7741 ctcgccacgc caatcttacc cgccatatcc gcatccatac cggtcagaag
cccttccagt
7801 gtcgaatctg catgcgtaac ttcagtcgta atgcgaacct tgtgcgccac
atccgcaccc
7861 acacaggatc ccaaaagccg ttccaatgtc ggatctgtat gcggaacttt
agtcgaaagg
7921 ccgacctgag gcgtcacatt cgcacgcaca ccggcgagaa gccttttgcc
tgtgacattt
7981 gtgggaggaa gtttgccagg aagggcgacc tcaagaggca taccaaaatc
catacaggta
8041 gatccggtga cggtgctggt ttaattaact ctgctggaga catgagagct
gccaaccttt
8101 ggccaagccc gctcatgatc aaacgctcta agaagaacag cctggccttg
tccctgacgg
8161 ccgaccagat ggtcagtgcc ttgttggatg ctgagccccc catactctat
tccgagtatg
8221 atcctaccag acccttcagt gaagcttcga tgatgggctt actgaccaac
ctggcagaca
8281 gggagctggt tcacatgatc aactgggcga agagggtgcc aggctttgtg
gatttgaccc
8341 tccatgatca ggtccacctt ctagaatgtg cctggctaga gatcctgatg
attggactcg
8401 tctggcgctc catggagcac ccagggaagc tactgtttgc tcctaacttg
ctcttggaca
8461 ggaaccaggg aaaatgtgta gagggcatgg tggagatctt cgacatgctg
ctggctacat
8521 catctcggtt ccgcatgatg aatctgcagg gagaggagtt tgtgtgcctc
aaatctatta
8581 ttttgcttaa ttctggagtg tacacatttc tgtccagcac cctgaagtct
ctggaagaga
8641 aggaccatat ccaccgagtc ctggacaaga tcacagacac tttgatccac
ctgatggcca
8701 aggcaggcct gaccctgcag cagcagcacc agcggctggc ccagctcctc
ctcatcctct
8761 cccacatcag gcacatgagt aacaaaggca tggagcatct gtacagcatg
aagtgcaaga
8821 acgtggtgcc cctctatgac ctgctgctgg agatgctgga cgcccaccgc
ctacatgcgc
8881 ccactagccg tggaggggca tccgtggagg aaacggacca aagccacttg
gccactgcgg
8941 gctctacttc atcgcattcc ttgcaaaagt attacatcac gggggaggca
gagggtttcc

9001 ctgccacagt cgcggctgca ggtgacggtg ctggtttaat taacatgacg gtcgaccatg
9061 atttcaatag cgaagatatt ttattcccca tagaaagcat gagtagtata caatacgtgg
9121 agaataataa cccaaataat attaacaacg atgttatccc gtattctcta gatatcaaaa
9181 acactgtctt agatagtgcg gatctcaatg acattcaaaa tcaagaaact tcactgaatt
9241 tggggcttcc tccactatct ttcgactctc cactgcccgt aacggaaacg ataccatcca
9301 ctaccgataa cagcttgcat ttgaaagctg atagcaacaa aaatcgcgat gcaagaacta
9361 ttgaaaatga tagtgaaatt aagagtacta ataatgctag tggctctggg gcaaatcaat
9421 acacaactct tacttcacct tatcctatga acgacatttt gtacaacatg aacaatccgt
9481 tacaatcacc gtcaccttca tcggtacctc aaaatccgac tataaatcct cccataaata
9541 cagcaagtaa cgaaactaat ttatcgcctc aaacttcaaa tggtaatgaa actcttatat
9601 ctcctcgagc ccaacaacat acgtccatta aagataatcg tctgtcctta cctaatggtg
9661 ctaattcgaa tcttttcatt gacactaacc caaacaattt gaacgaaaaa ctaagaaatc
9721 aattgaactc agatacaaat tcatattcta actccatttc taattcaaac tccaattcta
9781 cgggtaattt aaattccagt tattttaatt cactgaacat agactccatg ctagatgatt
9841 acgtttctag tgatctctta ttgaatgatg atgatgatga cactaattta tcacgccgaa
9901 gatttagcga cgttataaca aaccaatttc cgtcaatgac aaattcgagg aatgagctcg
9961 gatcctaact taagtctgaa gaatgaatga tttgatgatt tctttttccc tccatttttc
10021 ttactgaata tatcaatgat atagacttgt atagtttatt atttcaaatt aagtagctat
10081 atatagtcaa gataacgttt gtttgacacg attacattat tcgtcgacat ctttttttcag
10141 cctgtcgtgg tagcaatttg aggagtatta ttaattgaat aggttcattt tgcgctcgca
10201 taaacagttt tcgtcaggga cagtatgttg gaatgagtgg taattaatgg tgacatgaca
10261 tgttatagca ataaccttga tgtttacatc gtagtttaat gtacaccccg cgaattcgtt
10321 caagtaggag tgcaccaatt gcaaagggaa aagctgaatg ggcagttcga accgcggtct
10381 ttcctgcgtt atcccctgat tctgtggata accgtattac cgcctttgag tgagctgata
10441 ccgctcgccg cagccgaacg accgagcgca gcgagtcagt gagcgaggct gagcgacatg
10501 gaggcccaga ataccctcct tgacagtctt gacgtgcgca gctcaggggc atgatgtgac
10561 tgtcgcccgt acatttagcc catacatccc catgtataat catttgcatc catacatttt

10621 gatggccgca cggcgcgaag caaaaattac ggctcctcgc tgcagacctg cgagcaggga
10681 aacgctcccc tcacagacgc gttgaattgt ccccacgccg cgcccctgta gagaaatata
10741 aaaggttagg atttgccact gaggttcttc tttcatatac ttccttttaa aatcttgcta
10801 ggatacagtt ctcacatcac atccgaacat aaacaaccat gggtaaggaa aagactcacg
10861 tttcgaggcc gcgattaaat tccaacatgg atgctgattt atatgggtat aaatgggctc
10921 gcgataatgt cgggcaatca ggtgcgacaa tctatcgatt gtatgggaag cccgatgcgc
10981 cagagttgtt tctgaaacat ggcaaaggta gcgttgccaa tgatgttaca gatgagatgg
11041 tcagactaaa ctggctgacg gaatttatgc ctcttccgac catcaagcat tttatccgta
11101 ctcctgatga tgcatggtta ctcaccactg cgatccccgg caaaacagca ttccaggtat
11161 tagaagaata tcctgattca ggtgaaaata ttgttgatgc gctggcagtg ttcctgcgcc
11221 ggttgcattc gattcctgtt tgtaattgtc cttttaacag cgatcgcgta tttcgtctcg
11281 ctcaggcgca atcacgaatg aataacggtt tggttgatgc gagtgatttt gatgacgagc
11341 gtaatggctg gcctgttgaa caagtctgga aagaaatgca taagcttttg ccattctcac
11401 cggattcagt cgtcactcat ggtgatttct cacttgataa ccttattttt gacgagggga
11461 aattaatagg ttgtattgat gttggacgag tcggaatcgc agaccgatac caggatcttg
11521 ccatcctatg gaactgcctc ggtgagtttt ctccttcatt acagaaacgg ctttttcaaa
11581 aatatggtat tgataatcct gatatgaata aattgcagtt tcatttgatg ctcgatgagt
11641 ttttctaatc agtactgaca ataaaaagat tcttgttttc aagaacttgt catttgtata
11701 gtttttttat attgtagttg ttctatttta atcaaatgtt agcgtgattt atattttttt
11761 tcgcctcgac atcatctgcc cagatgcgaa gttaagtgcg cagaaagtaa tatcatgcgt
11821 caatcgtatg tgaatgctgg tcgctatact ggcggccgcc ctatagtgag tcgtattaga
11881 gttattaccc gagtagagca cttgaatcca ctgccccggg aatctcggtc gtaatgattt
11941 ctataatgac gaaaaaaaaa aaattggaaa gaaaaagctt catggccttt ataaaaagga
12001 actatccaat acctcgccag aaccaagtaa cagtatttta cggggcacaa atcaagaaca
12061 ataagacagg actgtaaaga tggacgcatt gaactccaaa gaacaacaag agttccaaaa
12121 agtagtggaa caaaagcaaa tgaaggattt catgcgtttg tactctaatc tggtagaaag
12181 atgtttcaca gactgtgtca atgacttcac aacatcaaag ctaaccaata aggaacaaac

12241 atgcatcatg aagtgctcag aaaagttctt gaagcatagc gaacgtgtag ggcagcgttt
12301 ccaagaacaa aacgctgcct tgggacaagg cttgggccga taaggtgtac tggcgtatat
12361 atatctaatt atgtatctct ggtgtagccc atttttagca tgtaaatata aagaccgagc
12421 gggccggcct cgggccatgg tcactg
//

Plasmid: Z4EM_Rpn4_Truncated for inducible *RPN4 (truncated) construct

```
LOCUS       Z4EM_Rpn4_Trunca       12412 bp    DNA     circular SYN 29-
NOV-2022
DEFINITION  synthetic circular DNA
ACCESSION   .
VERSION     .
KEYWORDS    .
SOURCE      synthetic DNA construct
  ORGANISM  recombinant plasmid
REFERENCE   1  (bases 1 to 12412)
  AUTHORS   Trial User
  TITLE     Direct Submission
  JOURNAL   Exported Dec 22, 2025 from SnapGene 8.2.1
            https://www.snapgene.com
FEATURES             Location/Qualifiers
     source          1..12412
                     /mol_type="other DNA"
                     /organism="recombinant plasmid"
     primer_bind     complement(6..22)
                     /label=M13 fwd
                     /note="common sequencing primer, one of multiple
similar
                     variants"
     primer_bind     6..22
                     /label=Leu2_NCoI_Forward_1
     rep_origin      163..618
                     /direction=RIGHT
                     /label=f1 ori
                     /note="f1 bacteriophage origin of replication; arrow
                     indicates direction of (+) strand synthesis"
     promoter        1256..1360
                     /gene="bla"
                     /label=AmpR promoter
     CDS             join(1361..1429,1430..2221)
                     /codon_start=1
                     /gene="bla"
                     /product="beta-lactamase"
                     /label=AmpR
                     /note="confers resistance to ampicillin,
carbenicillin, and
                     related antibiotics"

/translation="MSIQHFRVALIPFFAAFCLPVFAHPETLVKVKDAEDQLGARVGYI

ELDLNSGKILESFRPEERFPMMSTFKVLLCGAVLSRIDAGQEQLGRRIHYSQNDLVEYS

PVTEKHLTDGMTVRELCSAAITMSDNTAANLLLTTIGGPKELTAFLHNMGDHVTRLDRW

EPELNEAIPNDERDTTMPVAMATTLRKLLTGELLTLASRQQLIDWMEADKVAGPLLRSA

LPAGWFIADKSGAGERGSRGIIAALGPDGKPSRIVVIYTTGSQATMDERNRQIAEIGAS
                     LIKHW"
     rep_origin      2392..2980
```

```
                     /direction=RIGHT
                     /label=ori
                     /note="high-copy-number ColE1/pMB1/pBR322/pUC origin
of
                     replication"
     primer_bind     complement(3131..3152)
                     /label=RPR1_TETR_Reverse_4
     protein_bind    3268..3289
                     /label=CAP binding site
                     /bound_moiety="E. coli catabolite activator protein"
                     /note="CAP binding activates transcription in the
presence
                     of cAMP."
     promoter        join(3304..3309,3310..3327,3328..3334)
                     /label=lac promoter
                     /note="promoter for the E. coli lac operon"
     protein_bind    3342..3358
                     /label=lac operator
                     /bound_moiety="lac repressor encoded by lacI"
                     /note="The lac repressor binds to the lac operator
to
                     inhibit transcription in E. coli. This inhibition
can be
                     relieved by adding lactose or
                     isopropyl-beta-D-thiogalactopyranoside (IPTG)."
     primer_bind     3366..3382
                     /label=M13 rev
                     /note="common sequencing primer, one of multiple
similar
                     variants"
     primer_bind     3386..3402
                     /label=TOP_Check_Forward
     promoter        3403..3421
                     /label=T3 promoter
                     /note="promoter for bacteriophage T3 RNA polymerase"
     primer_bind     3429..3479
                     /label=HO_FSEI_Forward
     misc_feature    3458..3957

                     /note="URA3 forward for homology site cut with PMEI,
FSEI,
                     with NCOI "
     primer_bind     complement(3939..3966)
                     /label=NHEI_HO_Rever
     primer_bind     3956..3992
                     /label=NHEI_prestra_For
     misc_feature    3966..4190
                     /locus_tag="Sc tENO2"
                     /label=Sc tENO2
     primer_bind     4188..4208
                     /label=prestra_seq_for
                     /note="sequencing 2044bp"
     misc_feature    4195..4219
                     /locus_tag="Con1 scar"
```

```
                     /label=Con1 scar
     promoter        4224..4907
                     /locus_tag="pZ4 (-Gal4 site)"
                     /label=pZ4 (-Gal4 site)
     misc_feature    4456..4467
                     /locus_tag="Z4 Binding Site(2)"
                     /label=Z4 Binding Site
                     /label=Z4 Binding Site(2)
                     /label=nonstandard type: Binding
     misc_feature    4469..4480
                     /locus_tag="Z4 Binding Site(1)"
                     /label=Z4 Binding Site
                     /label=Z4 Binding Site(1)
                     /label=nonstandard type: Binding
     misc_feature    4483..4494
                     /locus_tag="Z4 Binding Site(3)"
                     /label=Z4 Binding Site
                     /label=Z4 Binding Site(3)
                     /label=nonstandard type: Binding
     misc_feature    4496..4507
                     /locus_tag="Z4 Binding Site(4)"
                     /label=Z4 Binding Site
                     /label=Z4 Binding Site(4)
                     /label=nonstandard type: Binding
     misc_feature    4510..4521
                     /locus_tag="Z4 Binding Site(5)"
                     /label=Z4 Binding Site
                     /label=Z4 Binding Site(5)
                     /label=nonstandard type: Binding
     misc_feature    4523..4534
                     /locus_tag="Z4 Binding Site"
                     /label=Z4 Binding Site
                     /label=nonstandard type: Binding
     misc_feature    complement(4547..4550)
                     /locus_tag="Bbs1(6)"
                     /label=Bbs1
                     /label=Bbs1(6)
     misc_feature    complement(4553..4558)
                     /locus_tag="Bbs1(4)"
                     /label=Bbs1
                     /label=Bbs1(4)
     promoter        4908..4920
                     /locus_tag="pZ4 (-Gal4 site)(1)"
                     /label=pZ4 (-Gal4 site)
                     /label=pZ4 (-Gal4 site)(1)
     promoter        4921..4947
                     /locus_tag="pZ4 (-Gal4 site)(2)"
                     /label=pZ4 (-Gal4 site)
                     /label=pZ4 (-Gal4 site)(2)
     primer_bind     complement(4922..4965)
                     /label=BSu36I_pestra_Rev
     misc_feature    4948..4960
                     /label=Sequence_insert
     misc_feature    4954..4959
```

```
                     /label=Sequencing_insert
     primer_bind     4957..4999
                     /label=Rpn4_Trun_AvrII_For
     misc_feature    4966..4971
                     /label=Kozak
     CDS             4972..4974
                     /codon_start=1
                     /label=Rpn4
                     /translation="M"
     CDS             4975..6483
                     /codon_start=1

/product="https://journals.plos.org/plosone/article?id=10.1
                     371/journal.pone.0009877#pone-0009877-t001"
                     /label=Rpn4

/translation="TLTDILEDELYHTNPGHSQFTSHYQNYHPNASITPYKLVNKNKEN

NTFTWNHSLQHQNESSAASIPPQQTYHFPIFNKYADPTLTTTTSFTTSEATANDRQINN

VHLIPNEIKGASETPLQKTVNLKNIMKVSDPYVPTRNTFNYDVKISNDFFDNGDNLYGN

DEEVLFYEDNYNPKMQWSLQDNSAAINNEDARAIFNNNCLQQEQHQEEPLLSLDVTPIS

MFGSDQKTGRAKSSSHLFNEYSYVDSNMDSISSVVSEDLLDERGHEKIEDEDEDNDLDE

DDIYDISLLKNRRKQSFVLNKNTIDFERFPSPSTSANVPSTATTGKRKPAKSSSNRSCV

SNSNENGTLERIKKPTSAVVSSNASRRKLINYTKKHLSSHSSTNSNSKPSTASPSAHTS

SSDGNNEIFTCQIMNLITNEPCGAQFSRSYDLTRHQNTIHAKRKIVFRCSECIKILGSE
                     GYQKTFSRLDALTRHIKSKHEDLSLEQRQEVTKFAKANIGYVMG"
     misc_feature    4975..4977
                     /label=Truncated
                     /note="ATGGCTTCTACGGAACTTAGCCTAAAAAGA

                     Above is the truncated sequence; however, with the
kozak I
                     added back methione

https://journals.plos.org/plosone/article?id=10.1371/journa
                     l.pone.0009877#pone-0009877-t001

                     RPN4-Delta-1,Äì10
                     "
     primer_bind     5139..5158
                     /label=Rpn4_for_seq
     misc_feature    5575..5577
                     /label=Truncated

/note="GAATTTGACTCTGATGACGACGATATCAGTGATGATGAAGAGGATGAAATAG
                     AAGAA

                     Sequence above was removed
```

```
                     RPN4-Delta-1,Äì10/Delta-211,Äì229

https://journals.plos.org/plosone/article?id=10.1371/journa
                     l.pone.0009877#pone-0009877-t001"
     primer_bind     complement(6919..6948)
                     /label=Rpn4_reverse_trunc
     primer_bind     6941..6969
                     /label=SBFI_Estro_For
     misc_feature    6947..6948
                     /locus_tag="Con1 scar"
                     /label=Con1 scar
     misc_feature    6949..7648
                     /locus_tag="Sc pRET2"
                     /label=Sc pRET2
     misc_feature    7655..8005
                     /locus_tag="pZ4 DNA Binding Domain"
                     /label=pZ4 DNA Binding Domain
                     /label=nonstandard type:  ORF
     misc_feature    8036..8977
                     /locus_tag="hER-LBD"
                     /label=hER-LBD
     misc_feature    9011..9934
                     /locus_tag="Msn2 Activation Domain (27 more nt's,
encoding
                     FPSMTNSRN)"
                     /label=Msn2 Activation Domain (27 more nt's,
                     encoding ...
                     /label=Msn2 Activation Domain (27 more nt's,
encoding
                     FPSMTNSRN)
                     /label=nonstandard type:  ORF
     primer_bind     complement(9915..9946)
                     /label=AFIII_Estro_RE_Rev
     primer_bind     complement(9915..9935)
                     /label=estra_seq_reverse
                     /note="3008bp"
     misc_feature    9940..10337
                     /label=tGMP1_Termin
     primer_bind     complement(10433..10460)
                     /label=RPR1_TETR_Reverse_4
     gene            10461..11817
                     /label=kanMX
                     /note="yeast selectable marker conferring kanamycin
                     resistance (Wach et al., 1994)"
     promoter        10461..10804
                     /label=TEF promoter
                     /note="Ashbya gossypii TEF promoter"
     CDS             10805..11614
                     /codon_start=1
                     /gene="aph(3')-Ia"
                     /product="aminoglycoside phosphotransferase"
                     /label=KanR
                     /note="confers resistance to kanamycin"
```

```
/translation="MGKEKTHVSRPRLNSNMDADLYGYKWARDNVGQSGATIYRLYGKP

DAPELFLKHGKGSVANDVTDEMVRLNWLTEFMPLPTIKHFIRTPDDAWLLTTAIPGKTA

FQVLEEYPDSGENIVDALAVFLRRLHSIPVCNCPFNSDRVFRLAQAQSRMNNGLVDASD

FDDERNGWPVEQVWKEMHKLLPFSPDSVVTHGDFSLDNLIFDEGKLIGCIDVGRVGIAD
                     RYQDLAILWNCLGEFSPSLQKRLFQKYGIDNPDMNKLQFHLMLDEFF"
     terminator      11620..11817
                     /label=TEF terminator
                     /note="Ashbya gossypii TEF terminator"
     primer_bind     11818..11867
                     /label=HO_NOTI_For
     misc_feature    complement(11826..11844)
                     /label=T7 promoter
     primer_bind     complement(11826..11844)
                     /label=NAT_Reverse_5
                     /note="its been butched.
                     gaacttcgggcacagaatactttaatacgactcactatagggagac"
     misc_feature    11881..12380
                     /label=HO_End
                     /note="This will go downstream of the URA3 site and
remove
                     it
                     to insert the plasmid cut with NCOI with eitehr PMEI
or
                     FSEI. Or just use FSEI and PEMI"
     primer_bind     complement(12365..12412)
                     /label=HO_NCOI_Rev
     primer_bind     complement(12396..12412)
                     /label=End_Reverse_check
                     /note="To check sequence"
ORIGIN
        1 gcttcactgg ccgtcgtttt acaacgtcgt gactgggaaa accctggcgt
tacccaactt
       61 aatcgccttg cagcacatcc ccctttcgcc agctggcgta atagcgaaga
ggcccgcacc
      121 gatcgccctt cccaacagtt gcgcagcctg aatggcgaat ggacgcgccc
tgtagcggcg
      181 cattaagcgc ggcgggtgtg gtggttacgc gcagcgtgac cgctacactt
gccagcgccc
      241 tagcgcccgc tcctttcgct ttcttccctt cctttctcgc cacgttcgcc
ggctttcccc
      301 gtcaagctct aaatcggggg ctccctttag ggttccgatt tagtgcttta
cggcacctcg
      361 accccaaaaa acttgattag ggtgatggtt cacgtagtgg gccatcgccc
tgatagacgg
      421 tttttcgccc tttgacgttg gagtccacgt tctttaatag tggactcttg
ttccaaactg
      481 gaacaacact caaccctatc tcggtctatt cttttgattt ataagggatt
ttgccgattt
      541 cggcctattg gttaaaaaat gagctgattt aacaaaaatt taacgcgaat
tttaacaaaa
```

601 tattaacgct tacaatttcc tgatgcggta ttttctcctt acgcatctgt gcggtatttc
661 acaccgcata gggtaataac tgatataatt aaattgaagc tctaatttgt gagtttagta
721 ttaggatctc taccccttggc gaaaagtcct ctgccaacaa tgatgatatc tgatccacca
781 cttacaactt cgtcgacggt tctgtactgc tgacccaata tgcgtatata taccaatcta
841 agtctgtgct ccttccttcg ttcttccttc tgttcggaga ttaccgaatc aaaaaaattt
901 caaggaaacc gaaatcaaaa aaaagaataa aaaaaaaatg atgaattgaa aaggtggtat
961 ggtgcactct cagtacaatc tgctctgatg ccgcatagtt aagccagccc cgacacccgc
1021 caacacccgc tgacgcgccc tgacgggctt gtctgctccc ggcatccgct tacagacaag
1081 ctgtgaccgt ctccgggagc tgcatgtgtc agaggttttc accgtcatca ccgaaacgcg
1141 cgagacgaaa gggcctcgtg atacgcctat ttttataggt taatgtcatg ataataatgg
1201 tttcttagac gtcggcgcgc cgaggacgtc aggtggcact tttcggggaa atgtgcgcgg
1261 aacccctatt tgtttatttt tctaaataca ttcaaatatg tatccgctca tgagacaata
1321 accctgataa atgcttcaat aatattgaaa aaggaagagt atgagtattc aacatttccg
1381 tgtcgccctt attccctttt ttgcggcatt ttgccttcct gtttttgctc acccagaaac
1441 gctggtgaaa gtaaaagatg ctgaagatca gttgggtgca cgagtgggtt acatcgaact
1501 ggatctcaac agcggtaaga tccttgagag ttttcgcccc gaagaacgtt ttccaatgat
1561 gagcactttt aaagttctgc tatgtggcgc ggtattatcc cgtattgacg ccgggcaaga
1621 gcaactcggt cgccgcatac actattctca gaatgacttg gttgagtact caccagtcac
1681 agaaaagcat cttacggatg gcatgacagt aagagaatta tgcagtgctg ccataaccat
1741 gagtgataac actgcggcca acttacttct gacaacgatc ggaggaccga aggagctaac
1801 cgcttttttg cacaacatgg gggatcatgt aactcgcctt gatcgttggg aaccggagct
1861 gaatgaagcc ataccaaacg acgagcgtga caccacgatg cctgtagcaa tggcaacaac
1921 gttgcgcaaa ctattaactg gcgaactact tactctagct tcccggcaac aattaataga
1981 ctggatggag gcggataaag ttgcaggacc acttctgcgc tcggcccttc cggctggctg
2041 gtttattgct gataaatctg gagccggtga gcgtgggtct cgcggtatca ttgcagcact
2101 ggggccagat ggtaagccct cccgtatcgt agttatctac acgacgggga gtcaggcaac
2161 tatggatgaa cgaaatagac agatcgctga gataggtgcc tcactgatta agcattggta

2221 actgtcagac caagtttact catatatact ttagattgat ttaaaacttc atttttaatt
2281 taaaaggatc taggtgaaga tcctttttga taatctcatg accaaaatcc cttaacgtga
2341 gttttcgttc cactgagcgt cagaccccgt agaaaagatc aaaggatctt cttgagatcc
2401 tttttttctg cgcgtaatct gctgcttgca aacaaaaaaa ccaccgctac cagcggtggt
2461 ttgtttgccg gatcaagagc taccaactct ttttccgaag gtaactggct tcagcagagc
2521 gcagatacca aatactgttc ttctagtgta gccgtagtta ggccaccact tcaagaactc
2581 tgtagcaccg cctacatacc tcgctctgct aatcctgtta ccagtggctg ctgccagtgg
2641 cgataagtcg tgtcttaccg ggttggactc aagacgatag ttaccggata aggcgcagcg
2701 gtcgggctga acggggggtt cgtgcacaca gcccagcttg gagcgaacga cctacaccga
2761 actgagatac ctacagcgtg agctatgaga aagcgccacg cttcccgaag ggagaaaggc
2821 ggacaggtat ccggtaagcg gcagggtcgg aacaggagag cgcacgaggg agcttccagg
2881 gggaaacgcc tggtatcttt atagtcctgt cgggtttcgc cacctctgac ttgagcgtcg
2941 atttttgtga tgctcgtcag gggggcggag cctatggaaa aacgccagca acgcggcctt
3001 tttacggttc ctggcctttt gctggccttt tgctcacatg ttctttcctg cgttatcccc
3061 tgattctgtg gataaccgta ttaccgcctt tgagtgagct gataccgctc gccgcagccg
3121 aacgaccgag cgcagcgagt cagtgagcga ggaagcggaa gagcgcccaa tacgcaaacc
3181 gcctctcccc gcgcgttggc cgattcatta atgcagctgg cacgacaggt ttcccgactg
3241 gaaagcgggc agtgagcgca acgcaattaa tgtgagttag ctcactcatt aggcacccca
3301 ggctttacac tttatgcttc cggctcctat gttgtgtgga attgtgagcg gataacaatt
3361 tcacacagga aacagctatg accatgatta cgccaagctc ggaattaacc ctcactaaag
3421 ggaacaaaag ctgggccggc catttaaatg tttaaacgct aaattcgagt gaaacacagg
3481 aagatcagaa aatcctcatt tcatccatat taacaataat ttcaaatgtt tatttgcatt
3541 atttgaaact aggcaagaca agcaacgaaa cgtttttgaa aattttgagt attttcaata
3601 aatttgtaga ggactcagat attgaaaaaa agctacagca attaatactt gataagaaga
3661 gtattgagaa gggcaacggt tcatcatctc atggatctgc acatgaacaa acaccagagt
3721 caaacgacgt tgaaattgag gctactgcgc caattgatga caatacagac gatgataaca
3781 aaccgaagtt atctgatgta gaaaaggatt aaagatgcta agagatagtg atgatatttc

3841 ataaataatg taattctata tatgttaatt accttttttg cgaggcatat ttatggtgaa
3901 ggataagttt tgaccatcaa agaaggttaa tgtggctgtg gtttcagggt ccataaagcg
3961 ctagcagtgc ttttaactaa gaattattag tcttttctgc ttattttttc atcatagttt
4021 agaacacttt atattaacga atagtttatg aatctattta ggtttaaaaa ttgatacagt
4081 tttataagtt actttttcaa agactcgtgc tgtctattgc ataatgcact ggaaggggaa
4141 aaaaaaggtg cacacgcgtg gctttttctt gaatttgcag tttgaaaaat gctgccaaac
4201 cagatgtcaa cacagctaca acgttatatt gaattttcaa aaattcttac tttttttttg
4261 gatggacgca aagaagttta ataatcatat tacatggcat taccaccata tacatatcca
4321 tatacatatc catatctaat cttacttata tgttgtggaa atgtaaagag ccccattatc
4381 ttagcctaaa aaaaccttct ctttggaact ttcagtaata cgcttaactg ctcattgcta
4441 tattgaagtg tggccgcggc ggaggagtgc ggcggaggag gagcggcgga ggagtgcggc
4501 ggaggaggag cggcggagga gtgcggcgga ggagtctaga aattgcgtcc tcgtcttcac
4561 cggtcgcgtt cctgaaacgc agatgtgcct aatgccgcac tgctccgaac aataaagatt
4621 ctacaatact agcttttatg gttatgaaga ggaaaaattg gcagtaacct ggccccacaa
4681 accttcaaat taacgaatca aattaacaac cataggatga taatgcgatt agtttttttag
4741 ccttatttct ggggtaatta atcagcgaag cgatgatttt tgatctatta acagatatat
4801 aaatggaaaa gctgcataac cactttaact aatactttca acattttcag tttgtattac
4861 ttcttattca aatgtcataa aagtatcaac aaaaaattgt taatatacct ctatacttta
4921 acgtcaagga gaaaaaacta taagatccac gtgagctgcc ctagggccac catgacctta
4981 acggatattt tagaagacga gttgtaccat actaatccag gtcacagtca gtttacgagt
5041 cattatcaaa actatcatcc aaatgctagt attactccat ataagttggt gaataagaac
5101 aaggaaaaca acactttttac gtggaatcat tcattacaac accagaatga atcgagtgca
5161 gcttcgatac ccccacaaca aacctaccat ttcccgatat tcaacaaata cgcggatcct
5221 actttaacta ccaccacctc ttttacgact agtgaagcaa cggccaacga tagacagatt
5281 aataatgtcc atctcatacc aaacgagatt aagggtgcta gcgaaacccc attgcagaag
5341 accgtcaatc taaagaatat aatgaaagta tcagacccgt atgtaccgac acggaatacg
5401 ttcaattatg atgttaaaat ttccaacgat tttttcgata acggtgacaa tctatatggt

5461 aatgatgaag aagtgctttt ctatgaggat aattataatc cgaaaatgca gtggtcactt
5521 caagataata gcgccgcaat aaacaatgag gatgcgagag ctatttttaa caataattgt
5581 ttgcaacaag agcaacacca agaggagcct ttactgtcat tggatgttac accaatctca
5641 atgtttggct cagatcaaaa aacgggtcgt gccaagagtt ctagtcattt atttaatgag
5701 tacagttacg ttgactctaa catggacagc atttccagtg ttgtatctga agatctgtta
5761 gatgaacggg gacatgagaa gatagaggat gaggatgagg ataatgatct tgatgaagac
5821 gatatctacg atatctctct cttgaagaac agaagaaagc aaagttttgt cctcaataaa
5881 aacactattg attttgaaag atttccatct ccctcaacct cggcaaacgt accgtctact
5941 gctactaccg gtaaaaggaa accagcaaaa tcatccagta accgtagttg cgttagtaac
6001 agtaatgaaa acggcacatt agaaagaata aagaagccta catcagctgt agtaagctca
6061 aatgctagta ggcggaagct aattaattat actaagaagc acttatcttc acattcatct
6121 acaaattcga attcgaaacc ttcgactgca tcaccatcgg cccatacgtc atcttctgac
6181 ggtaataacg aaatatttac gtgtcagata atgaatctca ttacaaatga accgtgtggt
6241 gcccaatttt caaggtccta tgatttaacg agacaccaaa ataccattca cgctaaaagg
6301 aagattgtct tccgttgctc ggagtgtata aaaattcttg gatctgaggg ctatcagaag
6361 acgttttcga gactggatgc tttaacaagg catataaaat cgaagcatga agatttgtcg
6421 ttagaacaac gtcaagaagt tacaaaattt gcaaaggcta atattggtta tgtcatgggt
6481 taaattatat aggagataaa agaagaaaac ctcacacaaa atggaaatac atatacacat
6541 ctatatatat ttacaatata tatcatatat ctgcattcag gaatgttctt ataaatatca
6601 catttttaaa gtacctcatt ggacttataa taagttttct attggttttc attatacttc
6661 ggaaaataca caattatatt atatacttac ccccccttaag gatttttttat gaaaccctca
6721 ttacggactt tctcagcatt gatcttagca gattcaggat ctaggtagaa ttttctgata
6781 aacttaagac cattattctt atctaattca acgactaagg ggataccatt tggaatatca
6841 acattcttga tgtcatcatc tgatataccc tccagaattt tcagtagcga tctcactgaa
6901 cttccatggc ccacaatcag gcatgaagat tcatcatcgt cctgcaggac gatggcttct
6961 tatctcactt caatagtact ttccaccggt tatacttccg gcttttccct attaatacaa
7021 gctacaattt caatgggtgg caaataatgt gtagaataga aaataagccg acagggtaat

7081 aaagaaaatt tttagaaaaa aaaggttaga tggcttattt aagttacagg ctagcgaaaa
7141 aaggaacttc agggcaagta aagtgtttga ttgggcacta gcatggctta taaaggcgag
7201 caattgtcga aactaattaa tgttgtacgg actattgctg tcatctcgtg gtaaatgcgt
7261 gttccaggtc gaatactact tgcacacagg cgagcggggc cccataaaag tgttgccgat
7321 ttgttaagtt gtcttttcgg tttttctact ctgttattcc ttacttccct ttttaagaac
7381 tctttttatc cttcatttag gatcttgcac gtttccgcct catcacttga attaaaacat
7441 gtctctgtca gtaaaccttg gcgtttctat tgttcttcat agttcaactt ttattattac
7501 ccgccctgcg cgtttacatt tttccagcaa cagccagcga aaaattagaa aatctggttg
7561 ttgacacctc aagaacaagg gcaattagcc tcagcgtcga atatagatca tattagaata
7621 cctatagctc catcaaaaga aatacacaag atctatgggt acccgcccat atgcttgccc
7681 tgtcgagtcc tgcgatcgcc gcttttctcg ccacgccaat cttacccgcc atatccgcat
7741 ccataccggt cagaagccct tccagtgtcg aatctgcatg cgtaacttca gtcgtaatgc
7801 gaaccttgtg cgccacatcc gcacccacac aggatcccaa aagccgttcc aatgtcggat
7861 ctgtatgcgg aactttagtc gaaaggccga cctgaggcgt cacattcgca cgcacaccgg
7921 cgagaagcct tttgcctgtg acatttgtgg gaggaagttt gccaggaagg gcgacctcaa
7981 gaggcatacc aaaatccata caggtagatc cggtgacggt gctggtttaa ttaactctgc
8041 tggagacatg agagctgcca acctttggcc aagcccgctc atgatcaaac gctctaagaa
8101 gaacagcctg gccttgtccc tgacggccga ccagatggtc agtgccttgt tggatgctga
8161 gcccccccata ctctattccg agtatgatcc taccagaccc ttcagtgaag cttcgatgat
8221 gggcttactg accaacctgg cagacaggga gctggttcac atgatcaact gggcgaagag
8281 ggtgccaggc tttgtggatt tgaccctcca tgatcaggtc caccttctag aatgtgcctg
8341 gctagagatc ctgatgattg gactcgtctg gcgctccatg gagcacccag ggaagctact
8401 gtttgctcct aacttgctct tggacaggaa ccagggaaaa tgtgtagagg gcatggtgga
8461 gatcttcgac atgctgctgg ctacatcatc tcggttccgc atgatgaatc tgcagggaga
8521 ggagtttgtg tgcctcaaat ctattatttt gcttaattct ggagtgtaca catttctgtc
8581 cagcaccctg aagtctctgg aagagaagga ccatatccac cgagtcctgg acaagatcac
8641 agacactttg atccacctga tggccaaggc aggcctgacc ctgcagcagc agcaccagcg

8701 gctggcccag ctcctcctca tcctctccca catcaggcac atgagtaaca aaggcatgga
8761 gcatctgtac agcatgaagt gcaagaacgt ggtgcccctc tatgacctgc tgctggagat
8821 gctggacgcc caccgcctac atgcgcccac tagccgtgga ggggcatccg tggaggaaac
8881 ggaccaaagc cacttggcca ctgcgggctc tacttcatcg cattccttgc aaaagtatta
8941 catcacgggg gaggcagagg gtttccctgc cacagtcgcg gctgcaggtg acggtgctgg
9001 tttaattaac atgacggtcg accatgattt caatagcgaa gatattttat tccccataga
9061 aagcatgagt agtatacaat acgtggagaa taataaccca aataatatta acaacgatgt
9121 tatcccgtat tctctagata tcaaaaacac tgtcttagat agtgcggatc tcaatgacat
9181 tcaaaatcaa gaaacttcac tgaatttggg gcttcctcca ctatctttcg actctccact
9241 gcccgtaacg gaaacgatac catccactac cgataacagc ttgcatttga aagctgatag
9301 caacaaaaat cgcgatgcaa gaactattga aaatgatagt gaaattaaga gtactaataa
9361 tgctagtggc tctggggcaa atcaatacac aactcttact tcaccttatc ctatgaacga
9421 cattttgtac aacatgaaca atccgttaca atcaccgtca ccttcatcgg tacctcaaaa
9481 tccgactata aatcctccca taaatacagc aagtaacgaa actaatttat cgcctcaaac
9541 ttcaaatggt aatgaaactc ttatatctcc tcgagcccaa caacatacgt ccattaaaga
9601 taatcgtctg tccttaccta atggtgctaa ttcgaatctt ttcattgaca ctaacccaaa
9661 caatttgaac gaaaaactaa gaaatcaatt gaactcagat acaaattcat attctaactc
9721 catttctaat tcaaactcca attctacggg taatttaaat tccagttatt ttaattcact
9781 gaacatagac tccatgctag atgattacgt ttctagtgat ctcttattga atgatgatga
9841 tgatgacact aatttatcac gccgaagatt tagcgacgtt ataacaaacc aatttccgtc
9901 aatgacaaat tcgaggaatg agctcggatc ctaacttaag tctgaagaat gaatgatttg
9961 atgatttctt tttccctcca tttttcttac tgaatatatc aatgatatag acttgtatag
10021 tttattattt caaattaagt agctatatat agtcaagata acgtttgttt gacacgatta
10081 cattattcgt cgacatcttt tttcagcctg tcgtggtagc aatttgagga gtattattaa
10141 ttgaataggt tcattttgcg ctcgcataaa cagttttcgt cagggacagt atgttggaat
10201 gagtggtaat taatggtgac atgacatgtt atagcaataa ccttgatgtt tacatcgtag
10261 tttaatgtac accccgcgaa ttcgttcaag taggagtgca ccaattgcaa agggaaaagc

10321 tgaatgggca gttcgaaccg cggtctttcc tgcgttatcc cctgattctg tggataaccg
10381 tattaccgcc tttgagtgag ctgataccgc tcgccgcagc cgaacgaccg agcgcagcga
10441 gtcagtgagc gaggctgagc gacatggagg cccagaatac cctccttgac agtcttgacg
10501 tgcgcagctc aggggcatga tgtgactgtc gcccgtacat ttagcccata catccccatg
10561 tataatcatt tgcatccata cattttgatg gccgcacggc gcgaagcaaa aattacggct
10621 cctcgctgca gacctgcgag cagggaaacg ctcccctcac agacgcgttg aattgtcccc
10681 acgccgcgcc cctgtagaga aatataaaag gttaggattt gccactgagg ttcttctttc
10741 atatacttcc ttttaaaatc ttgctaggat acagttctca catcacatcc gaacataaac
10801 aaccatgggt aaggaaaaga ctcacgtttc gaggccgcga ttaaattcca acatggatgc
10861 tgatttatat gggtataaat gggctcgcga taatgtcggg caatcaggtg cgacaatcta
10921 tcgattgtat gggaagcccg atgcgccaga gttgtttctg aaacatggca aaggtagcgt
10981 tgccaatgat gttacagatg agatggtcag actaaactgg ctgacggaat ttatgcctct
11041 tccgaccatc aagcatttta tccgtactcc tgatgatgca tggttactca ccactgcgat
11101 ccccggcaaa acagcattcc aggtattaga agaatatcct gattcaggtg aaaatattgt
11161 tgatgcgctg gcagtgttcc tgcgccggtt gcattcgatt cctgtttgta attgtccttt
11221 taacagcgat cgcgtatttc gtctcgctca ggcgcaatca cgaatgaata acggtttggt
11281 tgatgcgagt gattttgatg acgagcgtaa tggctggcct gttgaacaag tctggaaaga
11341 aatgcataag cttttgccat tctcaccgga ttcagtcgtc actcatggtg atttctcact
11401 tgataacctt atttttgacg aggggaaatt aataggttgt attgatgttg gacgagtcgg
11461 aatcgcagac cgataccagg atcttgccat cctatggaac tgcctcggtg agttttctcc
11521 ttcattacag aaacggcttt ttcaaaaata tggtattgat aatcctgata tgaataaatt
11581 gcagtttcat ttgatgctcg atgagttttt ctaatcagta ctgacaataa aaagattctt
11641 gttttcaaga acttgtcatt tgtatagttt ttttatattg tagttgttct attttaatca
11701 aatgttagcg tgatttatat ttttttcgc ctcgacatca tctgcccaga tgcgaagtta
11761 agtgcgcaga aagtaatatc atgcgtcaat cgtatgtgaa tgctggtcgc tatactggcg
11821 gccgccctat agtgagtcgt attagagtta ttacccgagt agagcacttg aatccactgc
11881 cccgggaatc tcggtcgtaa tgatttctat aatgacgaaa aaaaaaaaat tggaaagaaa

11941 aagcttcatg gcctttataa aaaggaacta tccaatacct cgccagaacc
aagtaacagt
12001 attttacggg gcacaaatca agaacaataa gacaggactg taaagatgga
cgcattgaac
12061 tccaaagaac aacaagagtt ccaaaaagta gtggaacaaa agcaaatgaa
ggatttcatg
12121 cgtttgtact ctaatctggt agaaagatgt ttcacagact gtgtcaatga
cttcacaaca
12181 tcaaagctaa ccaataagga acaaacatgc atcatgaagt gctcagaaaa
gttcttgaag
12241 catagcgaac gtgtagggca gcgtttccaa gaacaaaacg ctgccttggg
acaaggcttg
12301 ggccgataag gtgtactggc gtatatatat ctaattatgt atctctggtg
tagcccattt
12361 ttagcatgta aatataaaga ccgagcgggc cggcctcggg ccatggtcac tg
//
